# Novel Microcystins from *Planktothrix prolifica* NIVA-CYA 544 Identified by LC-MS/MS, Functional Group Derivatization and ^15^N-labeling

**DOI:** 10.3390/md17110643

**Published:** 2019-11-15

**Authors:** Vittoria Mallia, Silvio Uhlig, Cheryl Rafuse, Juris Meija, Christopher O. Miles

**Affiliations:** 1 Toxinology Research Group, Norwegian Veterinary Institute, Ullevålsveien 68, N-0454 Oslo, Norway; silvio.uhlig@vetinst.no; 2 Department of Chemistry, University of Oslo, P.O. Box 1033, N-0315 Oslo, Norway; 3 National Research Council, 1411 Oxford Street, Halifax, NS B3H 3Z1, Canada; cheryl.rafuse@nrc-cnrc.gc.ca (C.R.); christopher.miles@nrc-cnrc.gc.ca (C.O.M.); 4 National Research Council, 1200 Montreal Road, Ottawa, ON K1A 0R6, Canada; juris.meija@nrc-cnrc.gc.ca

**Keywords:** cyanotoxin, microcystin, hepatotoxin, mass spectrometry, *Planktothrix*

## Abstract

Microcystins are cyclic heptapeptides from cyanobacteria that are potent inhibitors of protein phosphatases and are toxic to animals and humans. At present, more than 250 microcystin variants are known, with variants reported for all seven peptide moieties. While d-glutamic acid (d-Glu) is highly-conserved at position-6 of microcystins, there has been only one report of a cyanobacterium (*Anabaena*) producing microcystins containing l-Glu at the variable 2- and 4-positions. Liquid chromatography–mass spectrometry analyses of extracts from *Planktothrix prolifica* NIVA-CYA 544 led to the tentative identification of two new Glu-containing microcystins, [d-Asp^3^]MC-ER (**12**) and [d-Asp^3^]MC-EE (**13**). Structure determination was aided by thiol derivatization of the Mdha^7^-moiety and esterification of the carboxylic acid groups, while ^15^N-labeling of the culture and isotopic profile analysis assisted the determination of the number of nitrogen atoms present and the elemental composition of molecular and product-ions. The major microcystin analog in the extracts was [d-Asp^3^]MC-RR (**1**). A microcystin with an unprecedented high-molecular-mass (2116 Da) was also detected and tentatively identified as a sulfide-linked conjugate of [d-Asp^3^]MC-RR (**15**) by LC–HRMS/MS and sulfide oxidation, together with its sulfoxide (**16**) produced via autoxidation. Low levels of [d-Asp^3^]MC-RW (**14**), [d-Asp^3^]MC-LR (**4**), [d-Asp^3^,Mser^7^]MC-RR (**11**), [d-Asp^3^]MC-RY (**17**), [d-Asp^3^]MC-RF (**18**), [d-Asp^3^]MC-RR–glutathione conjugate (**19**), and [d-Asp^3^]MC-RCit (**20**), the first reported microcystin containing citrulline, were also identified in the extract, and an oxidized derivative of [d-Asp^3^]MC-RR and the cysteine conjugate of **1** were partially characterized.

## 1. Introduction

**Figure 1:**
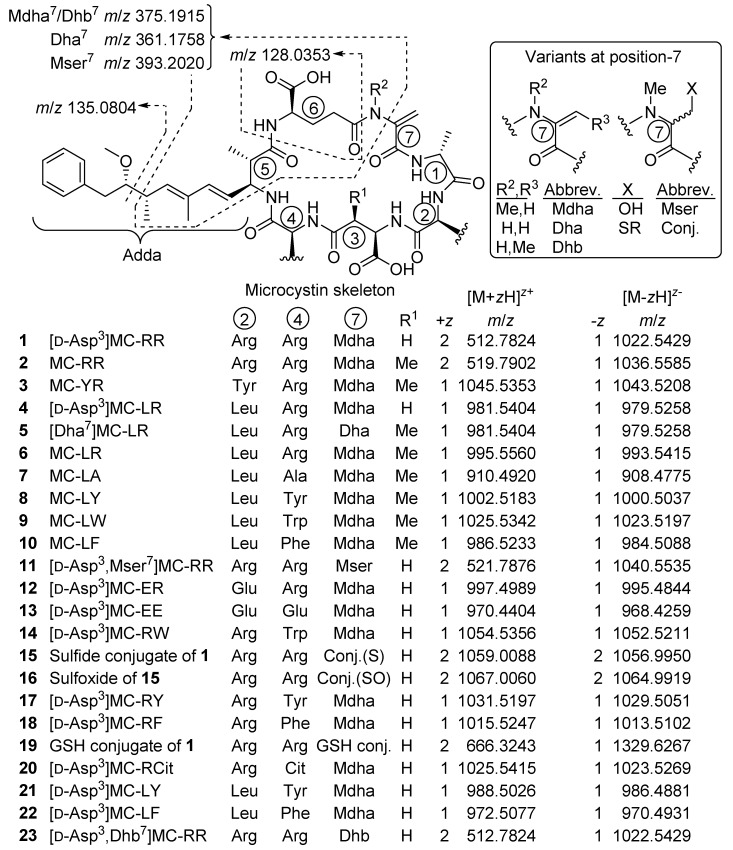
Structures of microcystins (MCs) mentioned in the abstract and text. Values for *m*/*z* are exact masses except for **15** and **16**, for which definitive atomic compositions have not yet been established. The origin of the characteristic fragments from Adda^5^ and Glu^6^ (in positive and negative ionization modes, respectively) are also shown. The stereochemistries of **11**–**16** and **20** are assumed, based on biosynthetic considerations, and amino acid numbering is shown inside the circles. An oxidized derivative of **1** and a cysteine conjugate of **1** was also tentatively identified in NIVA-CYA 544 extracts.

Microcystins (MCs) (Figure 1) are non-ribosomal heptapeptides [1] produced by cyanobacteria, frequently occurring in eutrophic freshwater ecosystems worldwide [2,3]. MCs are potent hepatotoxins implicated in the poisoning of diverse birds, fish, and mammals, including sheep, dogs, cattle, sea otters, and humans [4,5], and one incident with human fatalities has been reported [6,7]. Inhibition of protein phosphatase-1 and -2A (PP1 and PP2A) is believed to be the principal mechanism of toxicity of MCs [8,9]. Some studies show that MCs can also modulate PP activity by regulating their expression [10]. Oxidative stress may also be an important additional biochemical mechanism of MC toxicity in both mammalian and plant cells [11,12]. Recent studies have implicated MCs as reproductive toxins, likely due to endocrine-disrupting effects [10]. They are among the most common cyanotoxins worldwide and are the most studied. They are synthesized intracellularly by several cyanobacterial genera including *Microcystis* and *Planktothrix* spp., and then released to water bodies via cell lysis following cell death and/or physical stress [2,13,14,15].

Currently, more than 250 MC variants have been reported [16]. The increasing number of congeners, and the complexity of the sample matrix in environmental samples from mixed cyanobacterial blooms, complicates the detection and identification of MCs [17]. The World Health Organization recommends a provisional guideline value of 1 µg/L for MC-LR, the most studied MC congener, in drinking water and a chronic tolerable daily intake (TDI) of 0.04 µg/kg body mass per day for humans [18]. However, the vast majority of the congeners cannot be monitored in a single targeted LC–MS/MS method (so usually only the most common MCs are targeted), nor are their biological effects well understood [19]. Since the structure of MC congeners influences their toxicities [20], reliable identification of all major MC variants produced by individual cyanobacterial strains or in algal blooms is therefore needed for effective risk assessment and freshwater management [21].

MCs have molecular masses of around 1 kDa and share a general cyclic structure composed of seven d- and l-amino acids, including uncommon amino acids such as 3*S*-amino-9*S*-methoxy-2*S*,6,8*S*-trimethyl-10-phenyldeca-4*E*,6*E*-dienoic acid (Adda), iso-linked d-β-methylaspartic acid (d-Masp) and *N*-methyldehydroalanine (Mdha) (Figure 1). The presence of the Adda residue is crucial for the toxicity of MC molecules with both Adda^5^ and γ-linked d-Glu^6^ being particularly important for binding to the protein phosphatase enzyme [22,23]. The common amino acid sequence in MCs is cyclo(d-Ala^1^-X^2^- d-Masp^3^-Z^4^-Adda^5^-γ-d-Glu^6^-Mdha^7^) (Figure 1), where X and Z are variable l-amino acids. Other frequently encountered variations stem from demethylation or methylation at positions-3 (i.e., d-Asp instead of d-Masp) or -7 (e.g., dehydrobutyrine (Dhb) or dehydroalanine (Dha) instead of Mdha) [21]. In other cases, the substitution of d-Ala^1^ by d-Leu or d-Ser, and methyl esterification at d-Glu^6^ (to form d-Glu(OMe)^6^) have also been observed [24], although such methyl esters appear to be artefactual [25,26]. These structural variations can have a major impact on the physical properties of MCs, as well as on their toxicity and fate during algal bloom events [22]. In addition to MCs, cyanobacteria can also produce other cyanotoxins, other oligopeptides, can contain lipopolysaccharides in their cell walls and may produce other metabolites with various bioactivities and potential applications [16,27,28].

As a prelude to investigations into the components responsible for the reported reproductive toxicity of cyanobacteria [10,29,30], we screened a range of cultures of *Microcystis* and *Planktothrix* strains for MCs by LC–MS because the toxicity of MCs might influence results of cell-based bioassays. Several previously unreported putative MC congeners were detected in *P. prolifica* strain NIVA-CYA 544, isolated from Lake Steinsfjorden, Buskerud, Norway, in 2004. Here we report detailed analysis using LC–MS and MS/MS, chemical reactivity tests, and ^15^N-labeling, leading to the identification of a range of novel and previously known MCs in this culture.

## 2. Results and Discussion

### 2.1. MCs Profiling of P. prolifica NIVA-CYA 544

**Figure 2:**
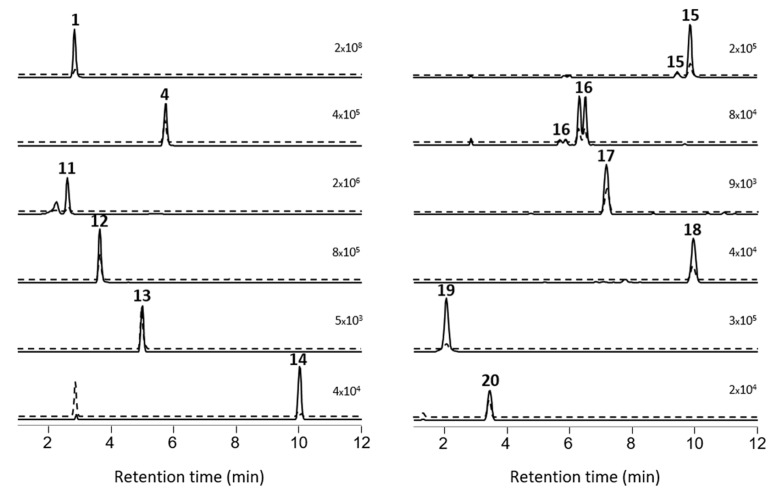
Extracted ion LC–HRMS chromatograms (± 5 ppm) of MC congeners obtained with LC-HRMS/MS method A from a concentrated extract of *P. prolifica* NIVA-CYA 544 (from top left): [d-Asp^3^]MC-RR (**1**), [d-Asp^3^]MC-LR (**4**), [d-Asp^3^,Mser^7^]MC-RR (**11**), [d-Asp^3^]MC-ER (**12**), [d-Asp^3^]MC-EE (**13**), [d-Asp^3^]MC-RW (**14**), sulfide-conjugate of **1** (**15**), sulfoxide of **15** (**16**), [d-Asp^3^]MC-RY (**17**), [d-Asp^3^]MC-RF (**18**), GSH-conjugate of **1** (**19**), and [d-Asp^3^]MC-RCit (**20**). Solid lines are chromatograms from positive ionization ([M + H]^+^ or [M + 2H]^2+^), while dashed lines are from negative ionization ([M − H]^−^ or [M − 2H]^2−^). Each pair of the positive and negative chromatogram is on the same fixed intensity scale (number in the top right-hand corner of each chromatogram).

Prior to the profiling of the *P. prolifica* strain, LC–HRMS (method A) and LC–ITMS/MS (method C) were tested and optimized using a set of nine MC standards as well as a nodularin-R standard. Extracts from the culture were then examined by LC–ITMS/MS and LC–HRMS/MS methods in positive and negative ionization modes, and the chromatograms examined for characteristic precursor- and product-ions (including those shown in Figure 1) corresponding to known MCs. To detect possible MC congeners in the *P. prolifica* extract, we also specifically looked for the Adda^5^-derived product-ion at *m*/*z* 135.0804 (Figure 1) in the positive mode HRMS/MS spectra, as well as the *m*/*z* 128.0353 (or 129.0324 in ^15^N-labeled MCs) product-ion (Figure 1), derived from the d-Glu^6^ moiety of MCs, in negative mode HRMS/MS spectra. The positive mode HRMS/MS spectra were also examined for a range of other characteristic product-ions of MCs. All of the candidate MCs displayed product-ions indicative of the presence of Adda^5^ and d-Glu^6^ in their HRMS/MS mass spectra. In addition, derivatization with mercaptoethanol was used together with LC–HRMS to identify candidate peaks of thiol-reactive compounds potentially containing Dha^7^- or Mdha^7^-groups [31,32], and identified 8 of the 12 candidate MC peaks (Figure 2, Table 1) as potentially containing Dha^7^ or Mdha^7^ moieties. Together, these screening approaches target three of the MC amino acid residues in closest contact with the binding site of PPs, two of which (Adda^5^ and d-Glu^6^) appear to be required for inhibition of PPs by MCs [33]. The resulting candidate peaks from this screening were then matched with possible precursor ions with the same retention time and an appropriate *m*/*z* in the LC–HRMS chromatograms (Figure 2), and more concentrated extracts were studied by targeted LC–HRMS/MS analysis, chemical reactivity, and ^15^N-labeling. Extracts were also treated with sodium periodate to identify compounds containing sulfide linkages via oxidation to their sulfoxides [25,33,34] with the reactions monitored by LC–HRMS/MS, and esterified with diazomethane to count the number of reactive carboxylic acid groups present in each MC, with the reactions monitored by LC–HRMS/MS.

### 2.2. Isotopic Enrichment Calculations

**Figure 3:**
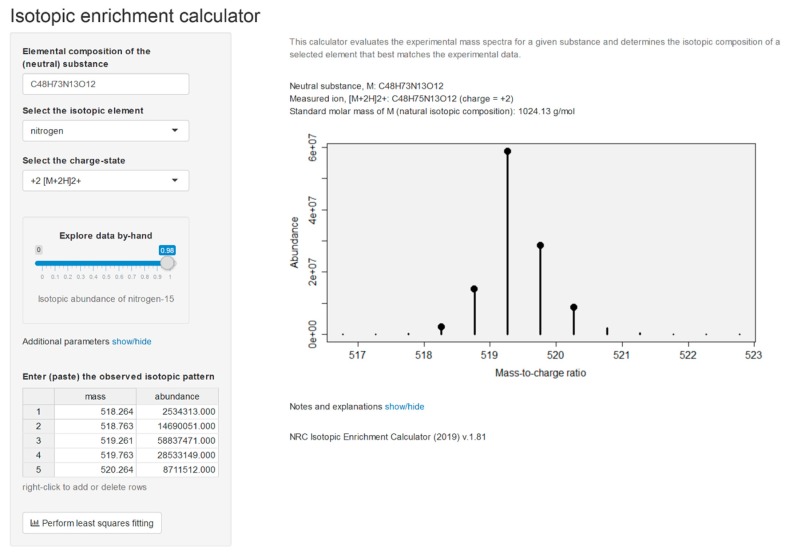
Screenshot of the NRC Isotopic Enrichment Calculator (v.1.81) as applied to the positive ionization electrospray mass spectrum for [d-Asp^3^]MC-RR (**1**) grown in a medium having 98% of nitrogen as nitrogen-15. Data input is in the left panel with output on the right-hand panel including the isotopic abundance with the best fit to the observed *m*/*z* and intensities, together with a representation of the mass spectrum (circles: observed *m*/*z* and intensities, vertical bars: calculated values given the molecular formula, charge-state, and isotopic composition of nitrogen).

The culture was maintained for 13 months in ^15^N-labeled medium and analyzed alongside unlabeled culture, allowing the number of N-atoms in the molecular- and product-ions to be determined [35] using LC–HRMS/MS method B. Isotopic composition of the constituent elements dictates the shape of isotope patterns observed in mass spectrometry. A molecule with elemental formula C*_c_*H*_h_*N*_n_*O*_o_*, for example, will contain (1 + *c*)(1 + *h*)(1 + *n*)(1 + *o*)(2 + *o*)/2 distinct combinations of ions (isotopologues). The abundance (proportion) of all of these isotopologues is governed in accordance with the basic rules of probability. For example, with the lightest of the ions having an abundance of *x*(^12^C)*^c^x*(^1^H)*^h^x*(^14^N)*^n^x*(^16^O)*^o^* where *x*(^12^C) is the abundance (proportion) of carbon-12 atoms among all carbon atoms and so on. Thus, knowledge of the molecular formula and the isotopic composition of all makeup elements enables us to establish the expected (‘theoretical’) isotope patterns of molecules. The full isotopic pattern of the MC [d-Asp^3^]MC-RR (**1**) (C_48_H_73_N_13_O_12_) contains nearly 5 million components, which necessitates computationally efficient algorithms to be used in practical calculations. When the isotopic composition of nitrogen (the abundance of nitrogen-15, *x*(^15^N)) is unknown in the analyzed toxins, the theoretical isotopic patterns can be viewed as a function of *x*(^15^N) and both the experimental and theoretical patterns are compared for each plausible value of *x*(^15^N) until the best fit is obtained. The similarity between the experimental and theoretical spectra is evaluated by comparing the ion intensities at all unique masses. For this, all ions in theoretical spectra are aggregated similar to the data collection process of the mass spectrometer. A good match between the two spectra will exhibit a linear regression, *I*_theor_(M*_i_*) = *bI*_exp_(M*_i_*), and the isotopic enrichment of nitrogen corresponding to the best match between the two spectra was found by means of the correlation coefficient of the above regression as described by MacCoss et al. [36]. Although lighter ions tend to have higher ion transmission efficiencies in mass spectrometers [37], leading to slightly biased isotopic patterns, an effect known as the instrumental mass fractionation, this has little effect on our calculations. Moreover, the fitness-for-purpose of our approach was established by subjecting our calculation routines to a series of MCs of known identity (molecular formula). The calculations were performed in R using a web-based interface and an example is shown for [d-Asp^3^]MC-RR (**1**) (Figure 3).

### 2.3. Elemental Composition Elucidation

**Figure 4:**
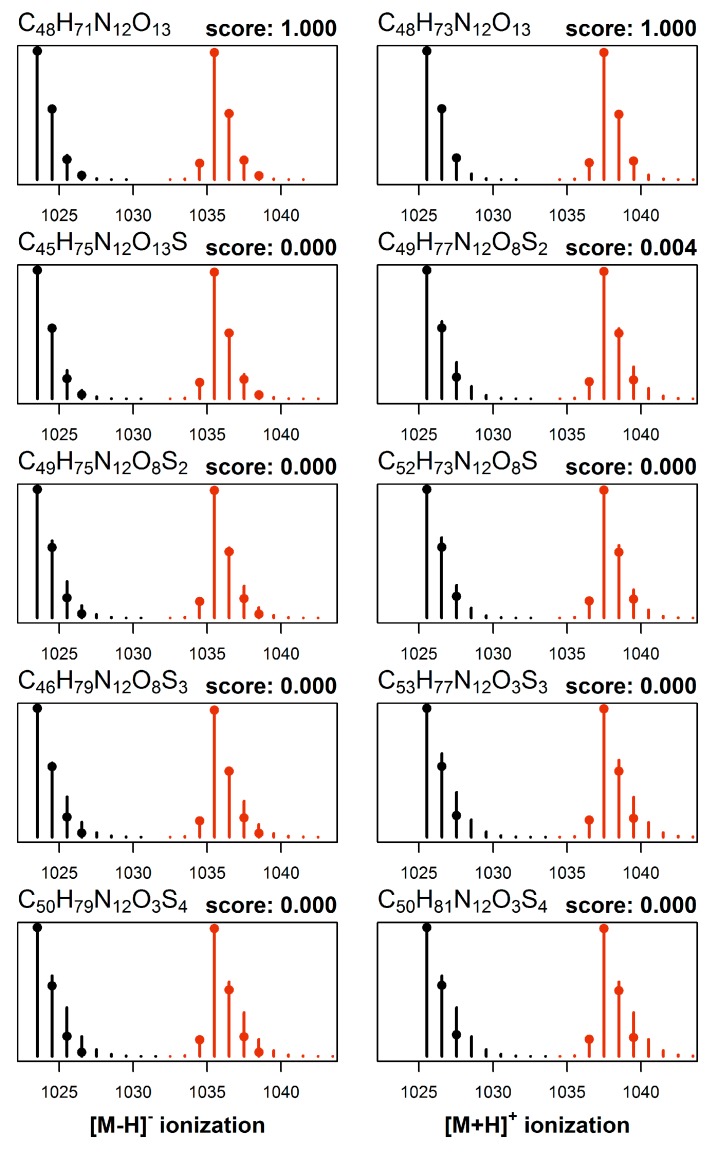
Elucidation of the elemental formula from HRMS of an MC using the NRC Molecular Formula Calculator. This example shows normalized mass spectra ([M − H]^−^ on the left, [M + H]^+^ on the right) of **20** from a culture cultivated in normal growth medium and in medium whose isotopic composition of nitrogen was altered to 98% nitrogen-15. Each MS measurement mode produced mass spectra (*m*/*z* and intensities shown with circles) from natural (black) and nitrogen-15 enriched (red) growth media, which were subjected to molecular formula elucidation calculations. Assuming up to 3 ppm mass measurement errors, only five elemental formulae satisfied all constraints (higher scoring formulae shown from top to bottom), and the resulting matches along with their match-scores (from 0.000–1.000) are shown (calculated mass spectra shown as black or red bars). Only one plausible candidate emerged with a high score for both [M − H]^−^ and [M + H]^+^ (C_48_H_72_N_12_O_13_), which is the elemental formula for [d-Asp^3^]MC-RCit (**20**). The remaining candidates showed very low scores for both [M − H]^−^ and [M + H]^+^, indicating a poor match to the experimentally observed HRMS spectra, and that they are therefore not viable elemental formulae for **20**.

The cultures were grown in two distinct media, one having normal isotopic composition and the other enriched in nitrogen-15. Crude extracts from both cultures were then mixed and analyzed by LC–HRMS/MS method B, which provided a set of two mass spectra obtained under identical conditions for each toxin. The molecular formulae were elucidated from this set of data first, by performing mass decomposition of each observed signal using efficient algorithms as implemented in the R packages ecipex [38] and Rdisop [39,40] while taking into account the isotopic enrichment of nitrogen-15 in the MCs as determined from the analysis of **1** present in the same cultures. From the set of obtained matches we retained only the molecular formulae common to all mass signals, and further eliminated those formulae that violated the Senior rules of molecular composition [41]. The theoretical isotopic pattern was generated for each candidate match and formulae whose theoretical isotopic patterns deviated significantly from the observed isotopic patterns were discarded. Last, the two resulting sets of candidates (one from the natural growth medium and one from nitrogen-15 enriched growth medium) were intersected and common matches returned. The entire procedure was then repeated for data acquired under different ionization modes, so that all the data available (e.g., [M + H]^+^, [M + 2H]^2+^, and [M − H]^−^) were used together to constrain the set of molecular formula candidates for each compound (e.g., Figure 4). The calculations were performed in R using a web-based interface, and a graphical representation of the output is shown for [d-Asp^3^]MC-RCit (**20**) (Figure 4). In this case, the analysis of *m*/*z* values of the observed signals from the neutral molecule leads to ca. 2000 candidate formulae which are reduced to ca. 200 by applying Senior rules. The isotopic profile analysis reduces the number of molecular formulae candidates to ca. 100 and then to 5 remaining matches after cross-referencing them with expected isotopic patterns of isotopically labeled analogs.

### 2.4. Identification of MC Congeners

Individual compounds were tentatively identified as MCs based on their MS/MS spectra, retention times relative to authentic standards and thiol reactivity. Tentative structures were then assigned based on the molecular formulae established from LC–HRMS, LC–ITMS/MS, LC–HRMS/MS and ^15^N-labeling experiments, as well as reactivity towards thiols, diazomethane, and periodate. It should be noted that the stereochemistry of the compounds cannot be determined using MS data alone, and the stereochemistries of novel compounds **12**–**16** and **20** were assumed to be identical to those of known MCs based on biosynthetic considerations (MCs are produced by MC synthetases, and all MCs whose structures have been fully elucidated possess the amino acid stereochemistry shown in Figure 1).

[d-Asp^3^]MC-RR (**1**): In LC–HRMS, the most abundant compound afforded ions with *m*/*z* 512.7815 (Table 1) and 1024.5549 (*z* = 2 and 1, respectively) in positive, and *m*/*z* 1022.5437 in negative ion modes, and contained 13 N atoms by ^15^N-labeling experiments, corresponding to an elemental composition of C_48_H_73_N_13_O_12_ for the neutral molecule (Table 1, Figure 1). This, as well as its short retention time and the predominance of its double-charged molecular ion in positive ion mode, was indicative of a desmethylated MC-RR congener. In the initial studies, **1** from NIVA-CYA 544 unexpectedly showed a small but consistent difference in retention time compared to a standard of [d-Asp^3^]MC-RR using method A (Figure S1), even though the two compounds gave essentially identical LC–HRMS/MS product-ion spectra. However, comparison of the reactivity of these compounds with mercaptoethanol and LC–HRMS/MS characteristics with reference materials of [d-Asp^3^]MC-RR (**1**) and [d-Asp^3^,Dhb^7^]MC-RR (**23**) from NRC, showed that **1** from NIVA-CYA 544 possessed identical retention time, MS and MS/MS spectra and rapid thiol reactivity as **1** from NRC, establishing its identity as **1**. In contrast, the initially-used commercial standard of [d-Asp^3^]MC-RR did not react detectably with mercaptoethanol, characteristic of the presence of a Dhb^7^- or Mdhb^7^-containing MC [31], and its retention time, MS, MS/MS spectra and thiol-reactivity characteristics were identical to those of the [d-Asp^3^,Dhb^7^]MC-RR (**23**) reference material from NRC. Product-ions from higher-energy collisional dissociation (HCD) of **1** in positive ion mode at *m*/*z* 375.1901 (C_20_H_27_O_5_N_2_^+^, from Adda^5^–d-Glu^6^–Mdha^7^ minus C_9_H_10_O, Δ*m* = −3.6 ppm, Figure 1) and 426.2077 (C_17_H_28_O_6_N_7_^+^, from Mdha^7^–d-Ala^1^–Arg^2^–d-Asp^3^, Δ*m* = −4.4 ppm) confirmed the site of demethylation as being on position-3 rather than position-7 (i.e., d-Asp^3^ rather than Dha^7^). This compound has previously been identified as the predominant MC in this strain [31]. Treatment of an extract of NIVA-CYA 544 with diazomethane resulted in complete esterification of the d-Glu^6^ carboxylic acid group of **1**, but no esterification of the d-Asp^3^ residue was detected. The d-Asp^3^ residue appears to be relatively unreactive to acid-catalyzed esterification with methanol since d-Glu(OMe)^6^ but not d-Asp^3^ esters have been reported as esterification artifacts thus far [25,26], and the (trimethylsilyl)diazomethane-promoted methyl esterification of MCs was recently shown to display the same selectivity [42]. With the identity of **1** as the most abundant MC in NIVA-CYA 544 firmly established, analysis of the profile of its molecular ion isotope envelope was used to estimate the level of nitrogen-15 incorporation into MCs at *x*(^15^N) = 0.98 mol/mol (Figure 3) in NIVA-CYA 544 after extended maintenance of the culture in nitrogen-15 enriched medium.

[d-Asp^3^,Mser^7^]MC-RR (**11**): The peak of [d-Asp^3^]MC-RR (**1**) was accompanied by an earlier eluting, minor peak (approximately 1% relative peak area) (Figure 2). In LC–HRMS, the compound afforded ions with *m*/*z* 521.7879 and 1042.5678 (*z* = 2 and 1, respectively) in positive, and *m*/*z* 1040.5567 in negative ion modes, and contained 13 N atoms by ^15^N-labeling, and had an elemental composition of C_48_H_75_N_13_O_13_ for the neutral molecule (Table 1). The mass difference was equivalent to addition of H_2_O to **1** (Table 1), and the compound afforded almost exclusively double-charged ions in positive ion mode, suggesting it to be a desmethylated congener of [Mser^7^]MC-RR (**11**). Furthermore, **11** did not react with mercaptoethanol, indicating that it did not contain an electrophilic double bond such as is present in the Dha^7^ or Mdha^7^ moieties found in most MCs [31,32]. In addition, characteristic product-ions at *m*/*z* 393.2003 (C_20_H_29_O_6_N_2_^+^, from Adda^5^–d-Glu^6^–Mser^7^ minus C_9_H_10_O, Δ*m* = −4.4 ppm, Figure 1) and *m*/*z* 444.2190 (C_17_H_30_O_7_N_7_^+^, from Mser^7^–Ala^1^–Arg^2^–Asp^3^, Δ*m* = −2.5 ppm) indicated the presence of d-Asp^3^ and Mser^7^ moieties, confirming its identity as [d-Asp^3^,Mser^7^]MC-RR (**11**).

[d-Asp^3^]MC-RR conjugate (**15**): One of the more abundant of the minor MCs in terms of peak area in the LC–HRMS chromatograms (Figure 2) of fresh extracts afforded exclusively double-charged ions in positive and negative ion modes with *m*/*z* 1059.0076 and *m*/*z* 1056.9981, respectively (Table 1, Figure 2), which showed weak product-ions at *m*/*z* 135.0804 and 128.0351 in the positive and negative ion LC–HRMS/MS chromatograms, respectively. These data suggest an MC with a molecular mass of 2116 Da for the corresponding neutral molecule. Furthermore, ^15^N-labeling indicated the presence of 21 N atoms in the structure. The compound did not react with mercaptoethanol and was oxidized with periodate to give a product with *m*/*z* corresponding to addition of one oxygen atom, presumed to be the sulfoxide derivative (see discussion for **16**, below, Table 1, Figure 2, Figure S2). The latter two observations suggest a sulfide linkage at the Mdha moiety, since sulfide-containing MCs are readily oxidized to sulfoxides upon treatment with mild oxidants such as periodate [33] or hydrogen peroxide [25], and the presence of an existing sulfide linkage to a Mdha^7^/Dha^7^ would prevent reaction with mercaptoethanol [25,32]. Positive ion LC–HRMS/MS spectra of **15** (and comparison with data from ^15^N-labeled **15**) established the presence of product-ions at *m*/*z* 135.0803 (C_9_H_11_O^+^, Δ*m* = −1.0 ppm, from Adda^5^) and 512.7820 (C_48_H_75_O_12_N_13_^+^, Δ*m* = −0.7 ppm, [**1** + 2H]^2+^), 426.2094 (C_17_H_28_O_6_N_7_^+^, Δ*m* = −0.4 ppm, from Mdha^7^–d-Ala^1^–Arg^2^–d-Asp^3^), and 599.3541 (C_31_H_47_O_6_N_6_^+^, Δ*m* = −1.8 ppm, from Arg^4^–Adda^5^–d-Glu^6^), indicating that **15** is an unidentified sulfide-linked conjugate of **1** coupled via its Mdha^7^ moiety and with a molecular mass of 2116 Da. Analysis of the isotope profile for unlabeled and ^15^N-labeled **15** suggested a probable elemental composition of C_93_H_145_N_21_O_33_S (Table 1), although C_97_H_145_N_21_O_28_S_2_ could not be excluded. Assuming conjugation of a sulfide moiety to **1** via its Mdha^7^-group, the former formula would require the thiol-containing moiety to be C_45_H_72_N_8_O_21_S (or C_49_H_72_N_8_O_16_S_2_ for the latter formula for **15**). While little information is available about the moiety in **15** that is conjugated to **1**, the LC–MS data suggest that it must be relatively non-polar and may contain an acidic functional group and not a strongly basic group, since **15** eluted much later than **1** on a C18 LC column (LC–HRMS method B, Table S1) and was doubly-charged in both positive and negative ion modes.

**Figure 5:**
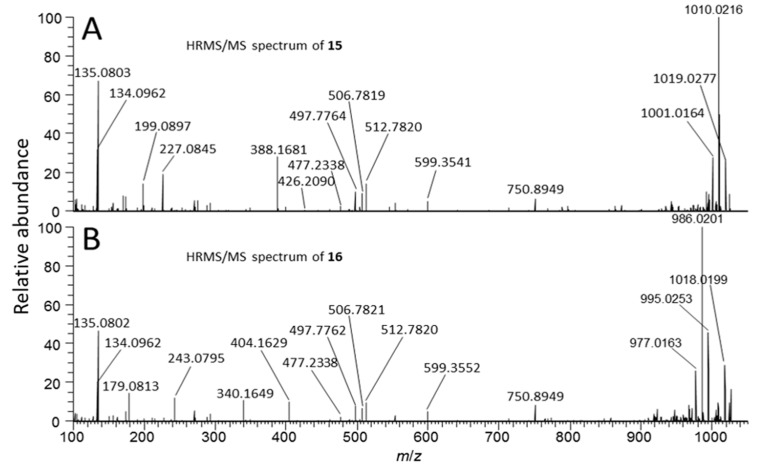
Positive mode product-ion spectra ([M + 2H]^2+^) of putative [d-Asp^3^]MC-RR conjugate **15** (**A**) and its sulfoxide **16** (**B**) obtained from an extract of NIVA-CYA 544 with LC–HRMS/MS method B.

[d-Asp^3^]MC-RR conjugate sulfoxide (**16**): A doubly-charged compound showing a major and a minor peak in both positive and negative modes was present at *m*/*z* 1067.0052 and 1064.9949, respectively, and contained 21 N atoms according to ^15^N-labeling, but was not initially recognized as an MC because it was present at low abundance in extracts, had an unusual charge state given its retention time, and it was not affected by thiol derivatization. However, HP-20 extracts contained this compound at levels sufficient for product-ion spectra, and in LC–HRMS/MS **16** gave rise in positive and negative ion modes to product-ions at *m*/*z* 135.0804 and 128.0351, respectively, that are characteristic of MCs (Figure 1). Furthermore, the mass difference between **16** and **15** corresponded to one oxygen atom, prompting a more detailed examination of **16** and ^15^N-labeled **16** by targeted positive ion LC–HRMS/MS. This confirmed the presence of product-ions at *m*/*z* 135.0804 (C_9_H_11_O^+^, Δ*m* = −0.3 ppm, from Adda^5^) and 512.7816 (C_48_H_75_O_12_N_13_^+^, Δ*m* = −1.5 ppm, from [**1** + 2H]^2+^), 426.2088 (C_17_H_28_O_6_N_7_^+^, Δ*m* = −1.8 ppm, from Mdha^7^–Ala^1^–Arg^2^–Asp^3^), and 599.3550 (C_31_H_47_O_6_N_6_^+^, Δ*m* = −0.3 ppm, from Arg^4^–Adda^5^–d-Glu^6^), indicating a second conjugate of **1**. At this stage, **15** was tested in a periodate oxidation experiment to ascertain whether it contained a dialkyl sulfide that could be oxidized to a sulfoxide. This experiment showed that **15** was quantitatively converted to **16** by periodate oxidation (Figure S2), establishing **16** as an *S*-oxide of **15**. Both peaks of **16** appeared to be distorted in a way that suggested the presence of a partially resolved pair of diastereomeric sulfoxides. Two product-ions in **16** (*m*/*z* 404.1629, C_20_H_26_O_4_N_3_S^+^, Δ*m* −2.4 ppm, and 243.0795, C_10_H_15_O_3_N_2_S^+^, Δ*m* −1.2 ppm) were present at *m*/*z* values 15.9949 greater than the equivalent product-ions in **15** (*m*/*z* 388.1681, C_20_H_26_O_3_N_3_S^+^, Δ*m* −2.2 ppm, and 227.0845, C_10_H_15_O_2_N_2_S^+^, Δ*m* −1.7 ppm) (Figure 5), indicating that these ions contained the sulfide/sulfoxide moiety. Sulfoxide-**16** appears to be an autoxidation product formed through aerial oxidation of **15**, as its concentration relative to **15** increased with time and sample manipulation, and was more abundant in the HP-20 extracts than in simple methanol–water extracts, similar to the situation for Met-containing MCs [34]. Compound-**16** was not affected by the weakly basic ammonium carbonate buffer (pH ~ 8.6) used for the mercaptoethanol derivatization, unlike the glutathione sulfoxide conjugates of [d-Leu^1^]MC-LR reported by Foss et al. [25], which could indicate that the sulfoxide group in **16** (and, consequently, the sulfide of **15**) might not be involved in the conjugation to the Mdha^7^ of **1**.

[d-Asp^3^]MC-RR–glutathione conjugate (**19**): Another predominantly doubly-charged compound, affording positive ions at *m*/*z* 666.3251 and 1331.6461 for [M + 2H]^2+^ and [M + H]^+^, respectively, and negative ions at *m*/*z* 1329.6289 for [M − H]^−^, eluted before **11** and was the earliest eluting MC congener identified with certainty in the extract (Figure 2, Table 1). Its calculated elemental composition (C_58_H_90_N_16_O_18_S for the neutral molecule, Table 1) showed that **19** contained one atom of sulfur and three more nitrogen atoms than **1**. Due to the short retention time and its elemental composition, and that an MC–GSH-conjugate was recently reported in a cyanobacterial bloom [25], we suspected that **19** might be a glutathione conjugate of the major MC congener, **1**. This was verified by reacting a standard of **1** with glutathione and comparing the LC–HRMS characteristics of the products with the culture extract (Figure S15, Figure S39). This appears to be the first report of GSH conjugates of MCs in cyanobacterial culture and suggests that the GSH-derived conjugates identified in a *Microcystis* bloom [25] could have been produced by the cyanobacteria in the bloom without the involvement of other organisms in the water column.

*[d-Asp^3^]MC-LR (**4**).* Another MC afforded [M + H]^+^ and [M − H]^−^ ions at *m*/*z* 981.5419 and *m*/*z* 979.5298 in positive and negative ion modes, respectively (Figure 2, Table 1), while ^15^N-labeling indicated the presence of 10 N atoms in the structure, and an elemental composition of C_48_H_72_N_10_O_12_ for the neutral molecule (Table 1). This analog has the same elemental composition and eluted with the same retention time as a standard of [d-Asp^3^]MC-LR (**4**). Product-ions at *m*/*z* 375.1902 (from Adda^5^–d-Glu^6^–Mdha^7^ minus C_9_H_10_O, Δ*m* = −3.3 ppm, Figure 1), 599.3538 (C_31_H_47_O_6_N_6_^+^, from Arg^4^–Adda^5^–d-Glu^6^, Δ*m* = −2.3 ppm) and 272.1343 (C_10_H_18_O_4_N_5_^+^, Δ*m* = −3.8 ppm, from d-Asp^3^–Arg^4^) showed that demethylation relative to MC-LR was in position-3 and not in position-7, consistent with **4**, which was previously tentatively identified as a minor MC in this culture [31]. Furthermore, detailed examination of the product-ion spectrum obtained from LC–MS^2^ (method C) of **4** (Table 2) showed that it was identical to those reported previously for this compound [32,43,44] and with that of the authentic standard of **4**, and all product-ions containing residue-3 appeared at *m*/*z* values 14 Da less than the corresponding product-ions of MC-LR (**6**) (Table 2).

**Figure 6:**
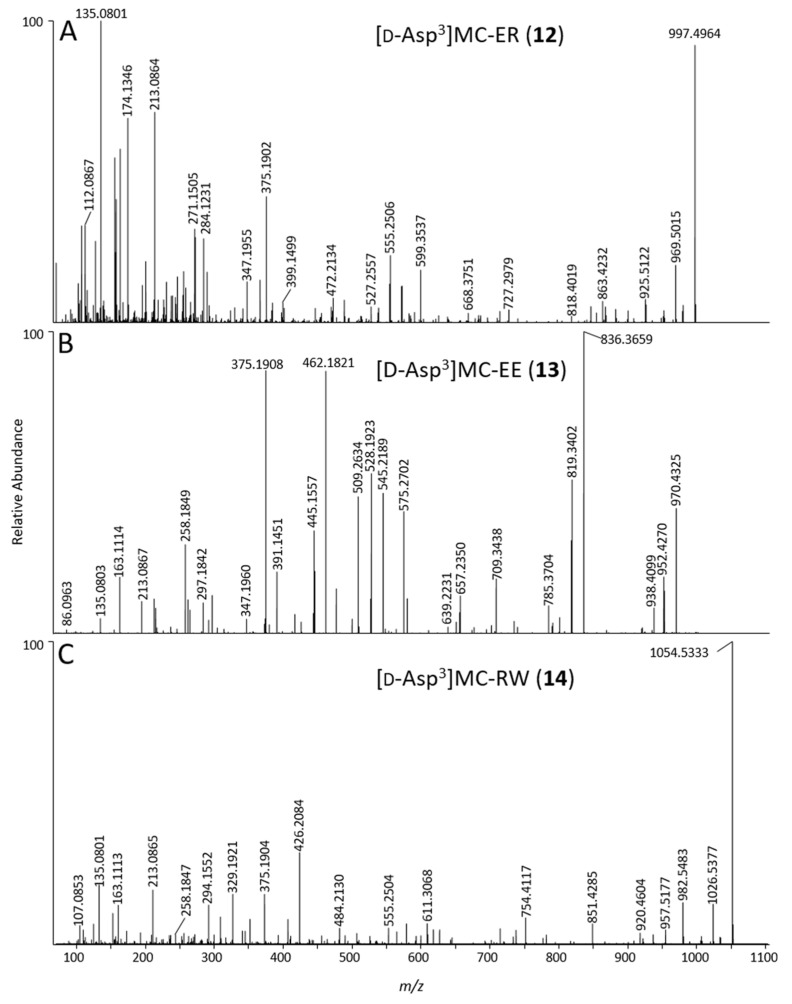
Positive mode product-ion spectra of [d-Asp^3^]MC-ER (**12**) (**A**), [d-Asp^3^]MC-EE (**13**) (**B**) and [d-Asp^3^]MC-RW (**14**) (**C**), obtained from an extract of NIVA-CYA 544 using LC–HRMS/MS (method B).

[d-Asp^3^]MC-ER (**12**): An MC that eluted before [d-Asp^3^]MC-LR (**4**) using LC–MS methods A and B (Table S1), displayed [M + H]^+^ and [M − H]^−^ ions at *m*/*z* 997.4988 and 995.4869 in full-scan positive and negative ion modes, respectively, and contained 10 N atoms by ^15^N-labeling, with an elemental composition of C_47_H_68_N_10_O_14_ (Table 1). The elemental composition, retention time, and being singly-charged was consistent with a demethylated MC containing Glu and Arg in the variable 2- and 4-positions (Figure 1). Product-ions at *m*/*z* 375.1902 (from Adda^5^–d-Glu^6^–Mdha^7^ minus C_9_H_10_O, Δ*m* = −3.3 ppm, Figure 1), 599.3537 (C_31_H_47_O_6_N_6_^+^, Δ*m* = −2.3 ppm, from Arg^4^–Adda^5^–d-Glu^6^) and 155.0811 (C_7_H_11_O_2_N_2_^+^, Δ*m* = −3.3 ppm, from Mdha^7^–d-Ala^1^) indicated that the compound differed from MC-LR (**6**) only in positions-3 and -4 (Figure 6, Figure S22). A product-ion at *m*/*z* 272.1342 (C_10_H_18_O_4_N_5_^+^, Δ*m* = −4.4 ppm, from d-Asp^3^–Arg^4^) indicated demethylation at position-3, showing that the remaining mass difference was in position-2 and thus that the compound likely contained Glu^2^ (Figure S23). Consistent with this was the presence of a product-ion at *m*/*z* 284.1231 (C_12_H_18_O_5_N_3_^+^, Δ*m* = −3.9 ppm, from Mdha^7^–d-Ala^1^–Glu^2^, cf. 268.3365 for Mdha^7^–d-Ala^1^–Leu^2^ for MC-LR (**6**)). In addition, comparison of the product-ion spectrum of **12** with that of [d-Asp^3^]MC-LR (**4**), obtained by LC−ITMS/MS method C, showed that all product-ions containing residue-4 in **12** were heavier by 16 Da than the corresponding product-ions in **4**, while all other product-ions occurred at identical *m*/*z* in **12** and **4**, and the expected mass differences to the corresponding product-ions from **5** and **6** were observed (Table 2). The reaction of **12** with diazomethane gave, principally, a dimethyl ester (Figure S38). This establishes the presence of three carboxylic acid groups in **12** (d-Glu^6^, Glu^2^, and the unreactive d-Asp^3^), and thus that the amino acid at position-2 contains a carboxylic acid rather than a hydroxyketone. Consequently, **12** was determined to be [d-Asp^3^]MC-ER, although the stereochemistry cannot be established from MS/MS data alone. Congeners of MC-ER have not been reported previously, although EE-type MCs have been reported before, but only as their methyl esters [45].

[d-Asp^3^]MC-EE (**13**): An MC affording [M + H]^+^ and [M − H]^−^ ions at *m*/*z* 970.4413 and 968.4301, and containing 7 N atoms by ^15^N-labeling, had a neutral formula of C_46_H_63_N_7_O_16_ (Table 1), indicating the absence of Arg in the structure despite its retention time is only slightly longer than for the Arg^4^-containing **4** in LC–HRMS method A. However, in LC–HRMS method B (using a C18 column) this compound was the latest-eluting MC in NIVA-CYA 544, eluting 4.6 min later than **4**, but more than 3.5 min earlier than non-Arg-containing MCs such as MC-LA in this system (Table S1). The elemental composition was consistent with [d-Asp^3^]MC-EE, an MC containing Glu at both the variable 2- and 4-positions (Figure 1). The abundance of [d-Asp^3^]MC-EE was typically ca. 1:55 relative to [d-Asp^3^]MC-ER based on LC–HRMS peak areas, making [d-Asp^3^]MC-EE difficult to detect even in concentrated culture extracts. Product-ions at *m*/*z* 375.1906 and 509.2634 (C_20_H_27_O_5_N_2_^+^, and C_29_H_37_O_6_N_2_^+^, Δ*m* = −1.7 and −2.4 ppm, both originating from Adda^5^–d-Glu^6^–Dha^7^, see Figure 1), and *m*/*z* 446.2278 and 580.3006 (C_23_H_32_N_3_O_6_^+^, and C_32_H_42_N_3_O_7_^+^, Δ*m* = −2.4 and −1.9 ppm, both originating from Adda^5^–d-Glu^6^–Mdha^7^–d-Ala^1^) indicated that **13** contained variations only in positions 2–4. Consistent with this, product-ions at *m*/*z* 262.1026 (C_9_H_16_O_6_N_3_^+^, Δ*m* = −2.1 ppm, from d-Asp^3^–Glu^4^), 391.1451 (C_14_H_23_O_9_N_4_^+^, Δ*m* = −2.2. ppm, from Glu^2^–d-Asp^3^–Glu^4^), and 575.2702 (C_28_H_39_O_9_N_4_^+^, Δ*m* = −1.7 ppm, from Adda^5^–d-Glu^6^–Mdha^7^–d-Ala^1^–Glu^2^) were consistent with Glu at positions-2 and -4 and d-Asp^3^ at position-3 (Figure 6, Figure S28). In addition, examination of the product-ion spectrum of **13** obtained with LC−ITMS/MS method C with published data for [d-Asp^3^]MC-LY (**21**) and [d-Asp^3^]MC-LF (**22**) obtained under similar conditions [31], showed only the mass differences that would be expected from replacing residues-2 and -4 of **21** and **22** with Glu (Table 3). Reaction of **13** with diazomethane gave mainly the trimethyl ester (Figure S38), demonstrating the presence of 3 reactive (d-Glu^6^, Glu^2^, and Glu^4^) and one much less reactive (d-Asp^3^) carboxylic acid groups in **13** and, when taken together with the LC–HRMS/MS data, confirms its identity as [d-Asp^3^]MC-EE (**13**).

[d-Asp^3^]MC-RW (**14**): The LC–HRMS and LC–HRMS/MS data revealed a compound that afforded [M + H]^+^ and [M − H]^−^ ions with *m*/*z* 1054.5387 and 1052.5264 in positive and negative ion modes, respectively (Figure 2, Table 1), and ^15^N-labeling revealed the presence of 11 N atoms, and an elemental composition for the corresponding neutral molecule of C_53_H_71_N_11_O_12_, consistent with a desmethylated congener of MC-RW or MC-WR (Table 1). Product-ions in positive mode at *m*/*z* 375.1904 (C_20_H_27_O_5_N_2_^+^, Δ*m* = −2.8 ppm, from Adda^5^–d-Glu^6^–Mdha^7^ minus C_9_H_10_O, Figure 1) and 426.2084 (C_17_H_28_O_6_N_7_^+^, Δ*m* = −3.0 ppm, from Mdha^7^–d-Ala^1^–Arg^2^–d-Asp^3^) confirmed the site of demethylation as being on position-3 rather than position-7 (d-Asp^3^ rather than Dha^7^) and, together with the complete absence of a product-ion at *m*/*z* 599.3552, confirmed the presence of Arg^2^ rather than Arg^4^ (Figure 6). Comparison with HRMS/MS data for [d-Asp^3^]MC-RY [46] revealed other product-ions at 851.4285 (C_42_H_59_O_11_N_8_^+^, Δ*m* = −1.5 ppm, from Adda^5^–d-Glu^6^–Mdha^7^–d-Ala^1^–Arg^2^–d-Asp^3^) and 302.1125 (C_15_H_16_O_4_N_3_^+^, Δ*m* = −3.4 ppm, from Asp^3^–Trp^4^) consistent with Trp at position-4, and thus that **14** is [d-Asp^3^]MC-RW. Comparison of the observed product-ions from LC–ITMS/MS of the [d-Asp^3^]MC-RW (**14**) (Figure S12) with literature data for the Arg^2^-containing congeners [d-Asp^3^]MC-RY (**17**) and [d-Asp^3^]MC-RF (**18**) (Table 4) [44] was fully consistent with the proposed structure, with **17** and **18** differing only in the amino acid at position-4 (Tyr and Phe, respectively) from [d-Asp^3^]MC-RW (**14**). Product-ions containing these moieties shifted in relation to mass differences between the three amino acids, while other fragments appeared at the same *m*/*z* values for all the compounds (Table 4).

[d-Asp^3^]MC-RY (**17**) and [d-Asp^3^]MC-RF (**18**): These two MCs were minor congeners in the *P. prolifica* NIVA-CYA 544 extract (Figure 2). Their elemental compositions were determined on the basis of the *m*/*z* of their protonated or deprotonated ions, ^15^N-labeling, and analysis of their isotope patterns to be C_51_H_70_N_10_O_13_ and C_51_H_70_N_10_O_12_, respectively (Table 1). [d-Asp^3^]MC-RY (**17**) and [d-Asp^3^]MC-RF (**18**) eluted with the same retention times as **17** and **18** in the extract from L. Victoria, and although the HRMS/MS spectrum of **17** was weak, it displayed several product-ions (*m*/*z* 426.2069, 375.1915, 213.0879, 155.0814, and 135.0801) that were consistent with [d-Asp^3^]MC-RY [46]. The HRMS/MS spectrum of **18** was stronger, and contained product-ions consistent with the proposed structure, including those observed for **17** (above) as well as at *m*/*z* 851.4282 (Δ*m* = −1.9 ppm, from Adda^5^–d-Glu^6^–Mdha^7^–d-Ala^1^–Arg^2^–d-Asp^3^) [46] that were consistent with [d-Asp^3^]MC-RF (**18**).

[d-Asp^3^]MC-RCit (**20**): A relatively early eluting MC congener (retention time 3.44 min using LC–HRMS method A) afforded singly-charged ions at *m*/*z* 1025.5431 and 1023.5311 in positive and negative ionization modes, respectively, corresponding to the [M + H]^+^ and [M − H]^−^ ions, respectively. The number of nitrogen atoms was determined to be 12 using ^15^N-labeling. These data, as well as analysis of the isotope profiles with and without nitrogen-15 labeling (Figure 4) established a molecular formula of C_48_H_72_N_12_O_13_ for the neutral molecule. The retention time of **20** was only slightly shorter than that of **4** in LC−HRMS method B (Figure S6, Table S1), which together with the charge state and molecular formula suggested that **20** contained one Arg residue. The positive HRMS/MS spectrum of **20** (Figure S36) was very similar to those of [d-Asp^3^]MC-RW (**14**) and [d-Asp^3^]MC-RF (**18**) (Figure S37), and in particular displayed prominent product-ions at *m*/*z* 375.1904 (C_20_H_27_O_5_N_2_^+^, Δ*m* = −2.8 ppm, from Adda^5^–d-Glu^6^–Mdha^7^ minus C_9_H_10_O, Figure 1) and 426.2083 (C_17_H_28_O_6_N_7_^+^, Δ*m* = −3.0 ppm, from Mdha^7^–d-Ala^1^–Arg^2^–d-Asp^3^), confirming demethylation at position-3 rather than position-7 (d-Asp^3^ rather than Dha^7^) and, together with the complete absence of a product-ion at *m*/*z* 599.3552, confirmed the presence of Arg^2^ rather than Arg^4^. Furthermore, product-ions at 851.4314 (C_42_H_59_O_11_N_8_^+^, Δ*m* = +1.9 ppm, from Adda^5^–d-Glu^6^–Mdha^7^–d-Ala^1^–Arg^2^–d-Asp^3^) and 273.1193 (C_10_H_17_O_5_N_4_^+^, Δ −0.2 ppm, from d-Asp^3^–Cit^4^) were consistent with Cit at position-4, and thus that **20** is [d-Asp^3^]MC-RCit. In particular, the LC–HRMS/MS data indicated that the side-chain of the amino acid at position-2 was neutral, and consisted of a C_4_H_9_ON_2_ unit that included exactly one ring or double bond. Given that the two nitrogen atoms cannot be basic (due to the molecule’s charge state, retention time, and fragmentation pattern), both of the nitrogen atoms must be either side of a carbonyl group, indicating the presence of a carbamide group R-NH-CONH_2_. This is consistent with Cit, which is by far the most likely of the possibilities based on biosynthetic and metabolic considerations. Furthermore, **20** showed a prominent product at *m*/*z* 982.5310 (Figures S36 and S64), which examination of the product-ion spectra from unlabeled and ^15^N-labeled **20** (Figure S64) unambiguously showed to be due to neutral loss of HNCO. This neutral loss is a characteristic of Cit-containing peptides [47] and, together with the foregoing observations, establishes **20** as [Asp^3^]MC-RCit. This is the first Cit-containing MC to be identified. Given that Cit is involved in both the biosynthesis and catabolism of Arg in bacteria [48], **20** may be a minor byproduct from biosynthesis of the much more abundant **1** or originate from the subsequent breakdown of **1** in the cells.

A number of minor MCs were detected by LC–HRMS method B in extracts of NIVA-CYA 544 (Figures S5–S8) but were not sufficiently abundant to be identified with any certainty from the LC–MS data. One of these is believed to be [d-Asp^3^]MC-RY(OMe) based on its accurate mass (*t*_R_ = 8.79 min, *m*/*z* 1061.5310, C_52_H_73_N_10_O_14_^+^ Δ*m* = +0.7 ppm) and relative retention time (eluting in the tail of the much more abundant [d-Asp^3^]MC-RY (**17**) (Figure S8), as has been observed elsewhere [44]). The limited number of product-ions observed (Figure S34) were also fully consistent with **17**. Another appeared to be a major and a minor isomer of the Cys conjugate of **1** (Figure S5, major isomer *t*_R_ = 3.74 min, *m*/*z* 573.2922, C_51_H_82_N_14_O_14_S^2+^, Δ*m* = −1.5 ppm, containing 14 nitrogen atoms by ^15^N-labeling and with an excellent isotope pattern match to the proposed structure (Figures S55–S57), the presence of which is unsurprising given the identification of the corresponding GSH conjugate (**19**) in the same extract. An oxidized analog of **1**, containing one extra oxygen atom (Figure S5, *t*_R_ = 4.09 min, *m*/*z* 520.7796, C_48_H_75_N_13_O_13_^2+^, Δ*m* = −0.4 ppm, containing 13 nitrogen atoms by ^15^N-labeling and with an excellent isotope pattern match for the proposed structure) was detected, but the location of the additional oxygen atom was not determined.

In summary, multiple LC–MS analyses were applied for the tentative identification of new MC congeners in *P. prolifica* NIVA-CYA 544. We showed the application of different modes of mass spectrometric fragmentation in order to obtain complementary structural information. Further structural elucidation was aided by specific derivatization techniques of functional groups and ^15^N-labeling of the peptides, as well as analysis of the isotope patterns observed for the compounds during LC–HRMS analysis of unlabeled and ^15^N-labeled culture extracts. This resulted in the characterization of new glutamic acid- (**12** and **13**) and citrulline-containing (**20**) microcystins as well as a tryptophan-containing analog (**14**). The identity of the high molecular weight MC-containing **15** has tentatively been shown to be a sulfide-linked conjugate of [d-Asp^3^]MC-RR (**1**), with its sulfoxide derivative **16** present as an autoxidation product in the extracts, but further studies are needed for definitive structural determination of **15** and **16**. Nevertheless, the detection of these unusual compounds illustrates the power of the combined chemical and LC–MS analytical methods used in this study. Furthermore, the presence of **15** and **16** in this culture suggests that similar high-molecular-mass MC conjugates may be produced by other cyanobacterial cultures and blooms, but would be difficult to detect by standard methods due to the combination of their unusual mass, charge-state and retention times. If similar conjugates exist for non-Arg-containing MCs, they are expected to elute very late and be singly-charged in positive ionization mode with *m*/*z* > 2000.

MCs containing Glu at positions-2 and -4 have been reported [45] as methyl esters at one or both positions. We did not observe any methyl esters of MCs in the NIVA-CYA 544 extracts in this study. Furthermore, during the chemical characterization of the MCs in this strain, we found that the carboxylic acid groups on Glu^2^, Glu^4^, d-Glu^6^ were readily esterified by diazomethane. The above findings for NIVA-CYA 544, taken together with the observation that d-Glu^6^ in MCs is known to be readily esterified by methanol in the presence of traces of acid [25,26], suggests that the originally-reported esterified MC-EE congeners [45] in *Anabaena* strain 186 may have been artifacts from reaction of the carboxylic acid groups of Glu^2^ and Glu^4^ with solvent during extraction and purification in a similar manner to that which has been described for d-Glu^6^. If so, then the seven MCs in the *Anabaena* strain 186 identified by Namikoshi et al. [45] would originally have been biosynthesized as [Dha^7^]MC-EE, [d-Asp^3^,Dha^7^]MC-EE, [Ser^7^]MC-EE, [d-Asp^3^,Ser^7^]MC-EE and MC-EE in the cyanobacterium. It would appear from this that the carboxylic acid groups in the Glu residues at position-2 and -4 of MC-EE congeners might be even more easily esterified than the carboxylic acid of d-Glu^6^, as none of the esterified MC-EE congeners reported by Namikoshi et al. contained a d-Glu(OMe)^6^ residue. The potential presence of artefactual MCs containing methyl esters after exposure to methanol and acids needs to be considered when using LC–MS methods to analyze processed extracts from cyanobacterial blooms or cultures. A Cit-containing MC, [d-Asp^3^]MC-RCit (**20**), was also detected for the first time. It seems likely that this compound is related to the presence of the much more abundant [d-Asp^3^]MC-RR (**1**), and therefore that low levels of Cit-containing MCs could be present in other cyanobacterial samples with high levels of Arg-containing MCs. The above findings also illustrate the power of combining LC–HRMS/MS techniques with isotopic labeling and selective chemical derivatization techniques and highlight the unexpected MC diversity that may be present in cyanobacteria and which could be easily overlooked using more conventional analytical approaches.

## 3. Experimental Section

### 3.1. Chemicals and Reagents

LC–MS grade water and acetonitrile were from Fisher Scientific (Oslo, Norway). Methanol (gradient quality) was from Romil (Cambridge, UK). The following MC standards (≥95% purity) were from Enzo Life Sciences (Enzo Biochem, Inc., Farmingdale, NY, USA): Hepatotox Set 1 (containing MC-LR (**6**), MC-RR (**2**), MC-LY (**8**), MC-YR (**3**), MC-LW (**9**), MC-LF (**10**), MC-LA, and NOD-R), [d-Asp^3^,Dhb^7^]MC-RR (**23**) (supplied as [d-Asp^3^]MC-RR (**1**), but subsequently identified as **23** by thiol derivatization and comparison with reference materials of **1** and **23** using LC-MS/MS methods A and B), and [d-Asp^3^]MC-LR (**4**). A certified reference material of [Dha^7^]MC-LR (**5**) and reference materials of [d-Asp^3^]MC-RR (**1**) and [d-Asp^3^,Dhb^7^]MC-RR (**23**) were from National Research Council of Canada (NRC, Halifax, NS, Canada). Extracts of a bloom from Lake Victoria that contained [d-Asp^3^]MC-RY (**17**), [d-Asp^3^]MC-RF (**18**) and [d-Asp^3^]MC-LA were available from earlier work [43]. Individual stock solutions of 12.5 µg/mL (MC-LY, MC-LW, MC-LF, MC-LA, [d-Asp^3^,Dhb^7^]MC-RR, [d-Asp^3^]MC-LR, 25 µg/mL (MC-RR), and 50 µg/mL (MC-LR, NOD-R), were prepared in 50% methanol. From those solutions, a pooled working stock of 1 µg/mL (each compound), was prepared in methanol and diluted to 200 ng/mL in 50% methanol. Sodium carbonate (pro analysis), 2-mercaptoethanol (≥99%), diazald (99%), l-glutathione (≥98%) and 2-(2-ethoxyethoxy)ethanol (≥99%) were from Sigma–Aldrich (Steinheim, Germany). Sodium bicarbonate, potassium hydroxide, formic acid and acetic acid (all pro analysis purity) were from Merck KGaA (Darmstadt, Germany). Diaion HP-20 resin was from Supelco Analytical (Bellefonte, PA, USA).

### 3.2. Cultivation of P. prolifica NIVA-CYA 544 and Extraction of MCs

*P. prolifica* strain NIVA-CYA 544 was from The Norwegian Culture Collection of Algae (NORCCA) maintained and owned by the Norwegian Institute for Water Research (NIVA) and the University of Oslo. The strain was originally isolated from Lake Steinsfjorden, Norway, in 2004. It was cultivated in Z8 medium [49] in 100 mL glass Erlenmeyer flasks in an incubator (IPP110plus, Memmert GmbH + Co. KG, Schwabach, Germany) at 18 °C with a 14/10 h light/dark photoperiod, using 1% of maximum light intensity. For general screening of MCs, 3 mL of the culture was transferred to a glass tube and stored at −20 °C overnight, then allowed to thaw at room temperature, and 3 mL of methanol was added. The tube was then vortex-mixed for 20 s, sonicated for 5 min and centrifuged for 10 min at 1000 rcf. The supernatant was transferred to screw-cap vials and stored refrigerated until analysis. A concentrated extract to assist in MS/MS analyses of the minor congeners was obtained with HP-20 as described elsewhere [33].

### 3.3. Cultivation of P. prolifica NIVA-CYA 544 for ^15^N-labeling of MCs

In late exponential phase, a culture (15 mL) was concentrated by centrifugation (8000 rcf, swinging bucket rotor, 4 °C, 15 min), and the supernatant removed. Concentrated cells (~3 mL) were inoculated into 17 mL of sterile Z8 medium in which the NaNO_3_ and Ca(NO_3_)_2_ had been replaced with Na^15^NO_3_ and Ca(^15^NO_3_)_2_ (>98% ^15^N, Cambridge Isotope Laboratories, Andover, MA, USA). Cultures were grown at 18 °C under a 14:10 h light/dark photoperiod in a Conviron model E7/2 dual compartment plant growth chamber. An approximate photon flux density of 95–100 µmol m^−2^ s^−1^ cool white light was maintained. The light was measured outside the flask using a Li-Cor Model LI-185B quantum/photometer. Cultures were transferred every 3 weeks at which time small aliquots were examined by LC–HRMS analysis (method B) until maximum ^15^N-incorporation (~98%) was observed (~12 weeks and 4 transfers) based on isotopic composition of **1** and **4**. After 20 transfers (~13 months), a concentrated extract was obtained from 15 mL of the labeled and unlabeled cultures for full-scan LC–HRMS (method B) analysis using HP-20 as described elsewhere [33], and the data used for analysis of the isotopic composition of the MCs.

### 3.4. Liquid Chromatography–Mass Spectrometry

#### 3.4.1. LC–HRMS and LC–HRMS/MS (Method A)

HPLC was performed using a Kinetex F5 column (150 × 2.1 mm, 2.6 μm, Phenomenex, Torrance, CA, USA) at 30 °C. The flow rate was 0.3 mL/min, and the injection volumes were 5–10 μL. Mobile phase A was 0.1% formic acid in the water, and mobile phase B was 0.1% formic acid in acetonitrile. The separation was performed by isocratic elution using 30% B for 0.5 min, followed by a linear gradient to 50% B over 14.5 min. The column was flushed with 100% B for 2 min before returning to the starting conditions and equilibration for 2 min. A Vanquish Horizon UHPLC (Thermo Fisher Scientific, Waltham, MA, USA) was interfaced with a Q-Exactive Fourier-transform high-resolution mass spectrometer (Thermo Fisher Scientific). A heated electrospray interface (HESI-II) was operated at 300 °C and used for ionization with a spray voltage of 3.8 kV and 3.5 kV in positive and/or negative mode, respectively. The mass spectrometer was run in the positive or negative full-scan mode in the mass range *m*/*z* 400−2200. The mass resolution was set to 70,000 at *m*/*z* 200. Other important interface parameters included an ion transfer capillary temperature of 250 °C, a sheath gas flow rate of 55 units, and an auxiliary gas flow rate of 25 units. All-ion-fragmentation (AIF) was performed using a mass resolution of 17,500, a max IT of 200 ms, and an AGC target of 3 × 10^6^. The normalized collision energy was set to 35%. The mass range during AIF was *m*/*z* 80−1200. Parallel reaction monitoring (PRM) was performed using a mass resolution of 17,500.

#### 3.4.2. LC–HRMS and LC–HRMS/MS (Method B)

LC-HRMS method B used a Q Exactive-HF Orbitrap mass spectrometer equipped with a HESI-II heated electrospray ionization interface (ThermoFisher Scientific, Waltham, MA, USA) using an Agilent 1200 LC system including a binary pump, autosampler and column oven (Agilent, Santa Clara, CA, USA). Analyses were performed with a SymmetryShield C18 column (100 × 2.1 mm, 3.5 µm, Waters, Milford, MA, USA) held at 40 °C with mobile phases A and B of water and acetonitrile, respectively, each of which contained formic acid (0.1% v/v). Gradient elution (0.3 mL/min) was from 20–90% B over 18 min, then to 100% B over 0.1 min and a hold at 100% B (2.9 min), then returned to 20% B over 0.1 min with a hold at 20% B (3.9 min) to equilibrate the column (total run time 25 min). Injection volume was typically 1–5 µL.

The mass spectrometer was operated in positive ion mode and calibrated from *m*/*z* 74–1622. The spray voltage was 3.7 kV, the capillary temperature was 350 °C, and the sheath and auxiliary gas flow rates were 25 and 8 units, respectively, with MS data acquired from 2–20 min. Mass spectral data was collected using a combined full-scan and data-independent acquisition (DIA) method. Full-scan data was collected over a range from *m*/*z* 500–1400 using the 60,000-mass resolution setting, an AGC target of 1 × 10^6^ and a max IT of 100 ms. DIA data was collected using the 15,000-mass resolution setting, an AGC target of 2 × 10^5^, max IT set to ‘auto’ and a stepped collision energy of 30, 60 and 80 eV. Precursor isolation windows were 62 *m*/*z* wide centered at *m*/*z* 530, 590, 650, 710, 770, 830, 890, 950, 1010, 1070, 1130, 1190, 1250, 1310, and 1370, and DIA chromatograms were typically extracted for the following product-ions: *m*/*z* 121.1011, 121.0647, 135.0804, 135.1168, 213.0870, 361.1758, 375.1915, 379.1864, 389.2072, 393.2020, 412.1939, 426.2096, 440.2252, 454.2409, 585.3395, 599.3552, 613.3709. Putative MCs detected using the above full-scan/DIA method were further probed in a targeted manner using the PRM mode with a 0.7 *m*/*z* precursor isolation window, typically using the 30,000-resolution setting, an AGC target of 5 × 10^5^ and a max IT of 400 ms. Typical collision energies were: stepped CE at 30 and 35 eV for MCs with no Arg, stepped CE at 60, 65 and 70 eV for MCs with one Arg, and CE at 65 eV for [M + H]^+^ and stepped CE at 20, 25 and 30 eV for [M + 2H]^2+^ of MCs with two Arg groups. Full-scan chromatograms were obtained in MS-SIM mode as above but with mass resolution 120,000 and max IT 300 ms.

In negative mode, the mass spectrometer was calibrated from *m*/*z* 69–1780 and the spray voltage was −3.7 kV, while the capillary temperature, sheath, and auxiliary gas flow rates were the same as for positive mode. Mass spectrometry data were collected in full-scan/DIA scan mode as above using a scan range of *m*/*z* 750–1400, a mass resolution setting of 60,000, AGC target of 1 × 10^6^ and max IT of 100 ms. For DIA, HRMS/MS data were collected from *m*/*z* 93–1400 using a resolution setting of 15,000, AGC target of 2 × 10^5^, max IT set to ‘auto’, and stepped collision energy 65 and 100 V. Isolation windows were 45 *m*/*z* wide and centered at *m*/*z* 772, 815, 858, 902, 945, 988, 1032, 1075, 1118, 1162, 1205, 1248, 1294, 1335, and 1378, and DIA chromatograms were extracted for the *m*/*z* 128.0353 (or *m*/*z* 129.0324 for ^15^N-labeled MCs) product-ion. Full-scan chromatograms were obtained over a scan range *m*/*z* 750–1400 at a mass resolution setting of 120,000 using an AGC target of 1 × 10^6^ and a max IT of 300 ms.

#### 3.4.3. LC–ITMS/MS (Method C)

The HPLC conditions were identical as for LC–HRMS method A. However, a Finnigan Surveyor HPLC system was interfaced with an LTQ linear ion trap mass spectrometer (both Thermo Fisher Scientific) operated in positive or negative ionization mode and fitted with an electrospray ionization interface. The capillary voltage and tube lens offset of the instrument were tuned with continuous infusion of MC-LR (10 μg/mL) in 50% methanol into a mobile phase composed of 50% A. The spray voltage was set to 3.5 kV, the sheath gas and auxiliary gas flow rates were 58 and 3.0 units, respectively, and the capillary temperature was 275 °C. The MS/MS product-ion spectra of the [M + H]^+^ and [M − H]^−^ ions were acquired using collision-induced dissociation in the ion trap. The ESI settings were as described above. Individual precursor ions were selected with an isolation width of *m*/*z* 2, the activation Q was set to 0.25, and the activation time was set to 30 ms. The normalized collision energy was 35%.

### 3.5. 2-Mercaptoethanol Derivatization for Mdha^7^/Dhb^7^ Differentiation

To an aliquot (100 µL) of the 50% methanol extract of *P. prolifica* was added 16 µL of 5 µg/mL MC-LR as internal standard (in 50% methanol), and then mixed with 60 µL of 0.2 M sodium carbonate buffer (pH = 9.2) in a septum-capped vial, left in the autosampler tray [28,29] at 20 °C for 20 min, and then analyzed by LC–HRMS (method A). Then, 2-mercaptoethanol (1 µL) was added, with brief vortex-mixing, and the vial placed back in the autosampler tray. The reaction was then followed by LC–HRMS method A for 3 h.

Separate derivatization experiments were performed by the addition of ammonium carbonate (0.1 M, 200 µL) to a filtered extract (200 µL), with 200 µL transferred to two LC-MS vials. To one vial was added 1 µL of a 1:1 mixture of mercaptoethanol and *d*_4_-mercaptoethanol (Sigma–Aldrich, St. Louis, MO, USA), while 1 µL of water was added to the other vial as a control. The samples were placed in the LC sample tray (15 °C) and the reactions monitored periodically until completion and then analyzed using LC–HRMS method B.

### 3.6. Methylation of Carboxylic Acids

An aliquot of the cyanobacterial extract (200 µL) was transferred to the outer tube of a diazomethane-generator (Aldrich, Steinheim, Germany) and 3 mL of methanol added. The extract was then exposed to diazomethane generated from diazald (*N*-methyl-*N*-nitroso-*p*-toluenesulfonamide) in the apparatus, according to the manufacturer’s instructions. After 18 h, 1 mL acetic acid was added to the inner tube to remove unreacted diazomethane, the methanol solution was transferred to a glass tube and evaporated at 60 °C under a gentle stream of nitrogen. The residue was dissolved in 100 µL of methanol, vortex-mixed, and transferred to an LC vial for LC–HRMS analysis using LC–HRMS method A.

### 3.7. ^15^N-Incorporation and Molecular Formula Calculations

The incorporation of ^15^N into the microcystin with an established molecular formula (**1**) in cultures of *P. prolifica* NIVA-CYA 544 grown in ^15^N-labeled media was calculated with NRC Isotopic Enrichment Calculator (https://metrology.shinyapps.io/isotopic-enrichment-calculator/, v.1.81) using intensities of the peaks in the isotope envelopes obtained with LC-HRMS method B (Figures S40 and S41). For MCs where the molecular formula was not established, the number of nitrogen atoms present was determined from the separation of the isotope envelope peaks of the labeled and unlabeled compound, greatly restricting the number of feasible molecular formulae that were consistent with the accurate mass of the compound. A second program, NRC Molecular Formula Calculator (https://metrology.shinyapps.io/molecular-formula-calculator/, v.1.01), the features of which are described in the text, was used to obtain the most likely molecular formula for unknown MCs, based on the accurate masses and relative intensities of the isotope envelope peaks of the labeled and unlabeled MC obtained from a mixture of labeled and unlabeled extracts, and on the isotopic composition of ^15^N in MCs as established for labeled **1**. Candidate patterns were ranked using Bayesian statistics as implemented in R package Rdisop [39,40] and except for **15** and **16** (where several viable formulae were obtained), the match with the highest score was chosen (scores were normalized to the best match).

### 3.8. Reaction of ***1*** with Glutathione to Produce ***19***.

Derivatization of **1** with GSH was based on Foss et al. [25] and proceeded by the addition of 200 μL of GSH (2.5 mg/mL in pH 9.4 carbonate buffer) to ca. 40 ng [d-Asp^3^]MC-RR (**1**) in methanol (90 μL). The progress of the reaction was followed by LC-HRMS method A (Figure S15).

### 3.9. Oxidation with Sodium Periodate

Periodate oxidations, based on Yilmaz et al. [33], were performed by the addition of aqueous sodium periodate (1 mg/mL) to an equal volume (100 μL) of the filtered extract. Samples and reactions were placed in the sample tray (held at 15 °C) and the reactions monitored periodically until completion and then analyzed.

## Figures and Tables

**Table 1:** Properties of MCs identified in *P. prolifica* NIVA-CYA 544 (LC–HRMS method A unless specified).

	Microcystin	Confidence	Neutral Formula *^a^*	No. N *^b^*	*t*_R_ (min) *^c^*	Positive *^d^ m/z*	+*z*	Δ*m* (ppm)	Thiol-Reactive	No. CO_2_H *^e^*
**1**	[d-Asp^3^]MC-RR	confirmed	C_48_H_73_N_13_O_12_	13	2.81	512.7815	2	−1.6	yes	2
**4**	[d-Asp^3^]MC-LR	confirmed	C_48_H_72_N_10_O_12_	10	5.72	981.5419	1	+1.6	yes	2
**11**	[d-Asp^3^,Mser^7^]MC-RR	probable	C_48_H_75_N_13_O_13_	13	2.59	521.7879	2	+0.5	no	ND
**12**	[d-Asp^3^]MC-ER	probable	C_47_H_68_N_10_O_14_	10	3.63	997.4988	1	−0.2	yes	3
**13**	[d-Asp^3^]MC-EE	probable	C_46_H_63_N_7_O_16_	7	4.99	970.4413	1	+0.9	yes	4
**14**	[d-Asp^3^]MC-RW	probable	C_53_H_71_N_11_O_12_	11	10.01	1054.5387	1	+2.9	yes	ND
**15**	Sulfide conjugate of **1**	tentative	C_93_H_145_N_21_O_33_S	21	9.85 *^f^*	1059.0076	2	−1.3	no	ND
**16**	**15**-sulfoxide	tentative	C_93_H_145_N_21_O_34_S	21	6.50 *^g^*	1067.0052	2	−1.2	no	ND
**17**	[d-Asp^3^]MC-RY	probable	C_51_H_70_N_10_O_13_	10	7.16	1031.5204	1	+0.7	yes	ND
**18**	[d-Asp^3^]MC-RF	probable	C_51_H_70_N_10_O_12_	10	9.94	1015.5246	1	−0.2	yes	ND
**19**	GSH-conjugate of **1**	confirmed	C_58_H_90_N_16_O_18_S	16	2.05	666.3251	2	+1.3	no	ND
**20**	[d-Asp^3^]MC-RCit	probable	C_48_H_72_N_12_O_13_	12	3.44	1025.5431	1	+1.6	yes	ND

*^a^* Except for **15** and **16**, only one single viable formula was obtained for each compound via analysis of its isotope patterns obtained from LC–HRMS (method B) in both negative and positive ionization modes using the National Research Council of Canada (NRC) Molecular Formula Calculator. See Figures S40–S57. *^b^* Number of nitrogen atoms, as measured from ^15^N-labeled culture (LC–HRMS method B). *^c^* Retention times of standards (min): [d-Asp^3^]MC-RR (**1**), 2.82; MC-RR (**2**), 3.12; MC-YR (**3**), 5.22; [d-Asp^3^]MC-LR (**4**), 5.71; [Dha^7^]MC-LR (**5**), 4.67 (method C); MC-LR (**6**), 5.84; MC-LA (**7**), 9.35; MC-LY (**8**), 10.65; MC-LW (**9**), 13.83; MC-LF (**10**), 13.91; [d-Asp^3^,Dhb^7^]MC-RR (**23**), 2.97 (Table S1). *^d^* Measured *m*/*z* for [M + H]^+^ or [M + 2H]^2+^. *^e^* Measured by esterification with CH_2_N_2_. ND = not determined. *^f^* Minor isomer also present at 9.46 min. *^g^* Minor isomer also present at 5.85 min.

**Table 2:** Assignments of observed product-ions and their *m*/*z* from collision-induced dissociation of [M + H]^+^ of [d-Asp^3^]MC-ER (**12**) obtained using LC–ITMS/MS (method C) and comparison to the corresponding product-ions observed for MC-LR (**6**), [Dha^7^]MC-LR (**5**) and [d-Asp^3^]MC-LR (**4**).*^a^*

Fragment Ion Assignment	(6)	(5)	(4)	(12)
[M + H]^+^	995.6	981.5	981.5	997.5
[M − NH_3_+ H]^+^	978.6	964.5	964.5	980.5
[M − H_2_O +H]^+^	977.6	963.6	963.5	979.5
[M − CO + H]^+^	967.6	953.6	953.6	969.5
[M − 48 + H]^+^	946.5	932.5	932.5	948.4
[Arg-Adda-Glu-res^7^-Ala-X^2^-NH_2_ + 2H]^+^	883.6	869.6	883.5	899.5
[Arg-Adda-Glu-res^7^-Ala-X^2^ + H]^+^	866.6	852.5	866.6	882.5
[res^7^-Ala-X^2^-res^3^-Arg-Adda + H]^+^	866.6	852.5	852.5	868.5
[M−Addafrag. + H]^+^	861.5	847.5	847.5	863.4
[M−Addafrag. − NH_3_ + H]^+^	844.5	830.4	830.4	846.4
[Arg-Adda-Glu-res^7^-Ala- X^2^-CO + H]^+^	838.6	824.6	838.6	854.5
[Ala-X^2^-res^3^-Arg-Adda − Addafrag. + H]^+^	783.5	783.5	769.6	785.4
[Arg-Adda-Glu-res^7^-Ala + H]^+^	753.5	739.5	753.5	753.5
[res^3^-Arg-Adda-Glu + H]^+^	728.5	728.5	714.4	714.4
[res^3^-Arg-Adda-Glu − H_2_O + H]^+^	710.4	710.4	696.4	696.4
[Arg-Adda-Glu-res^7^ + H]^+^	682.4	668.4	682.4	696.4
[Glu-res^7^-Ala-X^2^-res^3^-Arg + H]^+^	682.4	668.4	668.4	684.4
[Arg-Adda-Glu + H]^+^	599.4	599.4	599.4	599.4
[Arg-Adda-Glu − NH_3_ + H]^+^	582.4	582.4	582.4	582.4
[Arg-Adda-Glu − CO + H]^+^	571.4	571.4	571.4	571.4
[res^7^-Ala-X^2^-res^3^-Arg-NH_2_ + 2H]^+^	570.4	556.4	556.4	572.3
[res^7^-Ala-X^2^-res^3^-Arg + H]^+^	553.4	539.4	539.4	555.3
[Ala-X^2^-res^3^-Arg + H]^+^	470.4	470.4	456.4	472.3
[Ala-X^2^-res^3^-Arg − NH_3_ + H]^+^	453.3	453.3	439.3	455.3
[Adda-Glu-res^7^ − Addafrag. − NH_3_ + H]^+^	375.3	361.2	375.3	375.3
[res^3^-Arg-NH_2_ + H]^+^	303.2	303.2	289.2	289.2
[res^3^-Arg + H]^+^	285.2	285.2	ND	ND

*^a^* ND = Not Detected; X^2^ = Leu (**6**, **5**, **4**) or Glu (**12**); res^3^ = Masp (**6**, **5**) or Asp (**4**, **12**); res^7^ = Mdha (**6**, **4**, **12**) or Dha (**5**); Addafrag = C_9_H_10_O.

**Table 3:** Assignments of observed product-ions and their *m*/*z* from collision-induced dissociation of [M + H]^+^ of [d-Asp^3^]MC-EE (**13**) and comparison to the corresponding product-ions from [d-Asp^3^]MC-LY (**21**) and [d-Asp^3^]MC-LF (**22**). LC–ITMS/MS method C was used for acquisition of the data for **13**, data for **21** and **22** are from Miles et al. [44].*^a^*

Fragment Ion Assignment	(21)	(22)	(13)
[M + H]^+^	988	972	970.4
[M − NH_3_ + H]^+^	971	955	953.4
[M − H_2_O + H]^+^	970	954	952.5
[M − CO + H]^+^	960	944	942.6
[M − Addafrag + H]^+^	854	838	836.4
[M − Addafrag − NH_3_ + H]^+^	837	821	819.4
[M − Addafrag − H_2_O + H]^+^	836	820	818.5
[Adda-Glu-Mdha-Ala-X^2^ − NH_3_+ H]^+^	693	693	709.3
[M − Adda + H]^+^	675	659	657.3
[M − Adda − H_2_O + H]^+^	ND	ND	639.2
[Adda-Glu-Mdha-Ala − NH_3_ + H]^+^	580	580	580.3
[Adda-Glu-Mdha-Ala-X^2^ − Addafrag − NH_3_+ H]^+^	559	559	575.2
[Z^4^-Asp-X^2^-Ala-Mdha-NH_2_ + 2H]^+^	563	547	545.4
[Z^4^-Asp-X^2^-Ala-Mdha + H]^+^	546	530	528.3
[Adda-Glu-Mdha − NH_3_ + H]^+^	509	509	509.3
[Z^4^-Asp-X^2^-Ala-NH_2_ + 2H]^+^	480	464	462.3
[Z^4^-Asp-X^2^-Ala + H]^+^	463	447	445.2
[Z^4^-Asp-X^2^ + H]^+^	392	376	374.3
[Adda-Glu-Mdha − Addafrag − NH_3_ + H]^+^	375	375	375.3

*^a^* ND = Not Detected; X^2^ = Leu (**21**, **22**) or Glu (**13**); Z^4^ = Tyr (**21**), Phe (**22**) or Glu (**13**); Addafrag = C_9_H_10_O.

**Table 4:** Assignments of observed product-ions and their *m*/*z* from collision-induced dissociation of [M + H]^+^ of [d-Asp^3^]MC-RW (**14**) and comparison to the corresponding product-ions from [d-Asp^3^]MC-RY (**17**) and [d-Asp^3^]MC-RF (**18**). LC–MS method C was used for acquisition of the data for **14**, data for **17** and **18** are from Miles et al. [44].*^a^*

Fragment Ion Assignment	17	18	14
[M + H]^+^	1031	1015	1054.8
[M − NH_3_ + H]^+^	1014	998	1037.7
[M − H_2_O + H]^+^	1013	997	1036.7
[M − CO + H]^+^	1003	987	1026.7
[Z^4^-Adda-Glu-Mdha-Ala-Arg − NH_3_ + H]^+^	916	900	939.6
[M − Addafrag + H]^+^	897	881	920.7
[Adda-Glu-Mdha-Ala-Arg-Asp − NH_3_ + H]^+^	851	851	851.6
[Ala-Arg-Asp-Z^4^-Adda + H]^+^	819	803	842.5
[Adda-Glu-Mdha-Ala-Arg + H]^+^	754	754	754.6
[Adda-Glu-Mdha-Ala-Arg − H_2_O + H]^+^	736	736	736.5
[Adda-Glu-Mdha-Ala-Arg-Asp − Addafrag − NH_3_ + H]^+^	717	717	717.5
[Mdha-Ala-Arg-Asp-Z^4^-NH_2_ + H]^+^	606	590	629.5
[Mdha-Ala-Arg-Asp-Z^4^-NH_2_ − H_2_O + H]^+^	588	572	611.5
[Mdha-Ala-Arg-Asp-Z^4^ − NH_3_ + H]^+^	572	556	595.3
[Glu-Mdha-Ala-Arg-Asp + H]^+^	555	ND	555.4
[Arg-Asp-Z^4^ + H]^+^	435	419	458.4
[Mdha-Ala-Arg-Asp + H]^+^	426	426	426.4
[Mdha-Ala-Arg-Asp − NH_3_ + H]^+^	409	409	409.3
[Glu-Mdha-Ala-Arg − CO_2_H + H]^+^	395	395	395.3
[Adda-Glu-Mdha − Addafrag − NH_3_ + H]^+^	375	375	375.3
[Mdha-Ala-Arg + H]^+^	311	311	311.2

*^a^* ND = Not Detected; Z^4^= Tyr (**17**), Phe (**18**), or Trp (**14**); Addafrag = C_9_H_10_O.

## Supplementary Materials

The following are available online at https://www.mdpi.com/1660-3397/17/11/643/s1, LC–MS chromatograms, MS and MS/MS spectra of 15N-labeled, unlabeled and derivatized samples (Figures S1–S41, S55, S56, and S58–S67); table of retention times for LC–MS methods A–C (Table S1); analysis of mass spectral data with the NRC Molecular Formula Calculator (Figures S42–S54 and S57); computer code for the isotope enrichment and molecular formula calculators are available publicly at https://github.com/meijaj/molecular-formula-calculator and https://github.com/meijaj/isotopic-enrichment-calculator.
