# Supplementary material for: Novel Microcystins from Planktothrix prolifica NIVA-CYA 544 Identified by LC-MS/MS, Functional Group Derivatization and 15N-labeling
Source: Mar Drugs. 2019 Nov 15;17(11):643. doi: 10.3390/md17110643 (PMC6891653; doi:10.3390/md17110643)
Supplement: Supplementary file 1 [file marinedrugs-17-00643-s001.pdf]

Supplementary Material

Novel microcystins from *Planktothrix prolifica* NIVA-CYA 544 identified by LC-MS/MS, functional group derivatization and ^{15}N-labeling

Vittoria Mallia,[1,2] Silvio Uhlig,[1] Cheryl Rafuse,[3] Juris Meija,[4] and Christopher O. Miles[3]

[1]*Toxinology Research Group, Norwegian Veterinary Institute, Ullevålsveien 68, N-0454 Oslo, Norway*

[2]*Department of Chemistry, University of Oslo, P.O. Box 1033, N-0315 Oslo, Norway*

[3]*National Research Council, 1411 Oxford Street, Halifax, NS, B3H 3Z1, Canada*

[4]*National Research Council, 1200 Montreal Road, Ottawa, ON, K1A 0R6, Canada*

Table of contents

**Table S1**	Retention times of microcystins of interest using the three LC–MS methods	S3
**Figure S1**	LC–HRMS chromatogram of the 10 standards used for optimization	S4
**Figure S2**	LC–HRMS chromatograms and mass spectra: oxidation of **15** to **16**	S5
**Figure S3**	LC–HRMS chromatograms: oxidation of **15** to **16**	S6
**Figure S4**	LC–HRMS/MS and –MS/MS product ion spectra of [D-Asp^3]MC-LR (**4**), -ER (**12**)	S7
**Figure S5**	LC–HRMS chromatograms of an HP-20 extract of NIVA-CYA 544 (2.0–4.5 min)	S8
**Figure S6**	LC–HRMS chromatograms of an HP-20 extract of NIVA-CYA 544 (5.5–7.5 min)	S9
**Figure S7**	LC–HRMS chromatograms of an HP-20 extract of NIVA-CYA 544 (5.8–7.8 min)	S10
**Figure S8**	LC–HRMS chromatograms of an HP-20 extract of NIVA-CYA 544 (8.5–12 min)	S11
**Figure S9**	LC−MS/MS DIA chromatograms of an extract of NIVA-CYA 544 (^{14}N and ^{15}N)	S12
**Figure S10**	Positive LC−MS/MS DIA chromatograms of an extract of NIVA-CYA 544	S13
**Figure S11**	Negative LC−MS/MS DIA chromatograms of an extract of NIVA-CYA 544	S14
**Figure S12**	LC–MS/MS product-ion spectra of [D-Asp^3]MC-ER (**12**), -EE (**13**), -RW(**14**)	S15
**Figure S13**	LC–MS/MS positive product-ion spectra of **4**, **5**, **6**, **12**	S16
**Figure S14**	LC–MS/MS negative product-ion spectra of **4**, **5**, **6**, **12**	S17
**Figure S15**	LC–HRMS chromatograms: glutathione reaction (converting **1** to **19**)	S18
**Figure S16**	LC–HRMS/MS PRM spectra of [D-Asp^3]MC-RR (**1**), (^{14}N and ^{15}N)	S19
**Figure S17**	LC–HRMS/MS PRM spectra of [D-Asp^3]MC-RR (**1**), ($[M+2H]^{2+}$ and $[M+H]^+$)	S20
**Figure S18**	LC–HRMS/MS PRM spectra of [D-Asp^3]MC-LR (**4**), (^{14}N and ^{15}N)	S21
**Figure S19**	LC–HRMS/MS PRM spectra of [D-Asp^3,Mser^7]MC-RR (**11**), (^{14}N and ^{15}N)	S22
**Figure S20**	LC–HRMS/MS PRM spectra of [D-Asp^3]MC-ER (**12**), (^{14}N and ^{15}N)	S23
**Figure S21**	LC–HRMS/MS PRM spectra of [D-Asp^3]MC-LR (**4**), -ER (**12**)	S24
**Figure S22**	LC–HRMS/MS PRM spectra of [D-Asp^3]MC-LR (**4**), -ER (**12**), (*m/z* 68–250)	S25
**Figure S23**	LC–HRMS/MS PRM spectra of [D-Asp^3]MC-LR (**4**), -ER (**12**), (*m/z* 250–410)	S26

Figure S24 LC–HRMS/MS PRM spectra of [D-Asp^3]MC-LR (**4**), -ER (**12**), (*m/z* 410–630) S27
Figure S25 LC–HRMS/MS PRM spectra of [D-Asp^3]MC-LR (**4**), -ER (**12**), (*m/z* 630–981) S28
Figure S26 LC–HRMS/MS PRM spectrum of [D-Asp^3]MC-EE (**13**) S29
Figure S27 LC–HRMS/MS PRM spectra of [D-Asp^3]MC-LA, -EE (**13**) S30
Figure S28 LC–HRMS/MS PRM spectra of [D-Asp^3]MC-LA, -EE (**13**), (*m/z* 80–350) S31
Figure S29 LC–HRMS/MS PRM spectra of [D-Asp^3]MC-LA, -EE (**13**), (*m/z* 330–670) S32
Figure S30 LC–HRMS/MS PRM spectra of [D-Asp^3]MC-LA, -EE (**13**), (*m/z* 560–980) S33
Figure S31 LC–HRMS/MS PRM spectrum of [D-Asp^3]MC-RW (**14**) S34
Figure S32 LC–HRMS/MS PRM spectra of sulfide conjugate of [D-Asp^3]MC-RR (**15**) S35
Figure S33 LC–HRMS/MS PRM spectra of sulfoxide conjugate of [D-Asp^3]MC-RR (**16**) S36
Figure S34 LC–MS chromatograms of an extract of NIVA-CYA 544 and a L. Victoria sample S37
Figure S35 LC–HRMS/MS PRM spectrum of [D-Asp^3]MC-RF (**18**) S38
Figure S36 LC–HRMS/MS PRM spectrum of [D-Asp^3]MC-RCit (**20**) S39
Figure S37 LC–HRMS/MS PRM spectra of [D-Asp^3]MC-RW, -RF, -RCit (**14**, **18**, **20**) S40
Figure S38 LC–HRMS chromatograms: esterification of NIVA-CYA 544 extract (**12**, **13**) S41
Figure S39 LC–HRMS/MS PRM spectra of GSH-conjugate of **1** (**19**) S42
Figure S40 LC–HRMS spectrum (positive mode) of a mixture of ^{15}N-labelled and unlabelled **1** S43
Figure S41 LC–HRMS spectrum (negative mode) of a mixture of ^{15}N-labelled and unlabelled **1** S44
Figure S42 Elemental composition of [D-Asp^3]MC-RR (**1**) by isotope profile analysis S45
Figure S43 Elemental composition of oxidized [D-Asp^3]MC-RR by isotope profile analysis S46
Figure S44 Elemental composition of [D-Asp^3]MC-LR (**4**) by isotope profile analysis S47
Figure S45 Elemental composition of [D-Asp^3,Mser^7]MC-LR (**11**) by isotope profile analysis S48
Figure S46 Elemental composition of [D-Asp^3]MC-ER (**12**) by isotope profile analysis S49
Figure S47 Elemental composition of [D-Asp^3]MC-EE (**13**) by isotope profile analysis S50
Figure S48 Elemental composition of [D-Asp^3]MC-RW (**14**) by isotope profile analysis S51
Figure S49 Elemental composition of the sulfide conjugate of **1** (**15**) by isotope profile analysis S52
Figure S50 Elemental composition of sulfoxide conjugate **16** by isotope profile analysis S53
Figure S51 Elemental composition of [D-Asp^3]MC-RY (**17**) by isotope profile analysis S54
Figure S52 Elemental composition of [D-Asp^3]MC-RF (**18**) by isotope profile analysis S55
Figure S53 Elemental composition of the GSH conjugate of **1** (**19**) by isotope profile analysis S56
Figure S54 Elemental composition of [D-Asp^3]MC-RCit (**20**) by isotope profile analysis S57
Figure S55 LC–HRMS chromatograms and spectra of [D-Asp^3]MC-RR cysteine conjugate S58
Figure S56 LC–HRMS chromatograms and spectra of [D-Asp^3]MC-RR cysteine conjugate S59
Figure S57 Most probable elemental composition of [D-Asp^3]MC-RR cysteine conjugate S60
Figure S58 HRMS/MS spectra of unlabelled and ^{15}N-labelled sulfide conjugate of **1** (**15**) S61
Figure S59 Expansion of the HRMS/MS spectra of unlabelled and ^{15}N-labelled **15** S62
Figure S60 Expansion of the HRMS/MS spectra of unlabelled and ^{15}N-labelled **15** S63
Figure S61 HRMS/MS spectra of unlabelled and ^{15}N-labelled sulfoxide conjugate of **1** (**16**) S64
Figure S62 Expansion of the HRMS/MS spectra of unlabelled and ^{15}N-labelled **16** S65
Figure S63 Expansion of the HRMS/MS spectra of unlabelled and ^{15}N-labelled **16** S66
Figure S64 HRMS/MS spectra of unlabelled and ^{15}N-labelled [D-Asp^3]MC-RCit (**20**) S67
Figure S65 HRMS/MS spectra of unlabelled and ^{15}N-labelled [D-Asp^3]MC-RW (**14**) S68
Figure S66 HRMS/MS spectra of unlabelled and ^{15}N-labelled [D-Asp^3]MC-RF (**18**) S69
Figure S67 HRMS/MS spectra of unlabelled and ^{15}N-labelled [D-Asp^3]MC-RR (**1**) S70
Literature cited S71

Table S1. Retention times of microcystins detected in *P. prolifica* NIVA-CYA 544 or authentic samples analysed using the three LC–MS methods.

	Microcystin	Confidence	t_R (min)		
			method A	method B	method C
**1**	[D-Asp^3]MC-RR	confirmed	2.81	4.28	—
**4**	[D-Asp^3]MC-LR	confirmed	5.72	7.04	4.46
**11**	[D-Asp^3,Mser^7]MC-RR	probable	2.59	4.14	1.82
**12**	[D-Asp^3]MC-ER	probable	3.63	6.24	3.53
**13**	[D-Asp^3]MC-EE	probable	4.99	11.62	3.93
**14**	[D-Asp^3]MC-RW	probable	10.01	9.72	8.40
**15**	Sulfide conjugate of **1**	tentative	9.85[e]	7.50	7.47
**16**	**15**-sulfoxide	tentative	6.50[f]	6.53	—
**17**	[D-Asp^3]MC-RY	probable	7.16	8.73	—
**18**	[D-Asp^3]MC-RF	probable	9.94	9.82	—
**19**	GSH-conjugate of **1**	confirmed	2.05	4.09	—
**20**	[D-Asp^3]MC-RCit	probable	3.44	6.79	—
—	Cys-conjugate of **1**	tentative	—	3.74	—
—	oxidized **1**	tentative	—	4.09	—
**2**	MC-RR	standard	3.12	4.63	2.31
**3**	MC-YR	standard	5.22	7.14	4.09
**5**	[Dha^7]MC-LR	standard	—	7.28	4.67
**6**	MC-LR	standard	5.84	7.29	4.58
**7**	MC-LA	standard	9.35	15.10	8.39
**8**	MC-LY	standard	10.65	15.34	9.67
**9**	MC-LW	standard	13.83	17.20	12.80
**10**	MC-LF	standard	13.91	17.85	12.89
**23**	[D-Asp^3,Dhb^7]MC-RR	standard	2.97	—	2.19
**24**	[D-Asp^3]MC-EHar	tentative	—	6.55	—
**25**	[D-Asp^3]MC-RY(OMe)	tentative	—	8.89	—
**26**	[D-Asp^3]MC-HarY	tentative	—	8.90	—

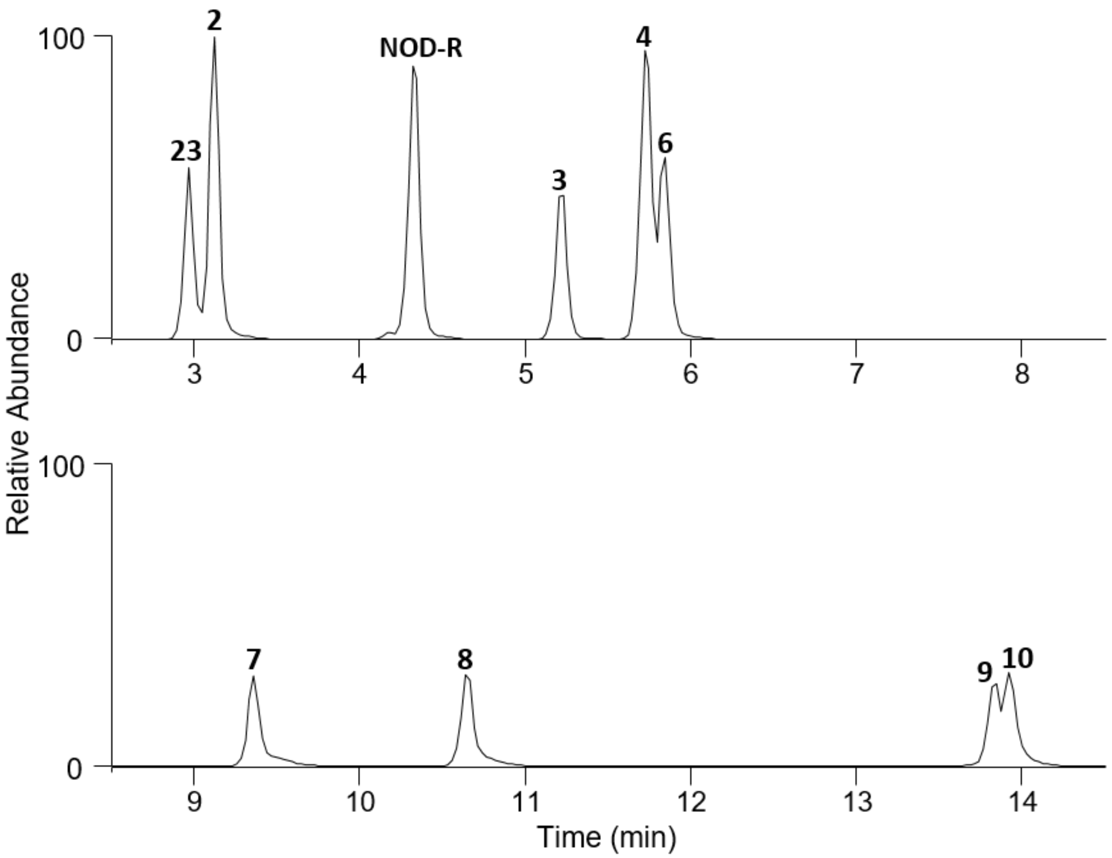

Figure S1. LC–HRMS (method A) full scan chromatogram in positive ion mode of a standard mixture (200 ng/mL) of 9 microcystins (MCs) and Nodularin-R, used to optimize the method: [D-Asp^{3},Dhb^{7}]MC-RR (**23**) (wrongly labelled by Enzo as [D-Asp^{3}]MC-RR), MC-RR (**2**), NOD-R, MC-YR (**3**), [D-Asp^{3}]MC-LR (**4**), MC-LR (**6**), MC-LA (**7**), MC-LY (**8**), MC-LW (**9**), MC-LF (**10**). The chromatogram was extracted at the specified *m*/*z* of standard compounds, as reported in Figure 1, with 5 ppm tolerance.

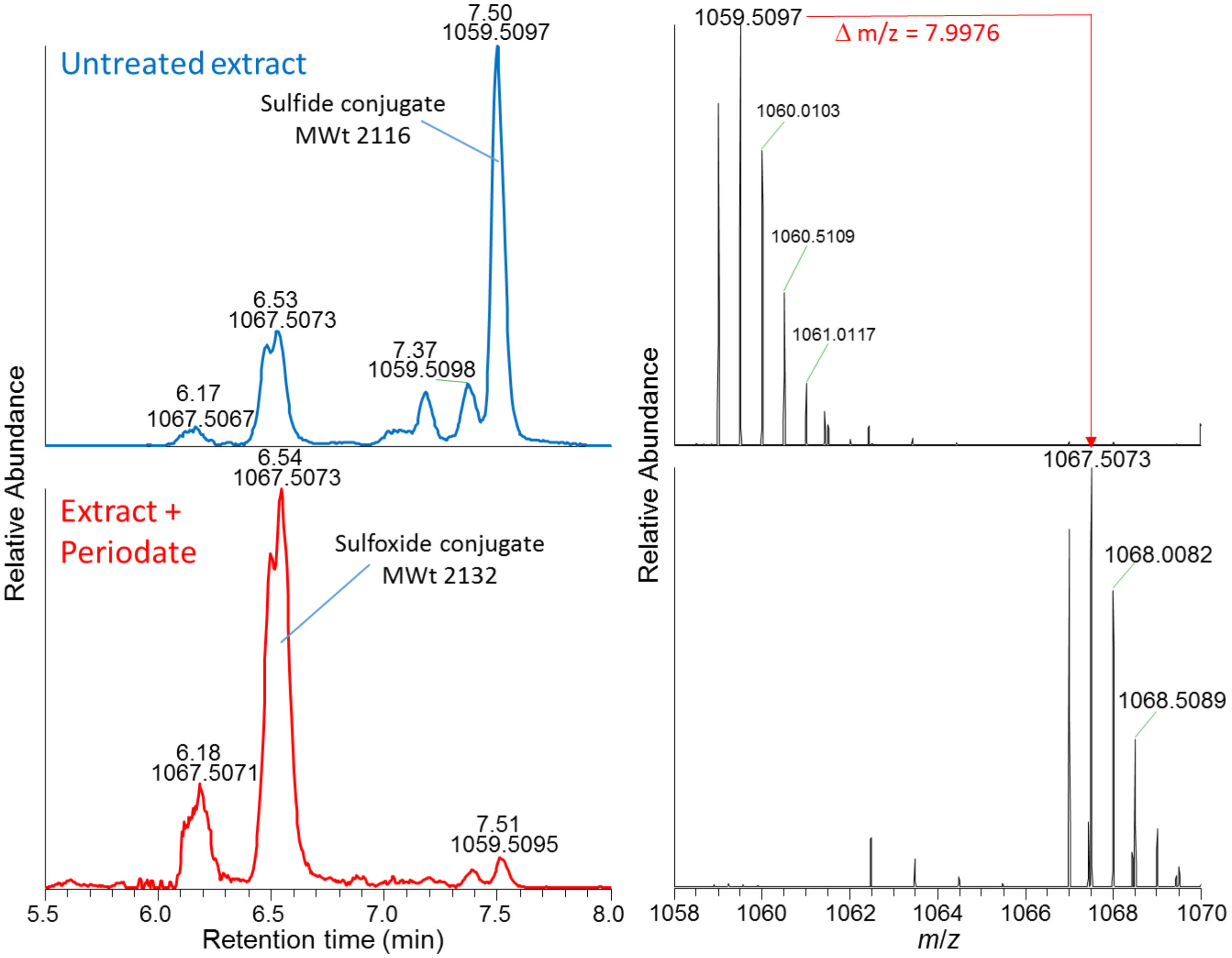

Figure S2. Left, LC−HRMS chromatograms (method B) before (top left) and after (bottom left) treatment with sodium periodate. Both chromatograms are extracted at both *m*/*z* 1059.0088 and 1067.0060, and show complete conversion of sulfide **15** to sulfoxide **16**. To the right are the mass spectra of the main peak the sulfide (top right) and sulfoxide (bottom right), showing a change in *m*/*z* corresponding to addition of one oxygen atom (theoretical Δ *m*/*z* = 7.9975).

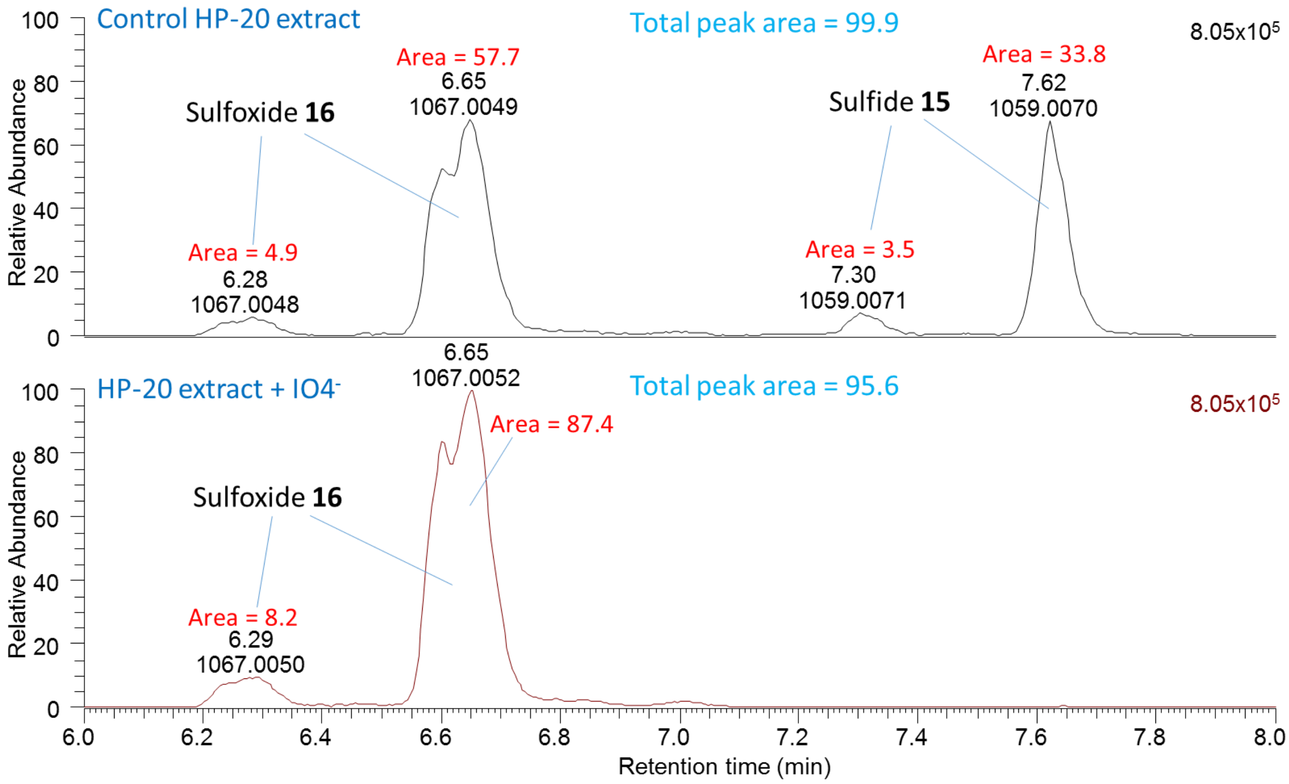

Figure S3. LC–HRMS chromatograms before (top) and 15 min after (bottom) treatment with sodium periodate. Both chromatograms are extracted at both *m*/*z* 1059.0088 and 1067.0060, and displayed with the same vertical scale. Equivalent injection volumes were used for both chromatograms, and integration of the peaks (peak areas in arbitrary units) shows complete conversion of sulfide **15** to sulfoxide **16**.

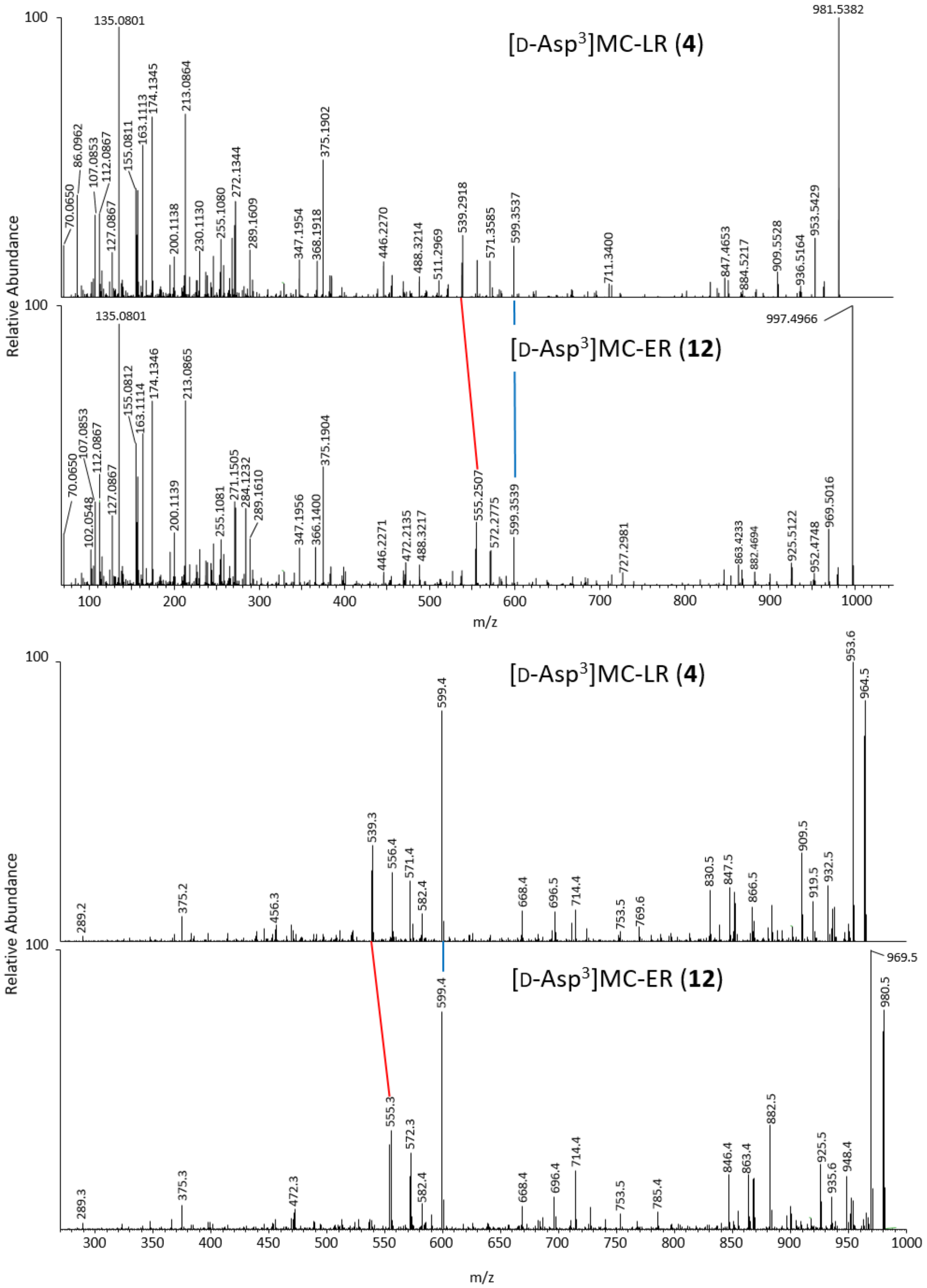

Figure S4. Top, LC–HRMS/MS (method A) product ion spectra, and; bottom, LC–MS^2 (method C) product ion spectra from collision-induced fragmentation of the $[M + H]^+$ ions of [D-Asp^3]MC-LR (**4**) and [D-Asp^3]MC-ER (**12**). The blue lines link examples of conserved fragments while the red lines link examples of fragments shifted by the exact difference between the masses of L and E (15.9585).

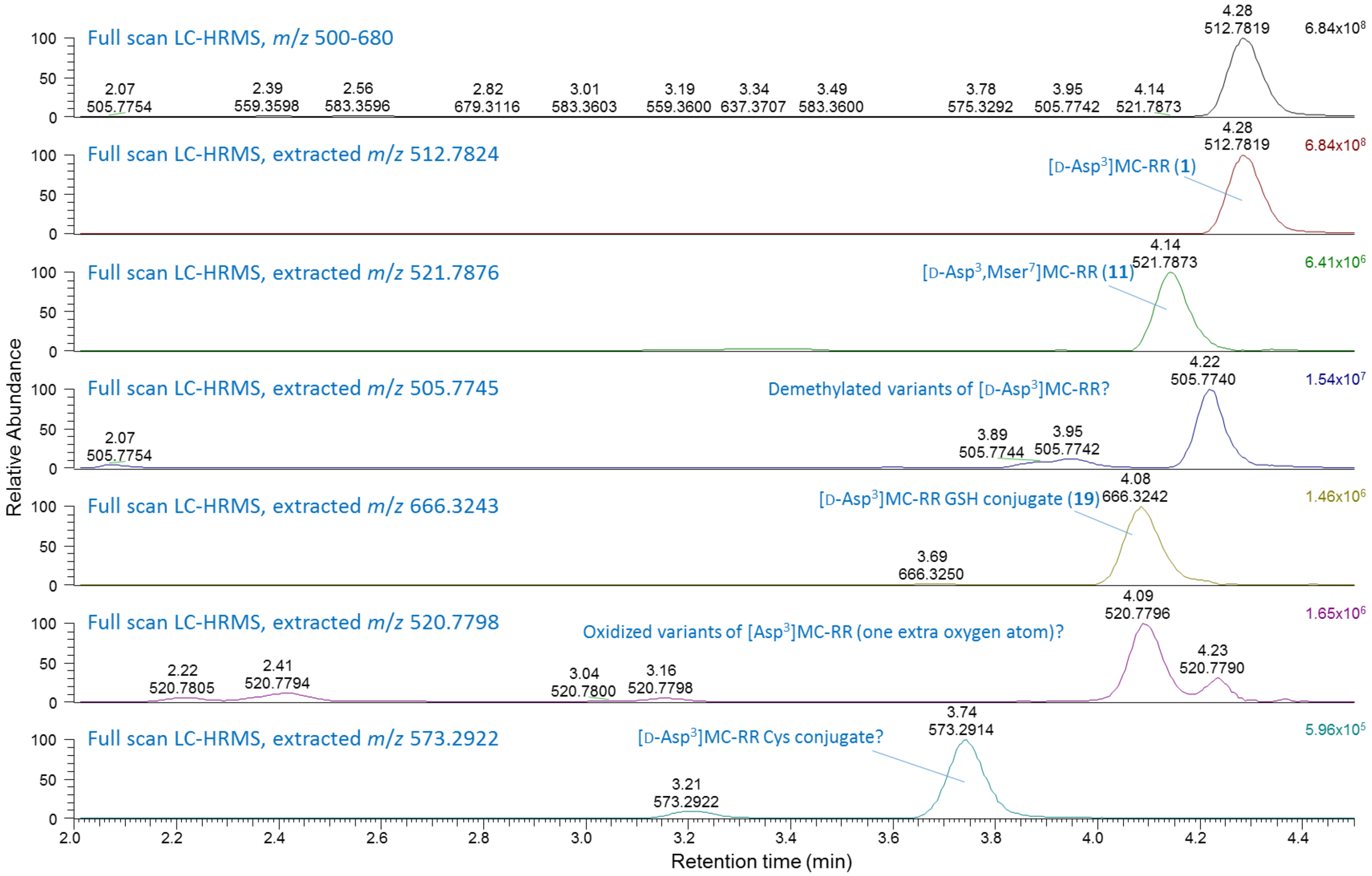

Figure S5. LC−HRMS (method B) chromatograms (2.0–4.5 min) in positive ionization mode of an HP-20 extract of NIVA-CYA 544, showing the polar doubly-charged microcystins. Extracted ion chromatograms were at the specified *m*/*z* with 5 ppm tolerance.

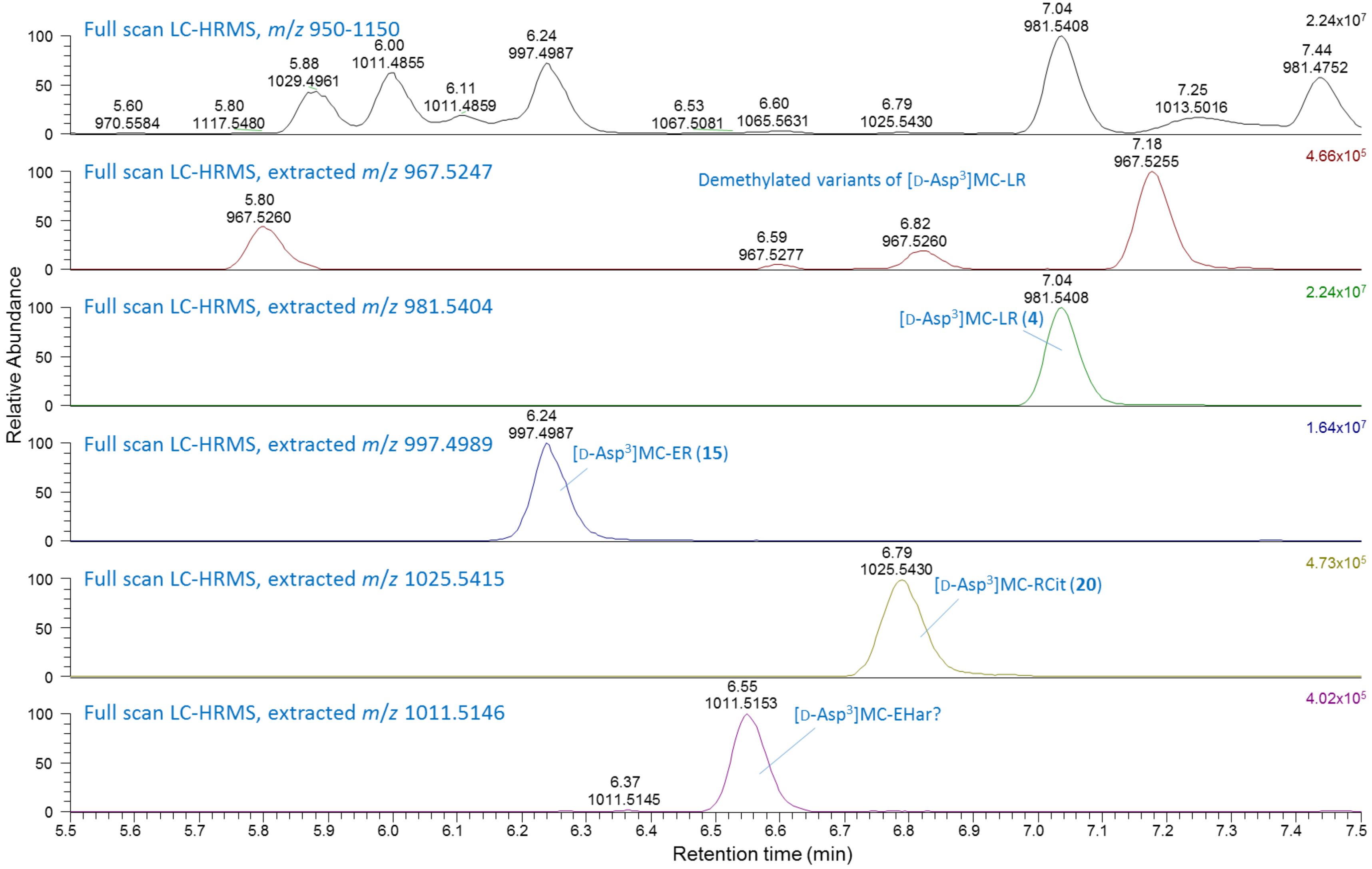

Figure S6. LC−HRMS (method B) chromatograms (5.5–7.5 min) in positive ionization mode of an HP-20 extract of NIVA-CYA 544, showing the less polar singly-charged microcystins. Extracted ion chromatograms were at the specified *m*/*z* with 5 ppm tolerance.

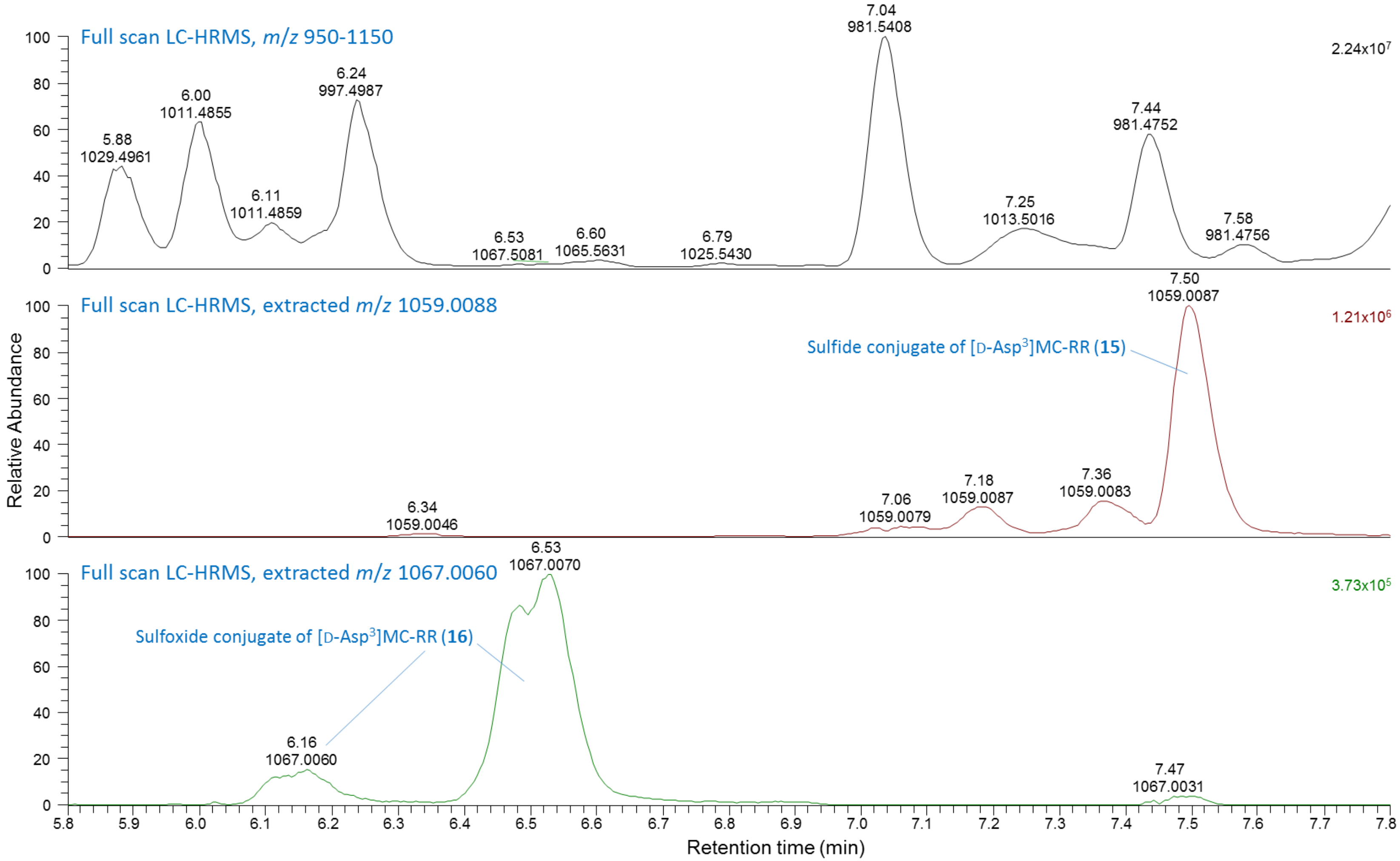

Figure S7. LC−HRMS (method B) chromatograms (5.8–7.8 min) in positive ionization mode of an HP-20 extract of NIVA-CYA 544, showing the less polar doubly-charged microcystin conjugates. Extracted ion chromatograms were at the specified *m*/*z* with 5 ppm tolerance. Note the presence of a major and a minor stereoisomer of **16**, each of which appears to be present as a pair of sulfoxide diastereoisomers.

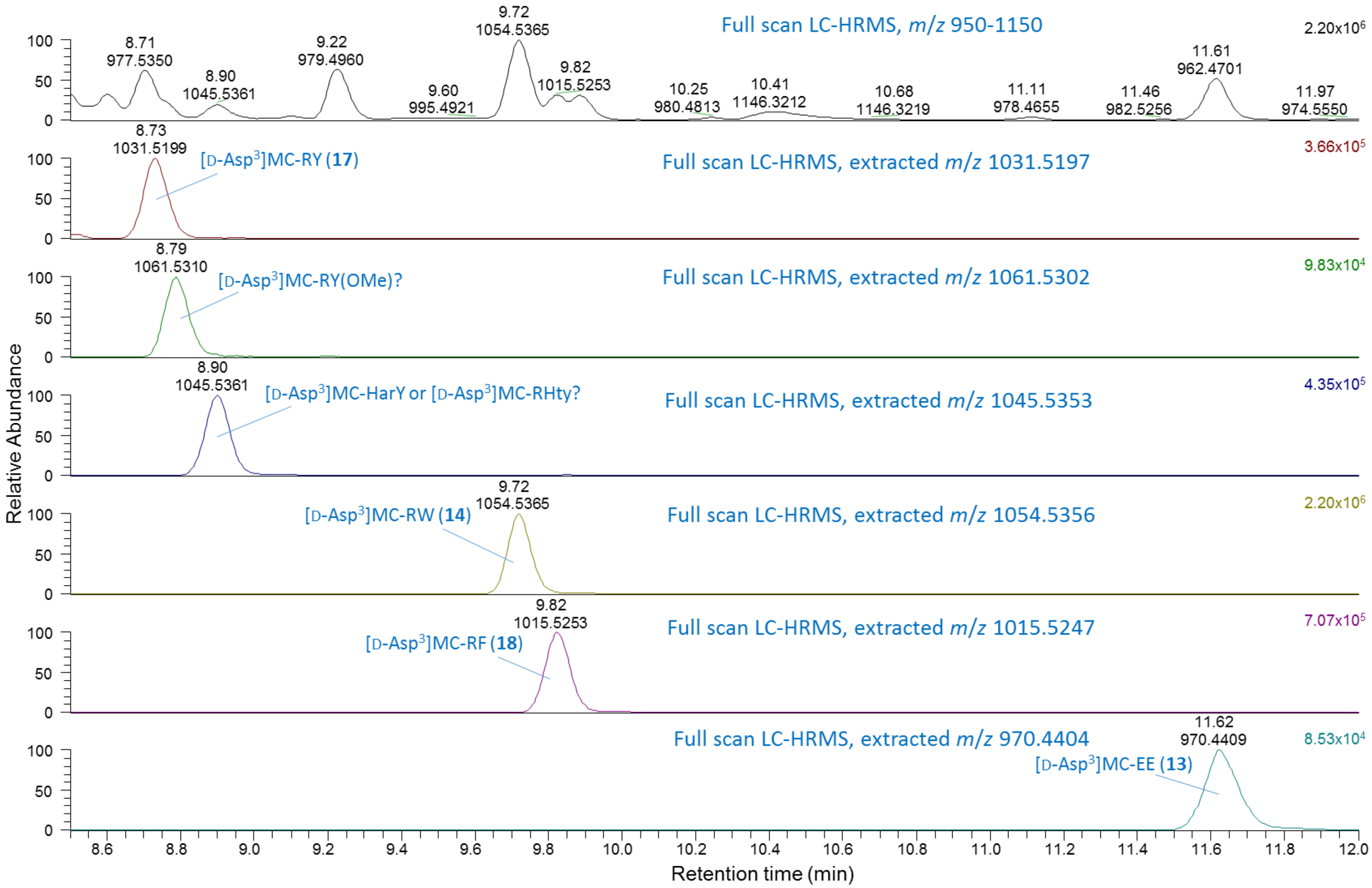

Figure S8. LC−HRMS (method B) chromatograms (8.5–12 min) in positive ionization mode of an HP-20 extract of NIVA-CYA 544, showing the least polar singly-charged microcystins. Extracted ion chromatograms were at the specified *m*/*z* with 5 ppm tolerance.

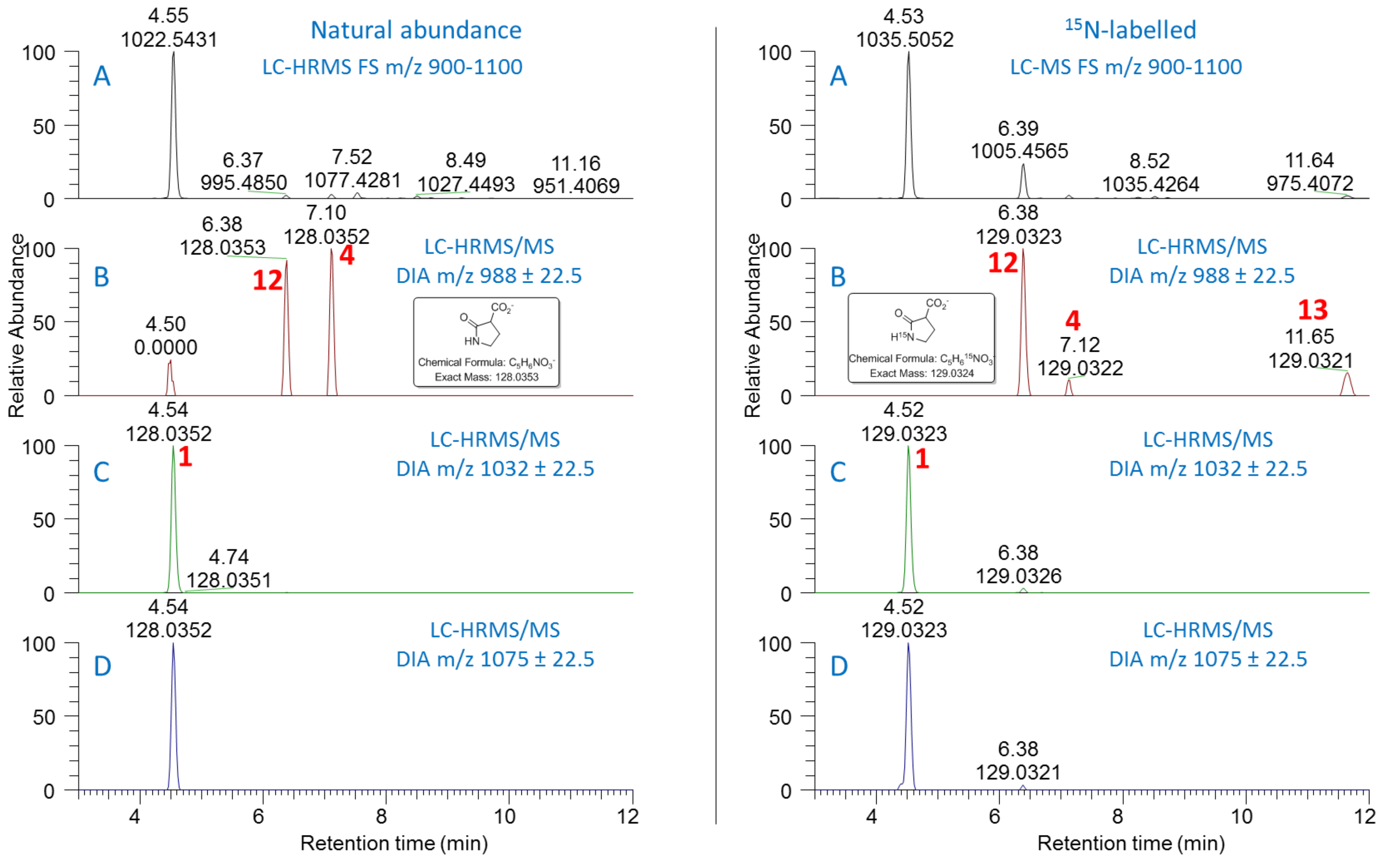

Figure S9. Negative ionization LC−MS/MS FS/DIA chromatograms of an extract of NIVA-CYA 544 at natural abundance (left) and in a ^{15}N-labelled culture (right), extracted at *m/z* 128.0353 ($C_5H_6{}^{14}NO_3^-$, left) and 129.0324 ($C_5H_6{}^{15}NO_3^-$, right) from the Glu^6-moiety.

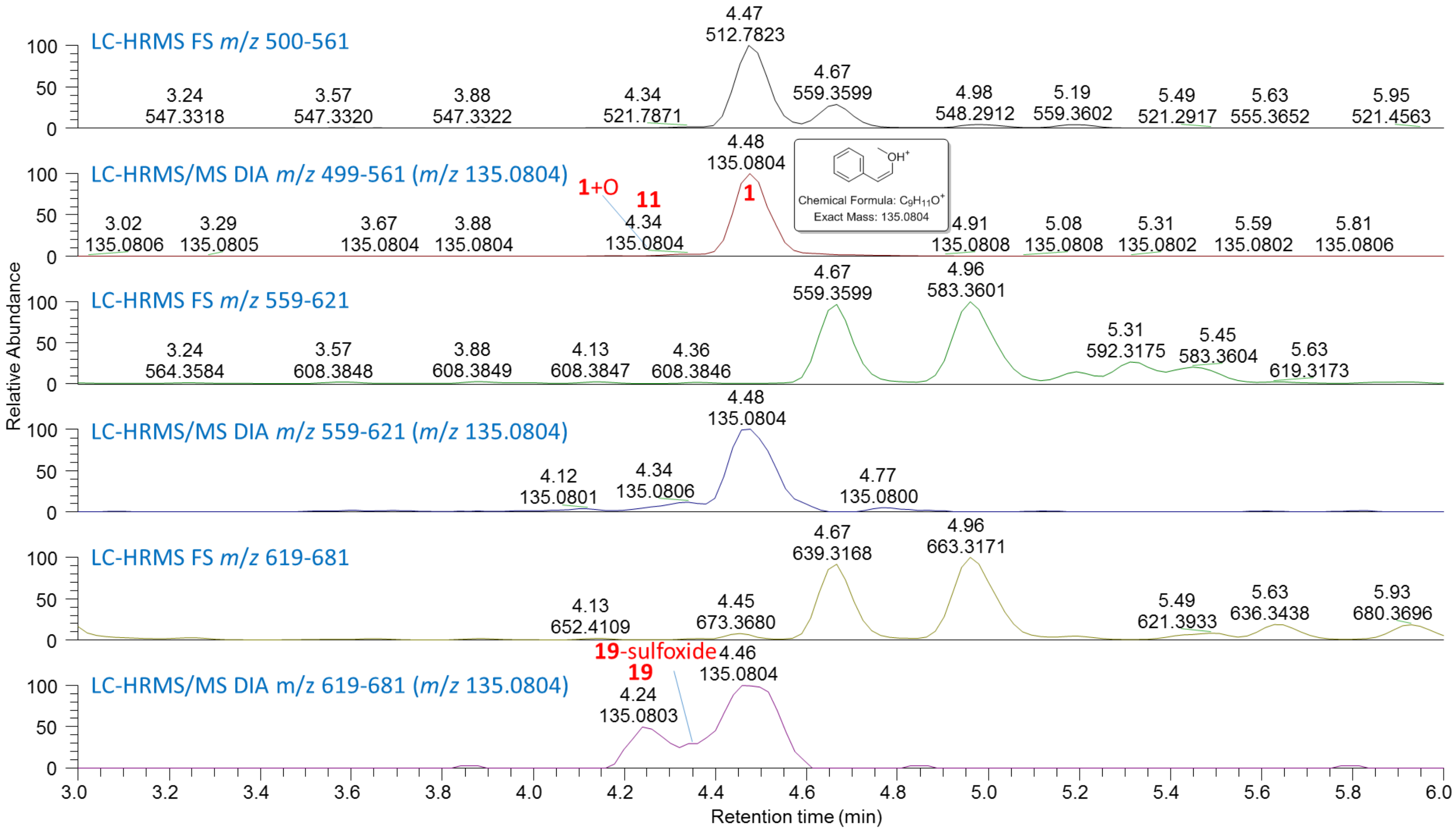

Figure S10. Positive ionization LC−MS/MS FS/DIA chromatograms of an extract of NIVA-CYA 544 at natural abundance extracted at *m/z* 135.0804 ($C_9H_{11}O^+$) from the Adda[5]-moiety.

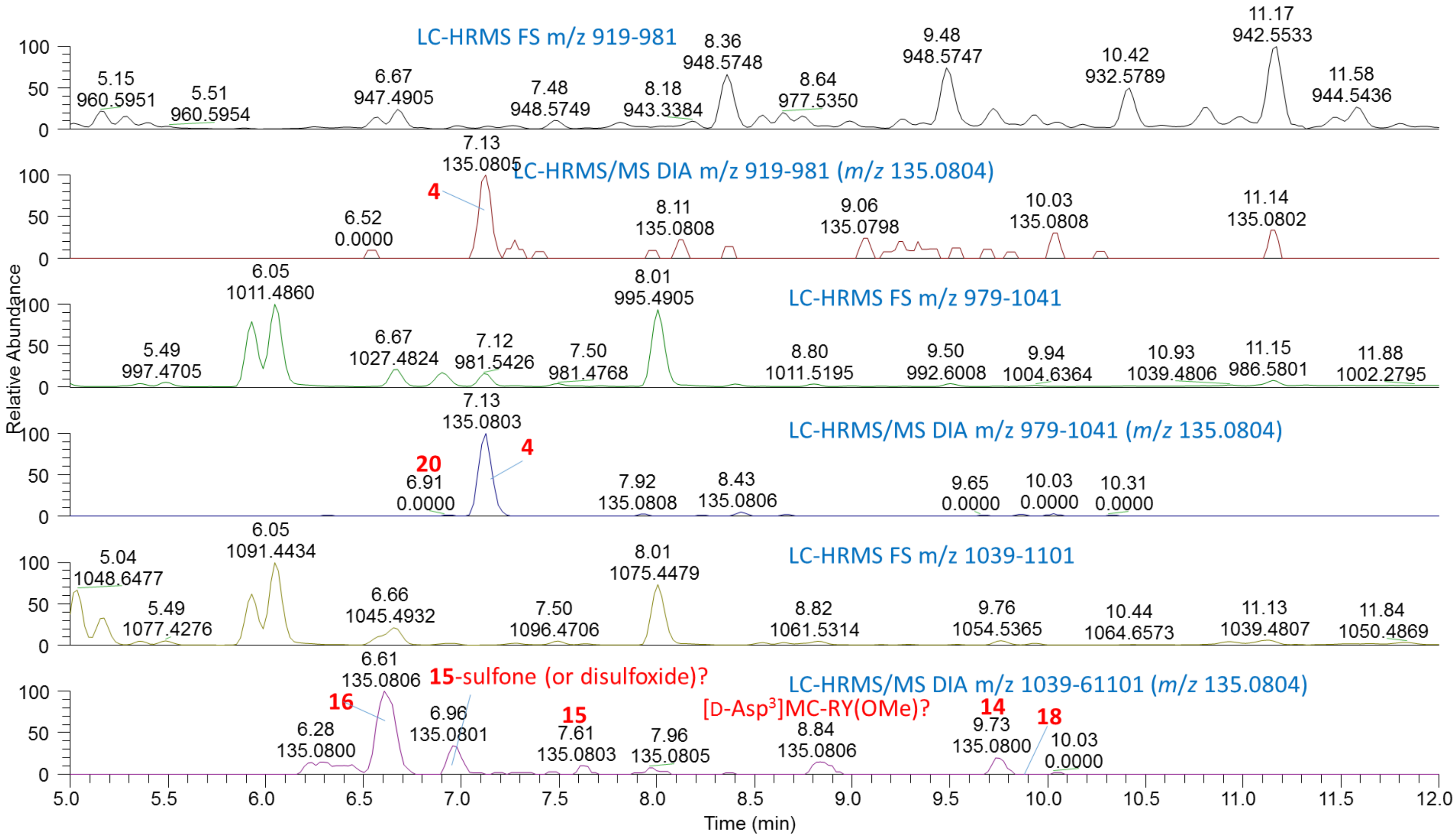

Figure S11. Positive ionization LC−MS/MS FS/DIA chromatograms of an extract of NIVA-CYA 544 at natural abundance extracted at *m/z* 135.0804 ($C_9H_{11}O^+$) from the Adda[5]-moiety.

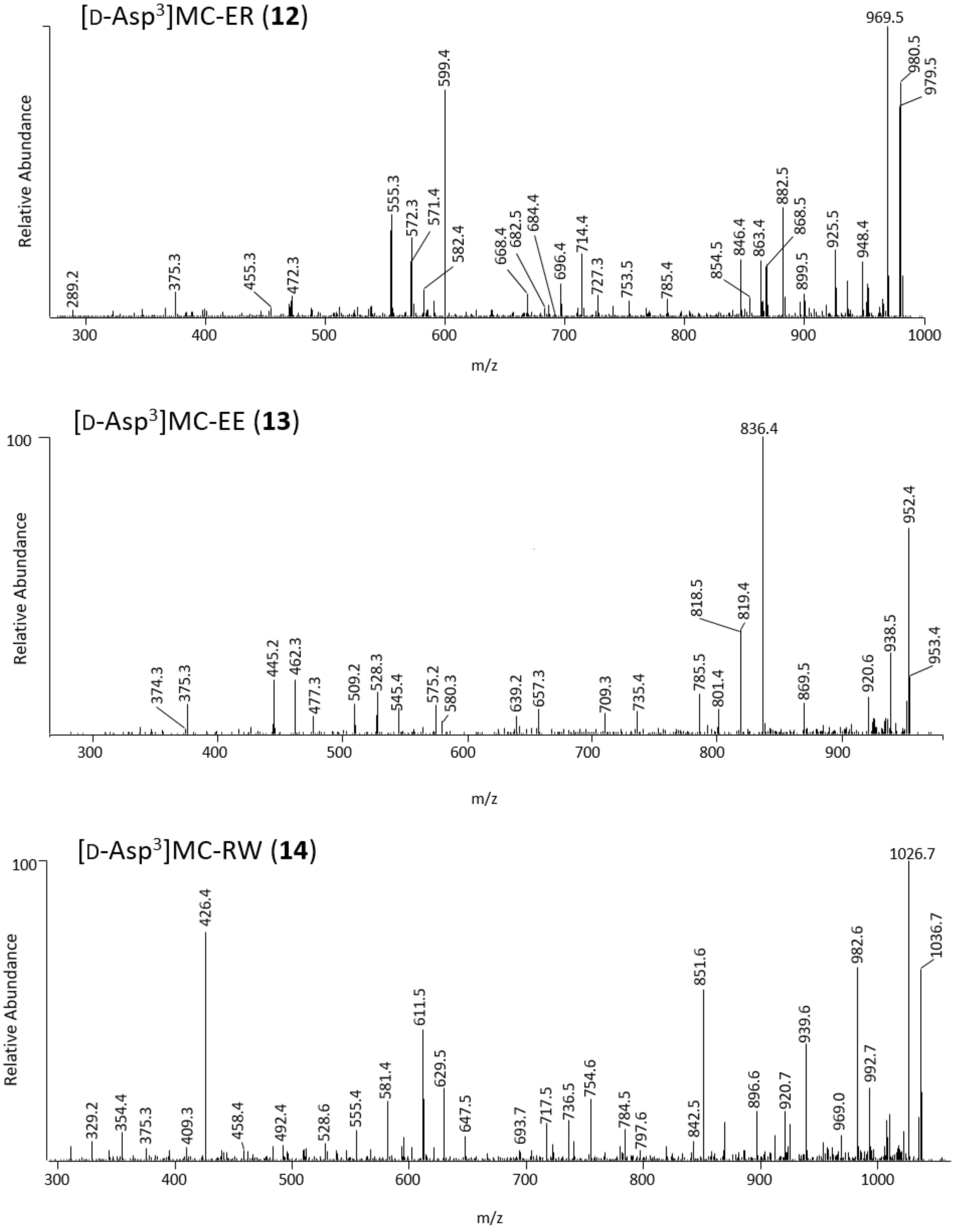

Figure S12. LC–MS^2 (method C) product ion spectra from collision-induced fragmentation of the [M + H]$^+$ ions of [D-Asp^3]MC-ER (**12**), [D-Asp^3]MC-EE (**13**) and [D-Asp^3]MC-RW (**14**).

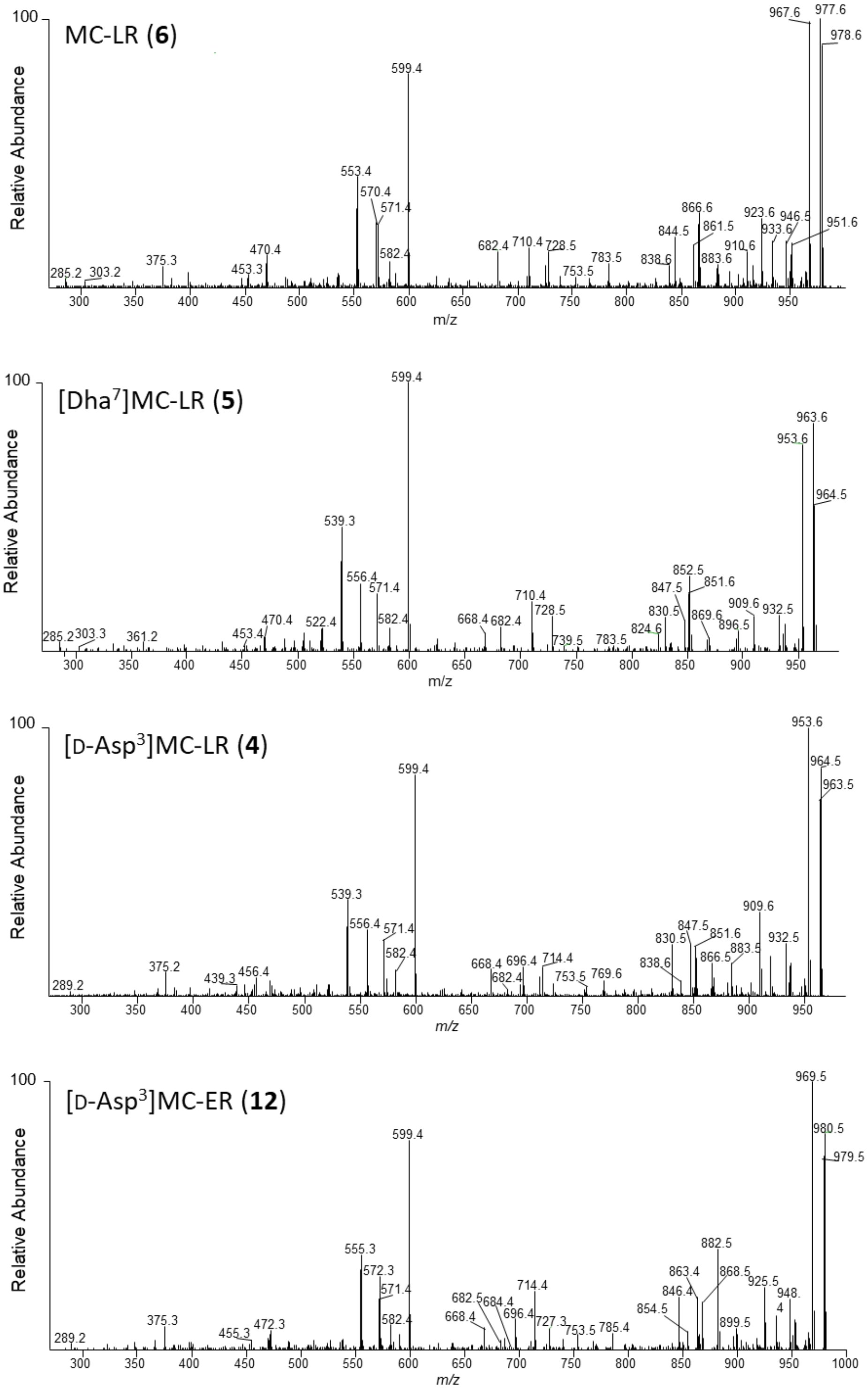

Figure S13. LC–MS/MS (method C) positive product ion spectra from collision-induced fragmentation of the $[M + H]^+$ ions of MC-LR (**6**), [Dha^7]MC-LR (**5**), [D-Asp^3]MC-LR (**4**) and [D-Asp^3]MC-ER (**12**).

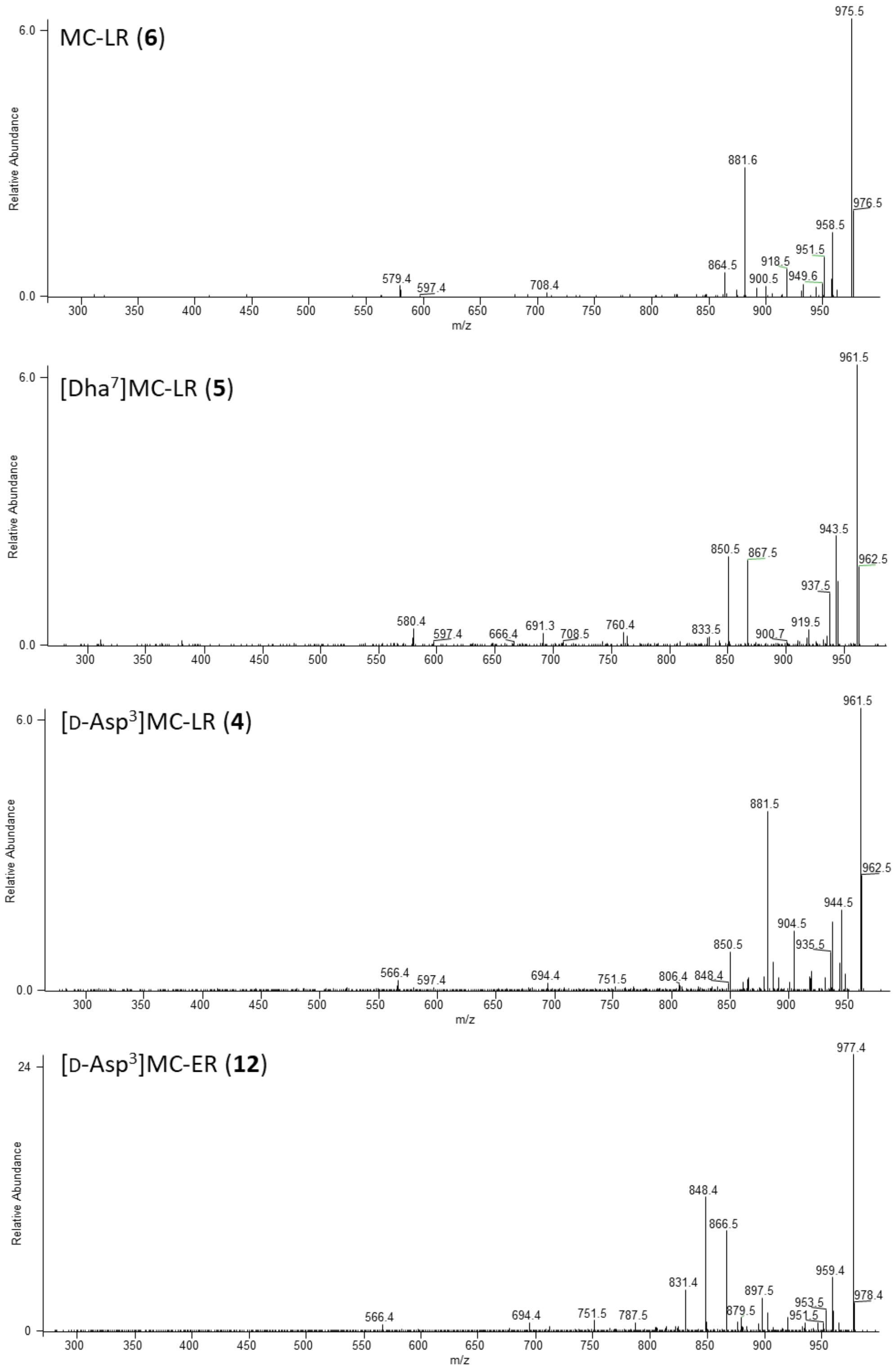

Figure S14. LC–MS/MS (method C) negative product ion spectra from collision-induced fragmentation of the $[M - H]^-$ ions of MC-LR (**6**), [Dha^7]MC-LR (**5**), [D-Asp^3]MC-LR (**4**) and [D-Asp^3]MC-ER (**12**).

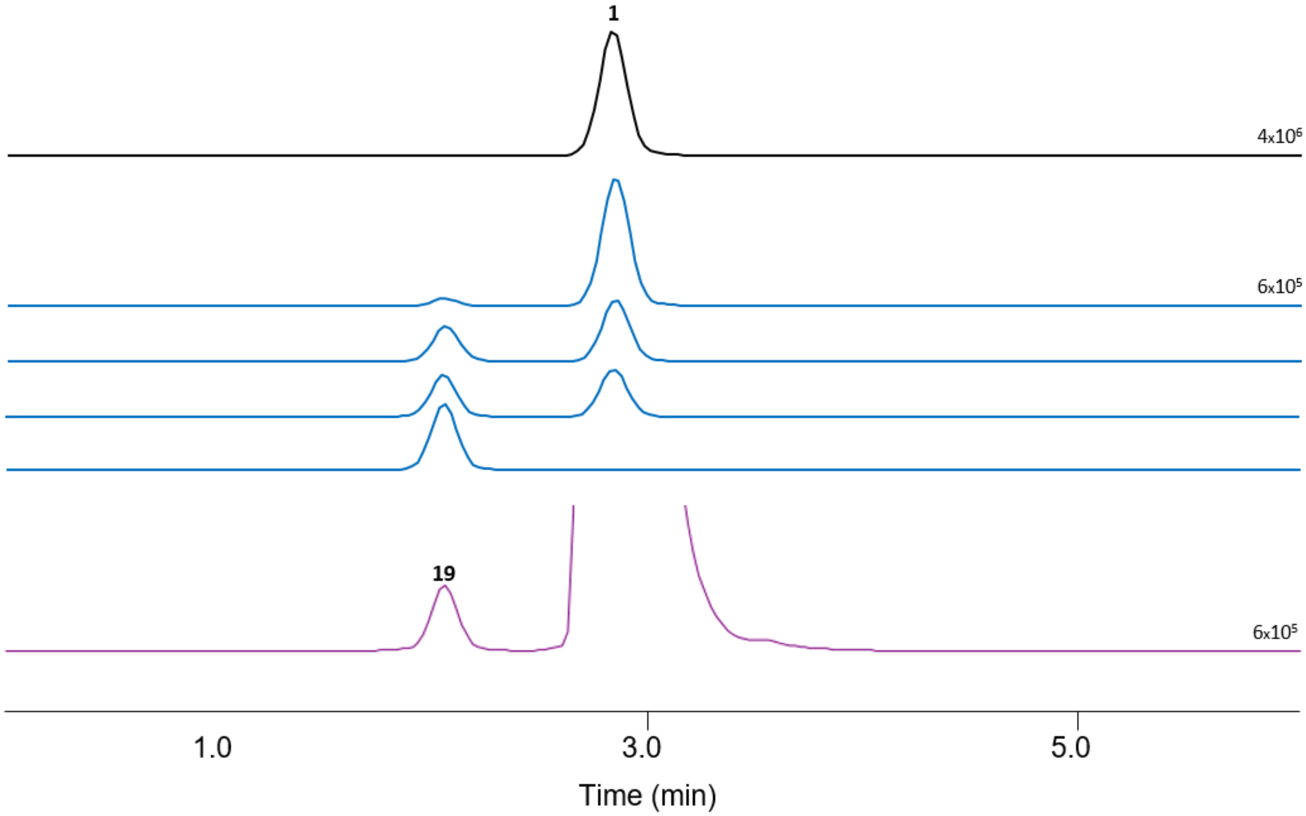

Figure S15. Extracted ion (at *m*/*z* for **1** and **19**) LC−HRMS (method A) chromatograms of: a standard solution of [D-Asp^3]MC-RR (**1**) (black); the gradual conversion of standard [D-Asp^3]MC-RR (**1**) to the GSH-conjugate of **1** (**19**) by reaction with glutathione in weakly basic solution (blue); an extract of NIVA-CYA 544 showing extracted *m*/*z* corresponding to $[M + H]^+$ for the GSH-conjugate of **1** (**19**) and [D-Asp^3]MC-RR (**1**) (purple). Results of the reaction (blue) supported the identification of **19** as the GSH-conjugate of the major microcystin congener **1**, together with elemental composition calculation and comparison of the LC–HRMS characteristics of the products with those of **19** in the culture extract. LC-HRMS/MS spectra of natural **19** in the culture extract and semi-synthetic **19** produced by reaction of **1** with GSH are shown in Figure S39.

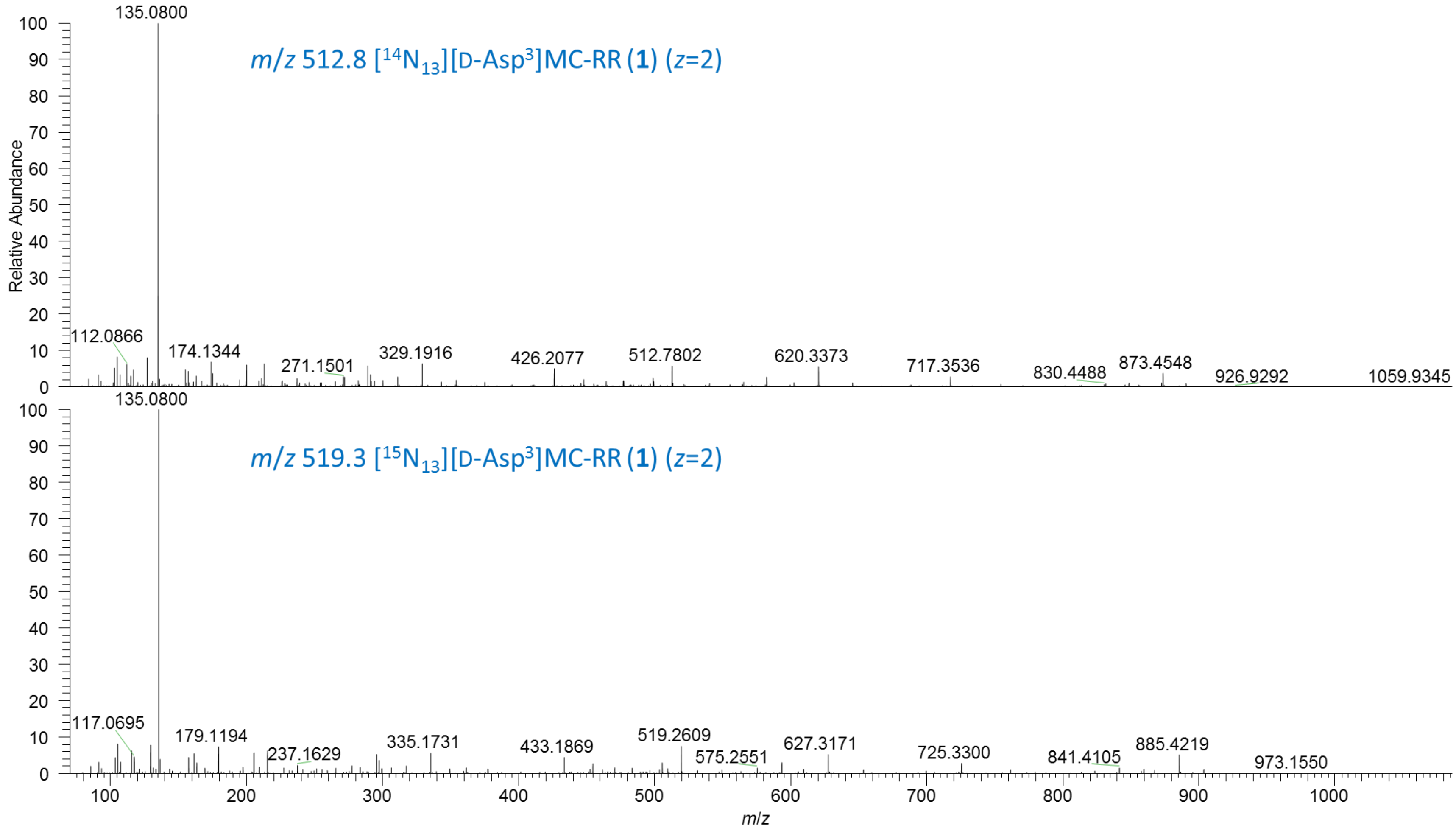

Figure S16. LC–HRMS/MS PRM spectra (method B) of $[M + 2H]^{2+}$ of [D-Asp^3]MC-RR (**1**) at *m*/*z* 512.8 (top), and of nitrogen-15 labelled [D-Asp^3]MC-RR (**1**) at *m*/*z* 519.3 (bottom).

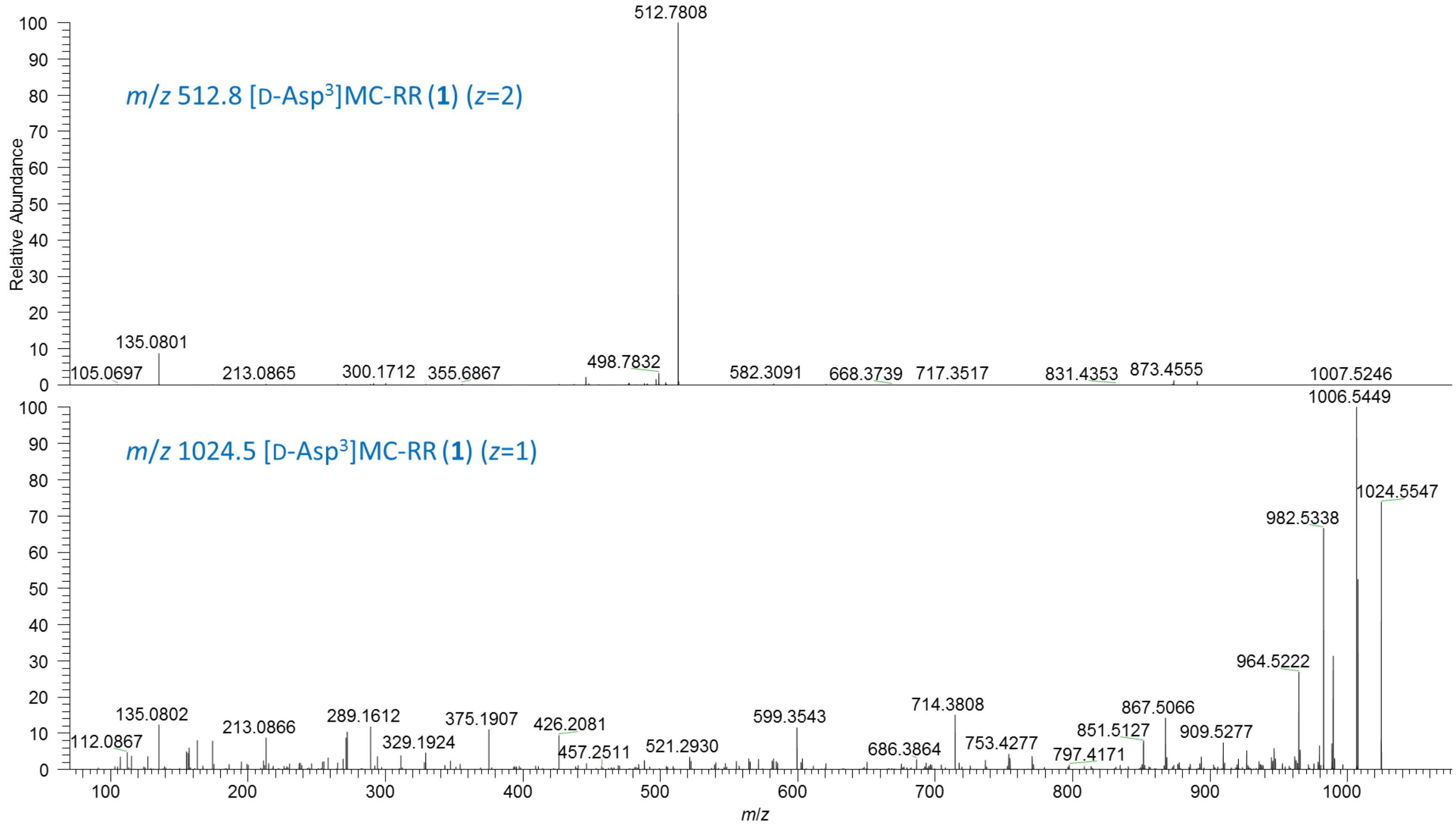

Figure S17. LC–HRMS/MS PRM spectra (method B) of $[M + 2H]^{2+}$ of [D-Asp^3]MC-RR (**1**) at *m/z* 512.8 (top), and of $[M + H]^+$ of [D-Asp^3]MC-RR (**1**) at *m/z* 1024.5 (bottom).

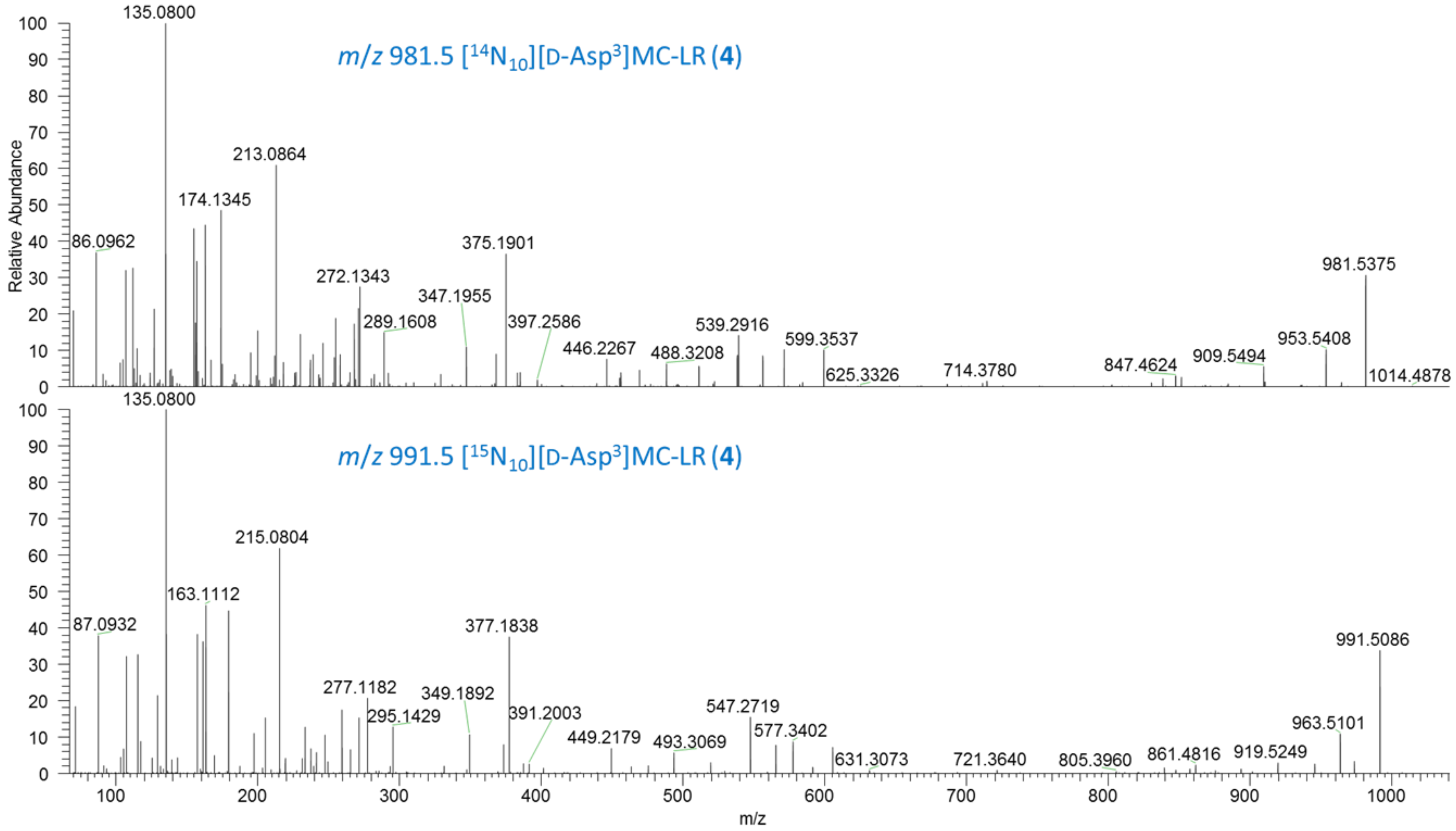

Figure S18. LC–HRMS/MS PRM spectra (method B) of $[M + H]^+$ of [D-Asp^3]MC-LR (**4**) at *m/z* 981.5 (top), and of nitrogen-15 labelled [D-Asp^3]MC-LR (**4**) at *m/z* 991.5 (bottom).

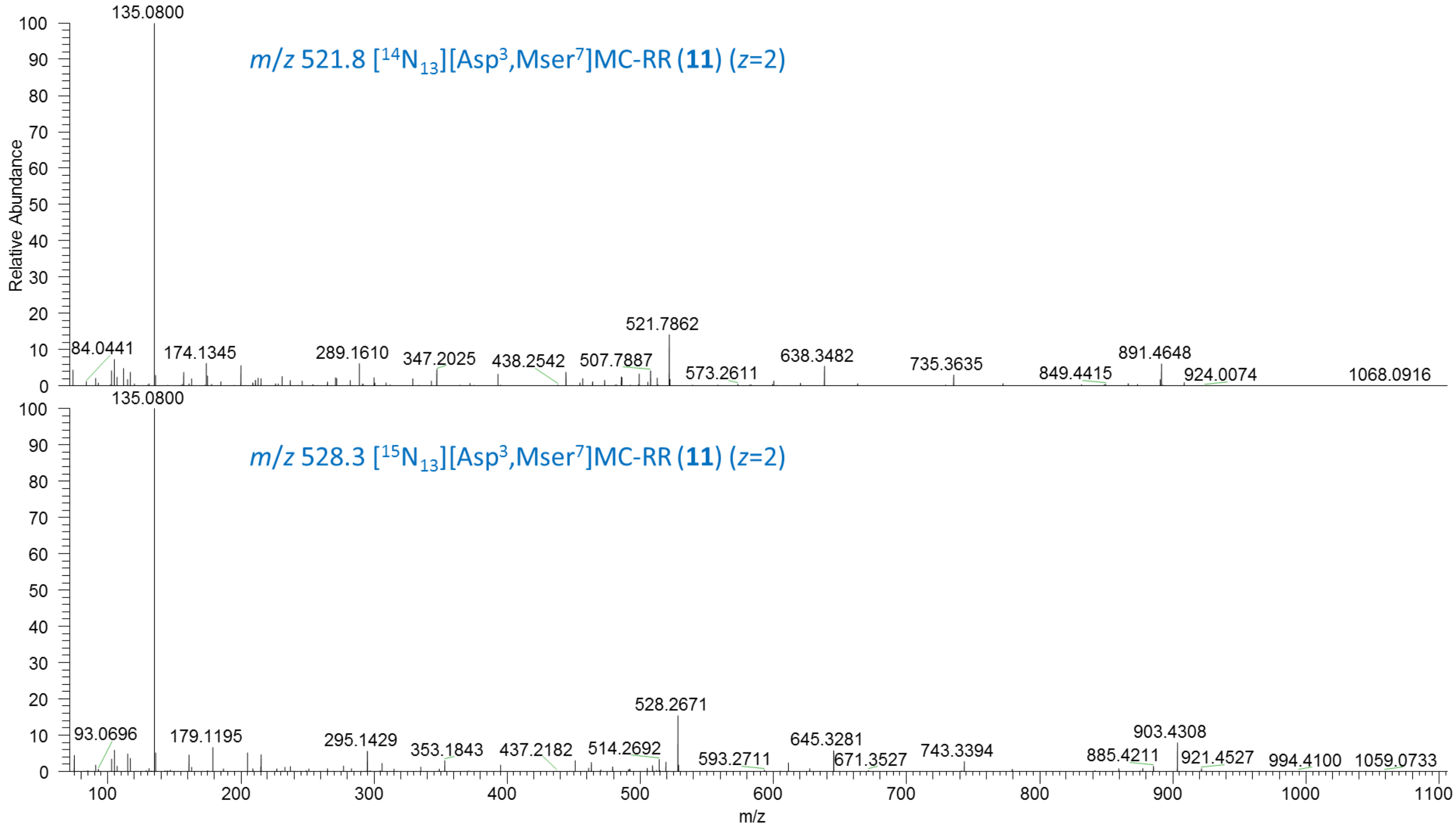

Figure S19. LC–HRMS/MS PRM spectra (method B) of $[M + 2H]^{2+}$ of [D-Asp^3,Mser^7]MC-RR (**11**) at *m*/*z* 521.8 (top), and of nitrogen-15 labelled [D-Asp^3]MC-RR (**11**) at *m*/*z* 528.3 (bottom).

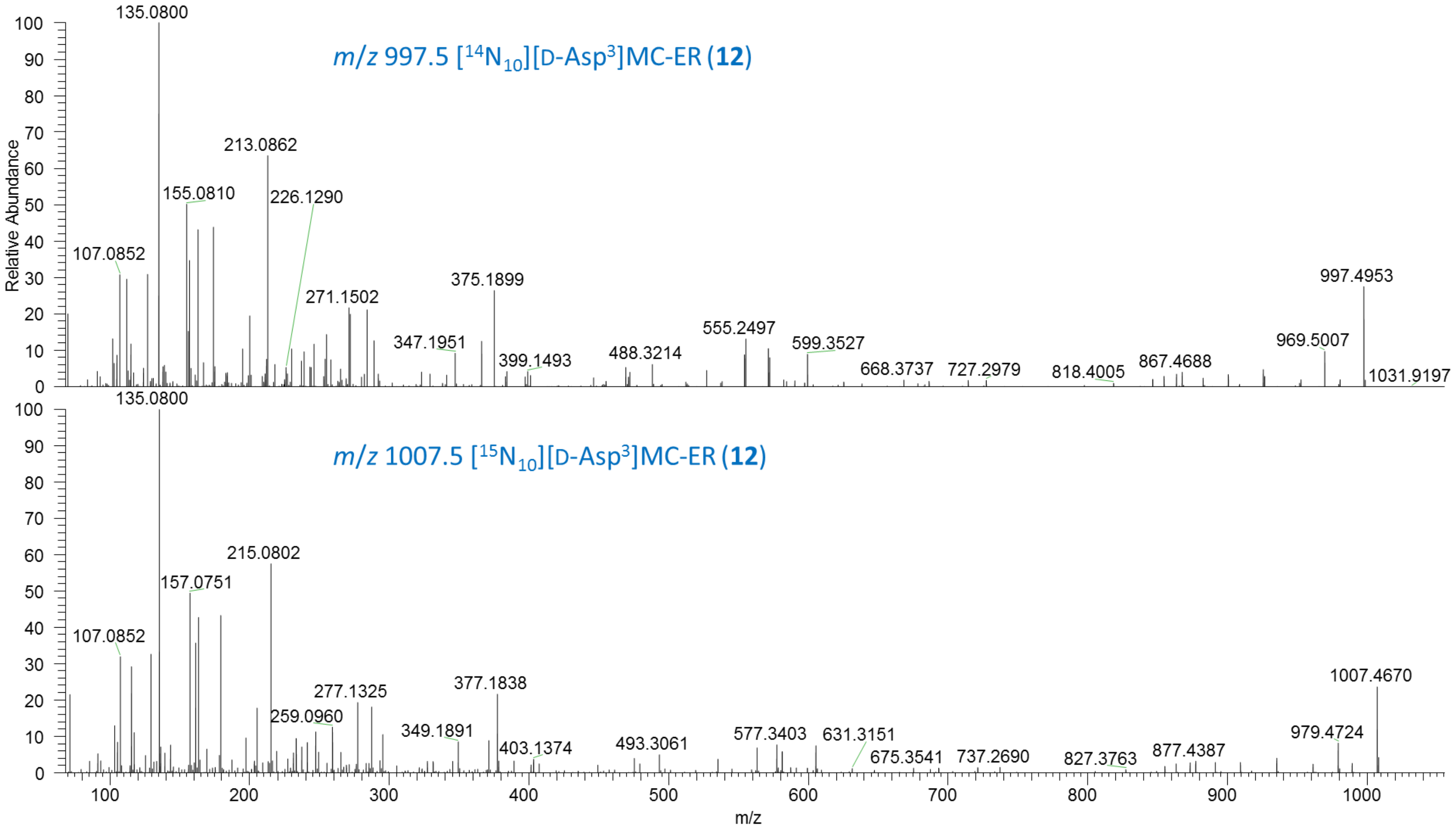

Figure S20. LC–HRMS/MS PRM spectra (method B) of $[M + H]^+$ of [D-Asp^3]MC-ER (**12**) at *m*/*z* 997.5 (top), and of nitrogen-15 labelled [D-Asp^3]MC-ER (**12**) at *m*/*z* 1007.5 (bottom).

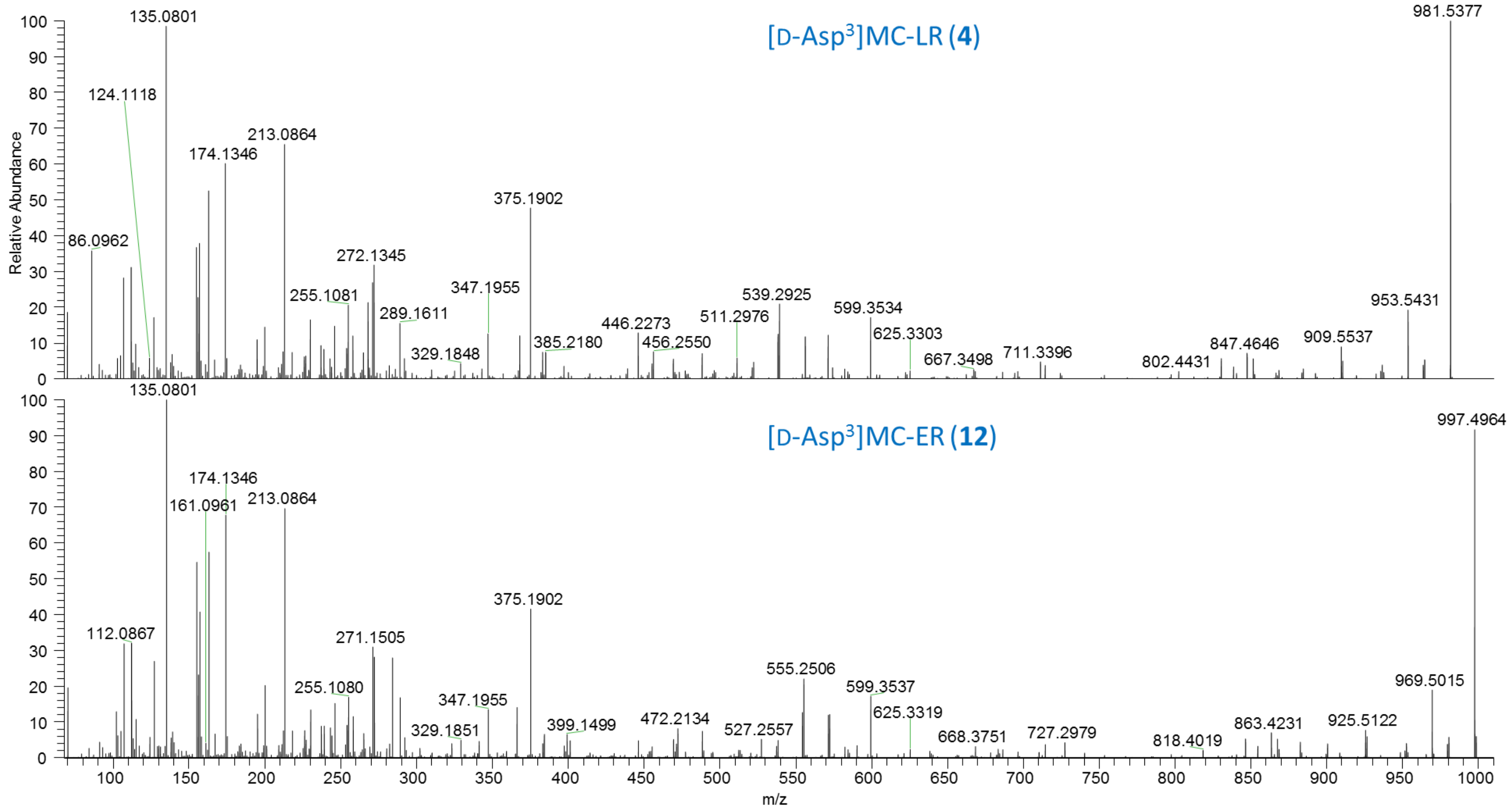

Figure S21. LC–HRMS/MS PRM spectra (method B) of $[M + H]^+$ of [D-Asp^3]MC-LR (**4**) at *m/z* 981.5 (top), and of [D-Asp^3]MC-ER (**12**) at *m/z* 997.5 (bottom).

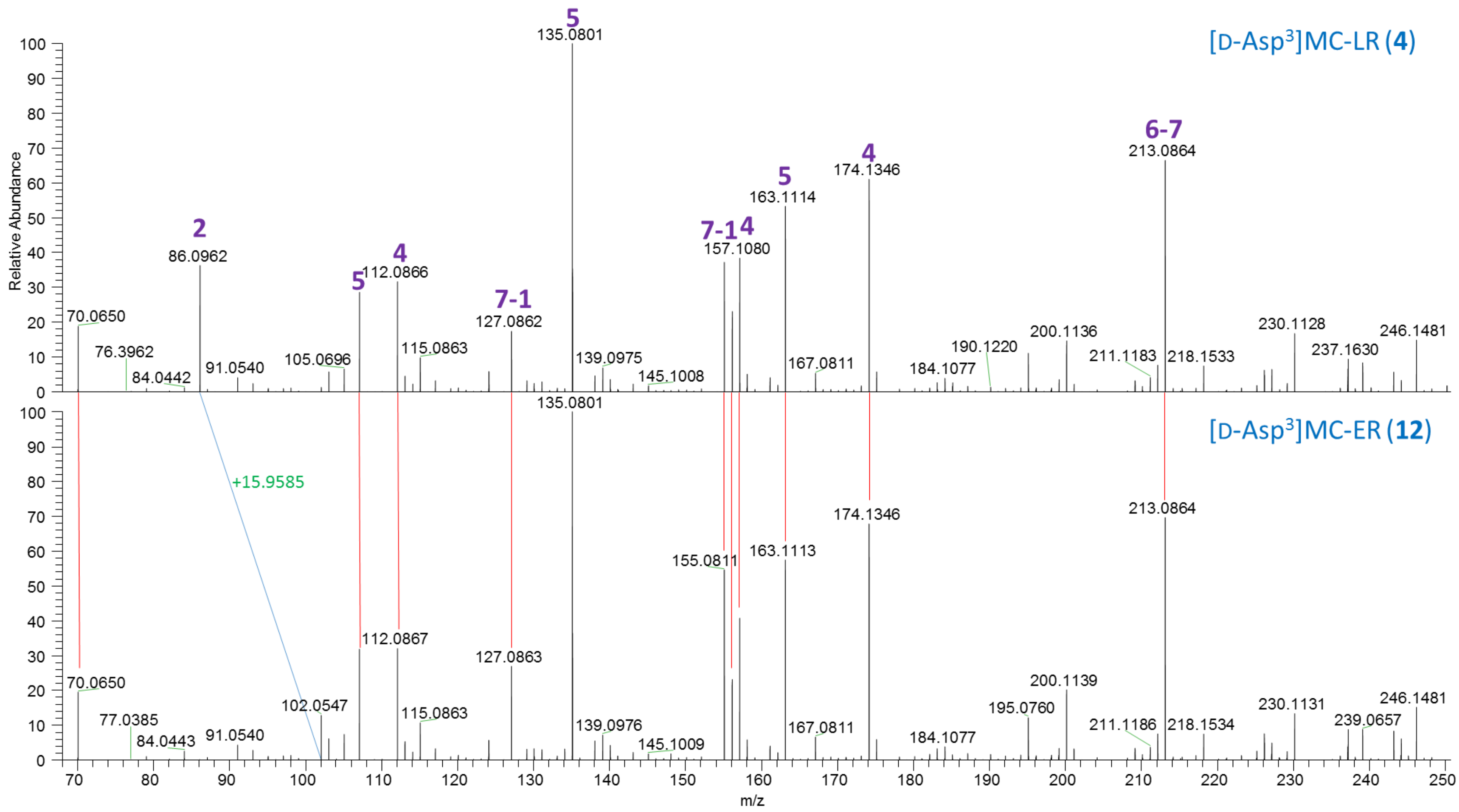

Figure S22. Expansion (*m/z* 68–250) of the LC–HRMS/MS PRM spectra (method B) of $[M + H]^+$ of [D-Asp^3]MC-LR (**4**) at *m/z* 981.5 (top), and of [D-Asp^3]MC-ER (**12**) at *m/z* 997.5 (bottom). Blue lines join selected peaks that differ by *m/z* +15.9595, which is the exact mass difference between **4** and **12**, and between leucine and glutamic acid. The bold purple numbers indicate the amino acid residue numbers of the amino acids attributed to selected product ions based on Yilmaz et al.[1] The results show that **4** and **12** differ by 15.9595 Da in amino acid-2.

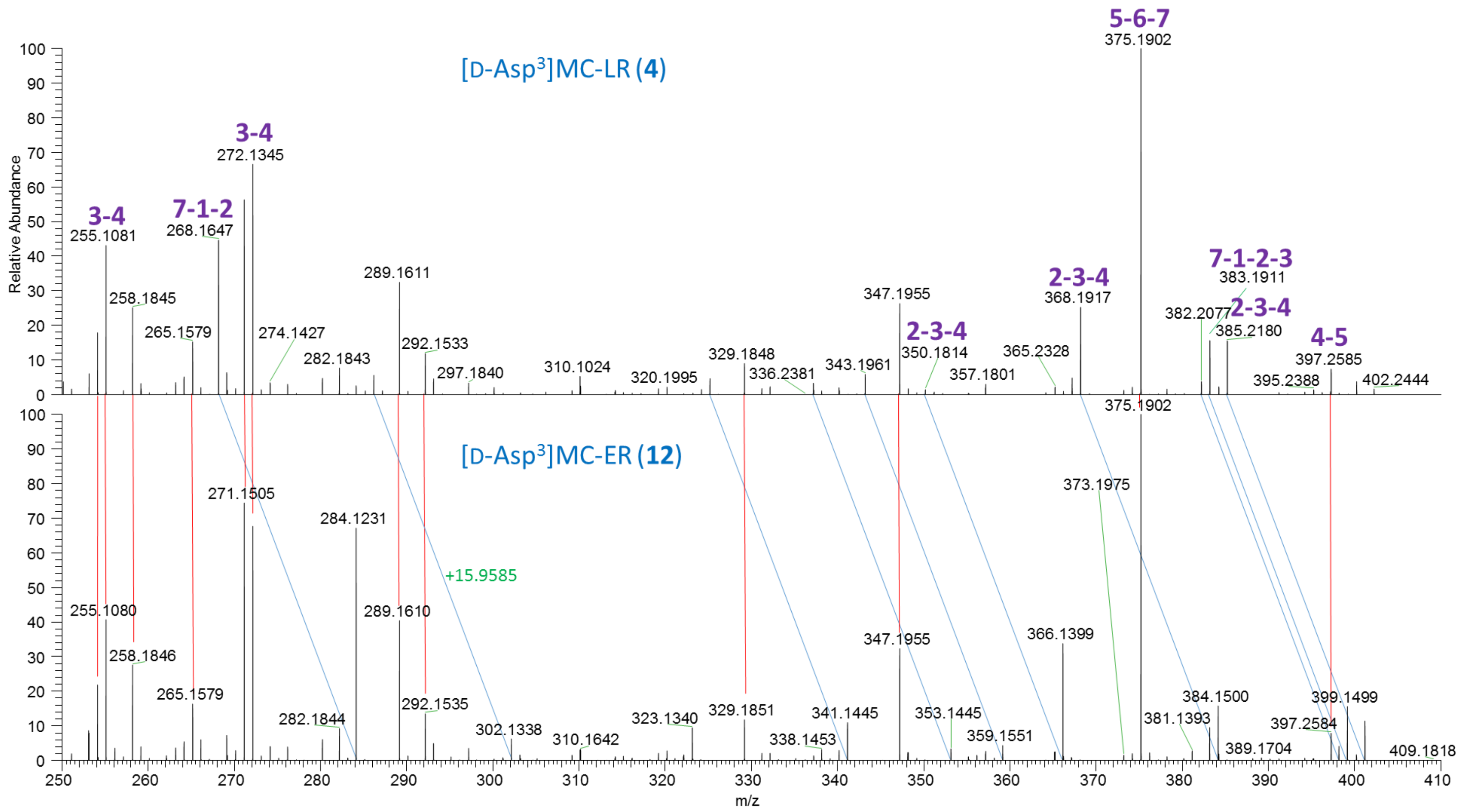

Figure S23. Expansion (*m/z* 250–410) of the LC–HRMS/MS PRM spectra (method B) of $[M + H]^+$ of [D-Asp^3]MC-LR (**4**) at *m/z* 981.5 (top), and of [D-Asp^3]MC-ER (**12**) at *m/z* 997.5 (bottom). Blue lines join selected peaks that differ by *m/z* +15.9595, which is the exact mass difference between **4** and **12** (and between leucine and glutamic acid). The bold purple numbers indicate the amino acid residue numbers of the amino acids attributed to selected product ions based on Yilmaz et al. (2019). The results show that **4** and **12** differ by 15.9595 Da in amino acid-2.

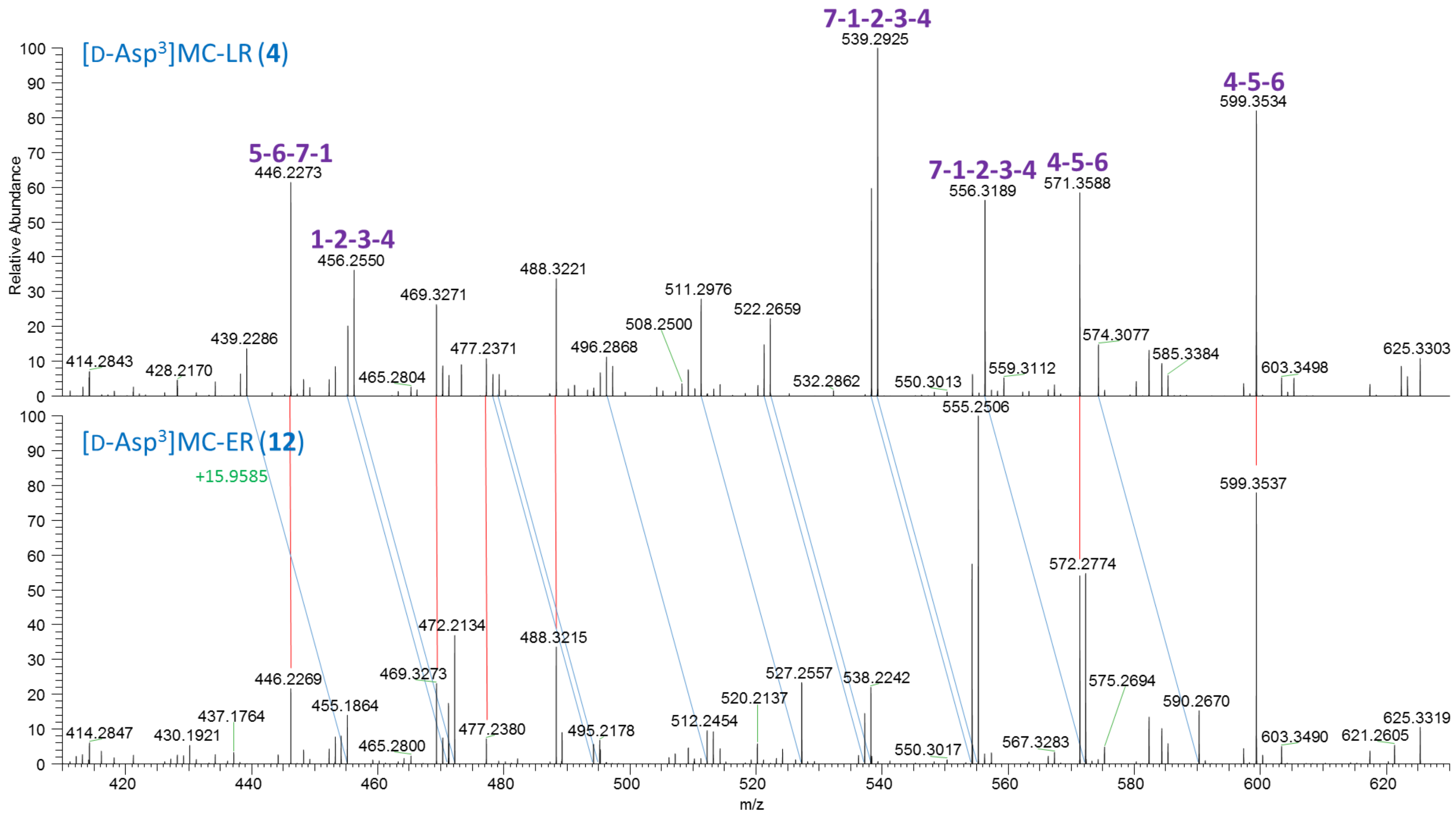

Figure S24. Expansion (*m*/*z* 410–630) of the LC–HRMS/MS PRM spectra (method B) of [M + H]$^+$ of [D-Asp^3]MC-LR (**4**) at *m*/*z* 981.5 (top), and of [D-Asp^3]MC-ER (**12**) at *m*/*z* 997.5 (bottom). Blue lines join selected peaks that differ by *m*/*z* +15.9595, which is the exact mass difference between **4** and **12** (and between leucine and glutamic acid). The bold purple numbers indicate the amino acid residue numbers of the amino acids attributed to selected product ions based on Yilmaz et al. (2019). The results show that **4** and **12** differ by 15.9595 Da in amino acid-2.

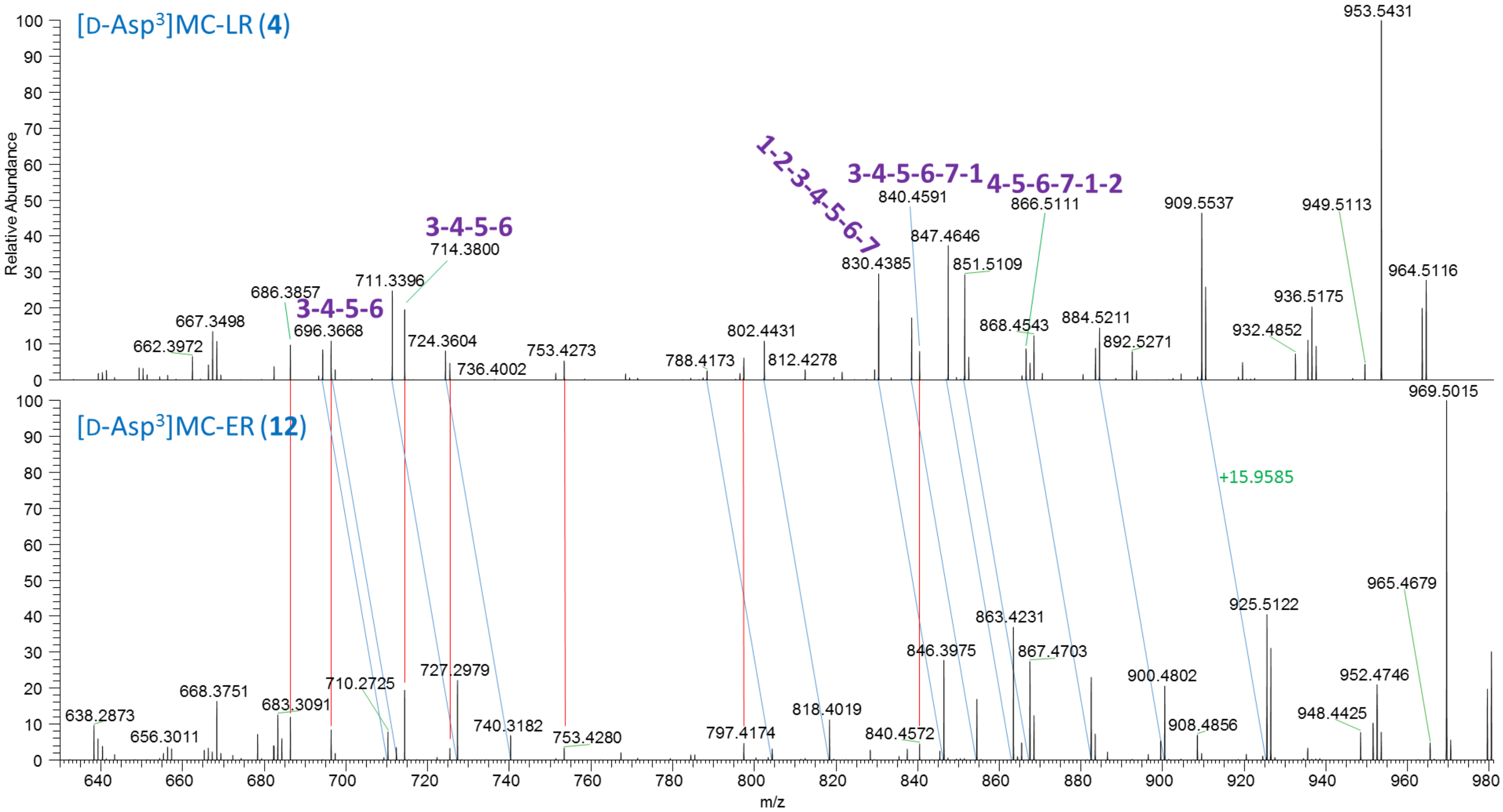

Figure S25. Expansion (*m/z* 630–981) of the LC–HRMS/MS PRM spectra (method B) of $[M + H]^+$ of [D-Asp[3]]MC-LR (**4**) at *m/z* 981.5 (top), and of [D-Asp[3]]MC-ER (**12**) at *m/z* 997.5 (bottom). Blue lines join selected peaks that differ by *m/z* +15.9595, which is the exact mass difference between **4** and **12** (and between leucine and glutamic acid). The bold purple numbers indicate the amino acid residue numbers of the amino acids attributed to selected product ions based on Yilmaz et al. (2019). T
he results show that **4** and **12** differ by 15.9595 Da in amino acid-2.

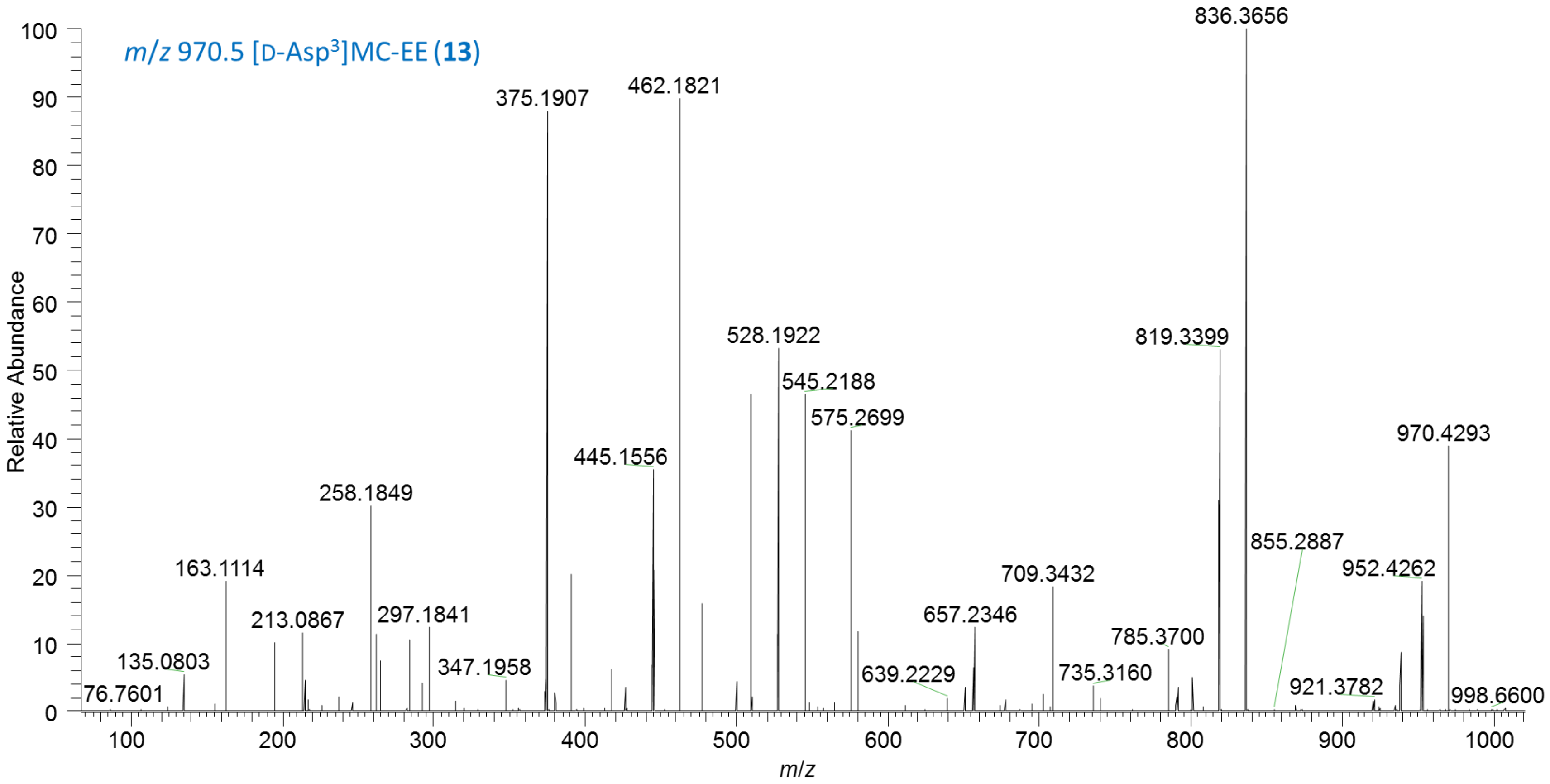

Figure S26. LC–HRMS/MS PRM spectrum (method B) of $[M + H]^+$ of [D-Asp^3]MC-EE (**13**) at *m/z* 970.5.

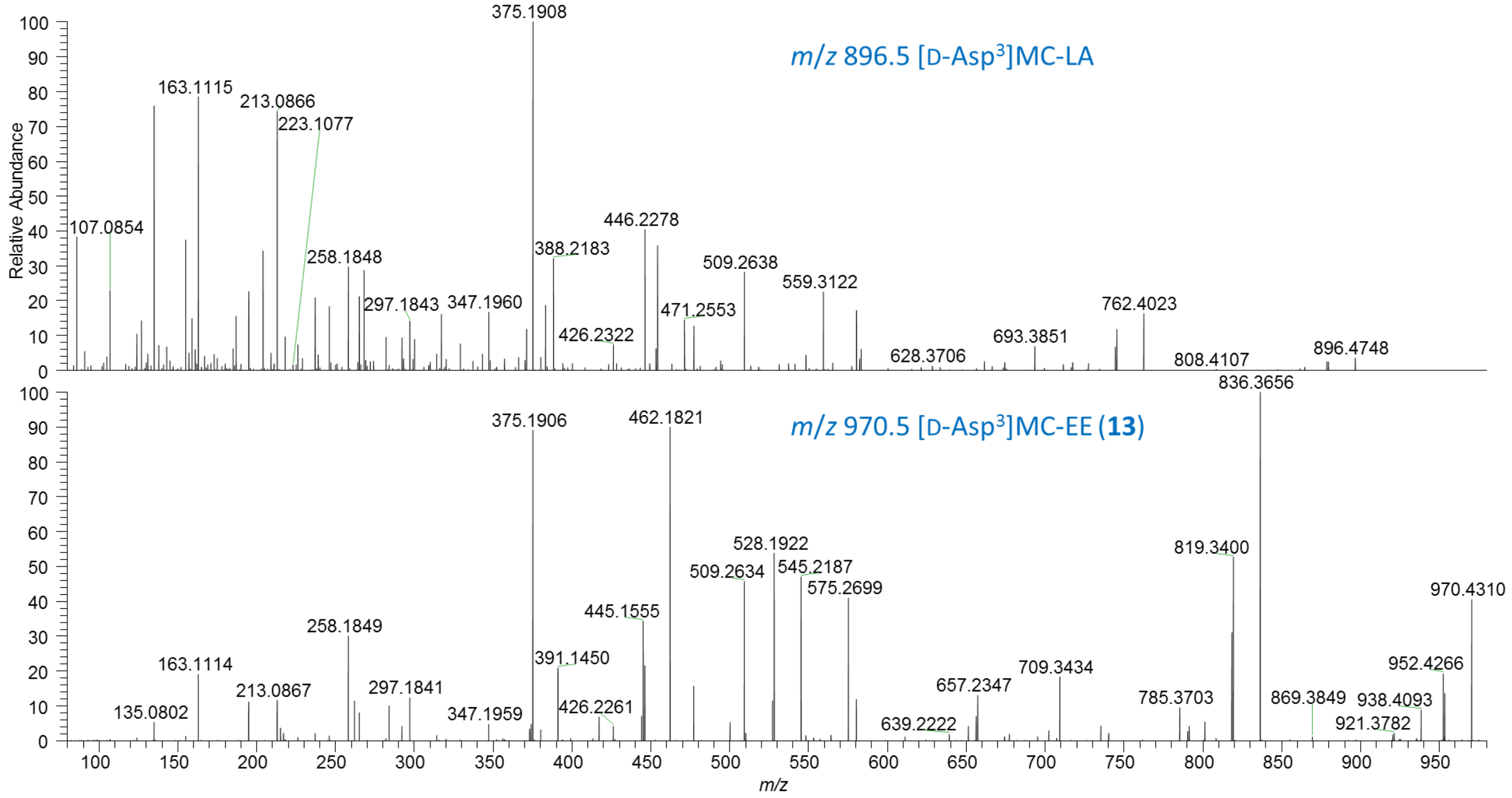

Figure S27. LC–HRMS/MS PRM spectra (method B) of $[M + H]^+$ of [D-Asp^3]MC-LA at *m*/*z* 896.5 (top), and of [D-Asp^3]MC-EE (**13**) at *m*/*z* 970.5 (bottom).

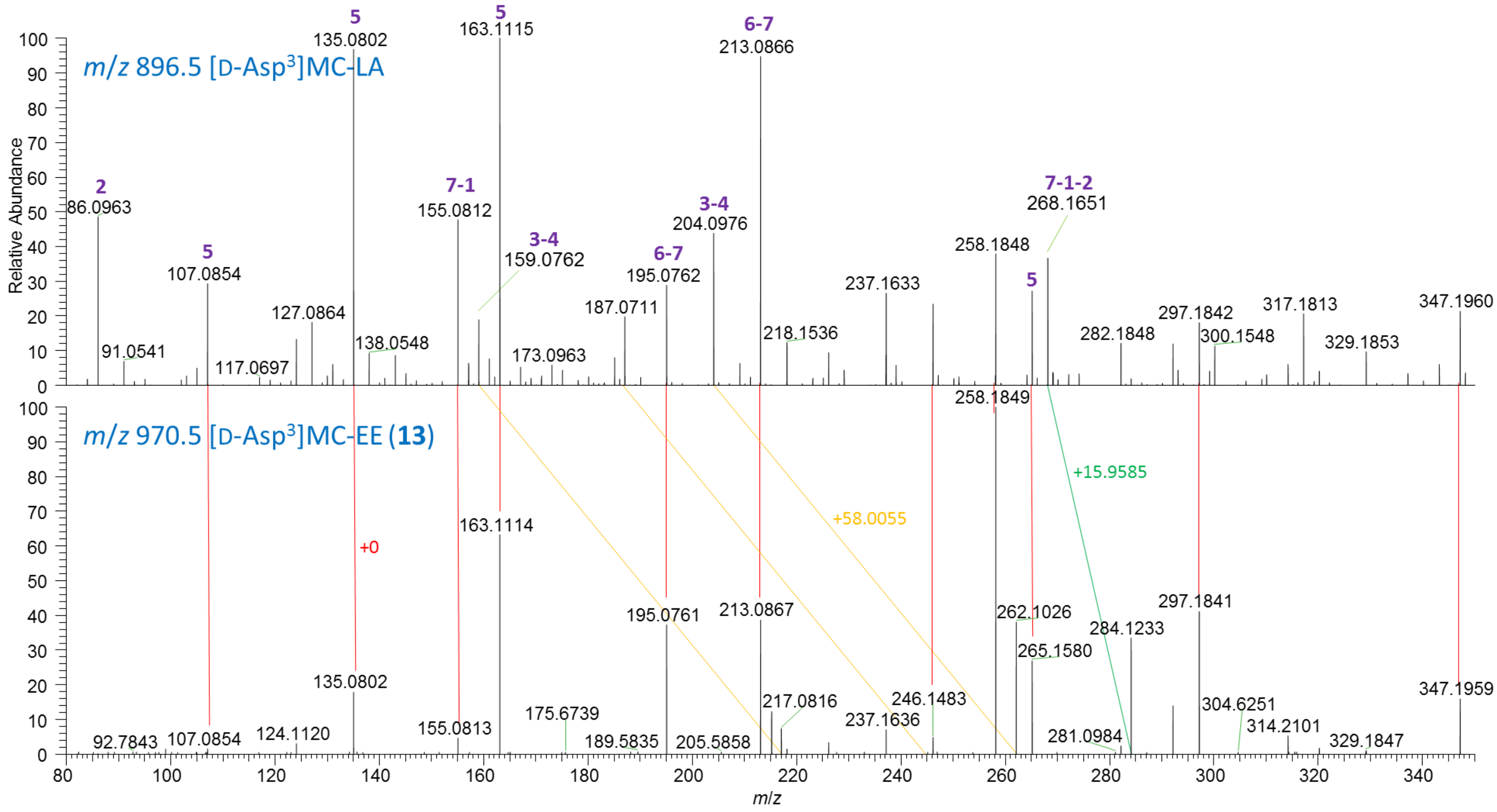

Figure S28. Expansion (*m/z* 80–350) of the LC–HRMS/MS PRM spectra (method B) of $[M + H]^+$ of [D-Asp^3]MC-LA at *m/z* 896.5 (top), and of [D-Asp^3]MC-EE (**13**) at *m/z* 970.5 (bottom). Blue lines join peaks differing by *m/z* +73.9640 (the exact mass difference between [D-Asp^3]MC-LA and **13**) and contain both amino acid-2 and -4; orange lines join peaks differing by *m/z* +58.0055 (the difference in exact mass between Ala and Glu) and contain amino acid-4 but not -2; green lines join peaks differing by *m/z* +15.9585 (the difference in exact mass between Leu and Glu) and contain amino acid-2 but not -4; red lines join peaks that do not differ between the two compounds, and thus contain neither amino acid-2 nor -4. Bold purple numbers indicate the amino acid residue numbers of the amino acids attributed to selected product ions based on LeBlanc et al.[2] The results show that **13** and [D-Asp^3]MC-LA differ by +15.9585 Da in amino acid-2 and by +58.0055 in amino acid-4.

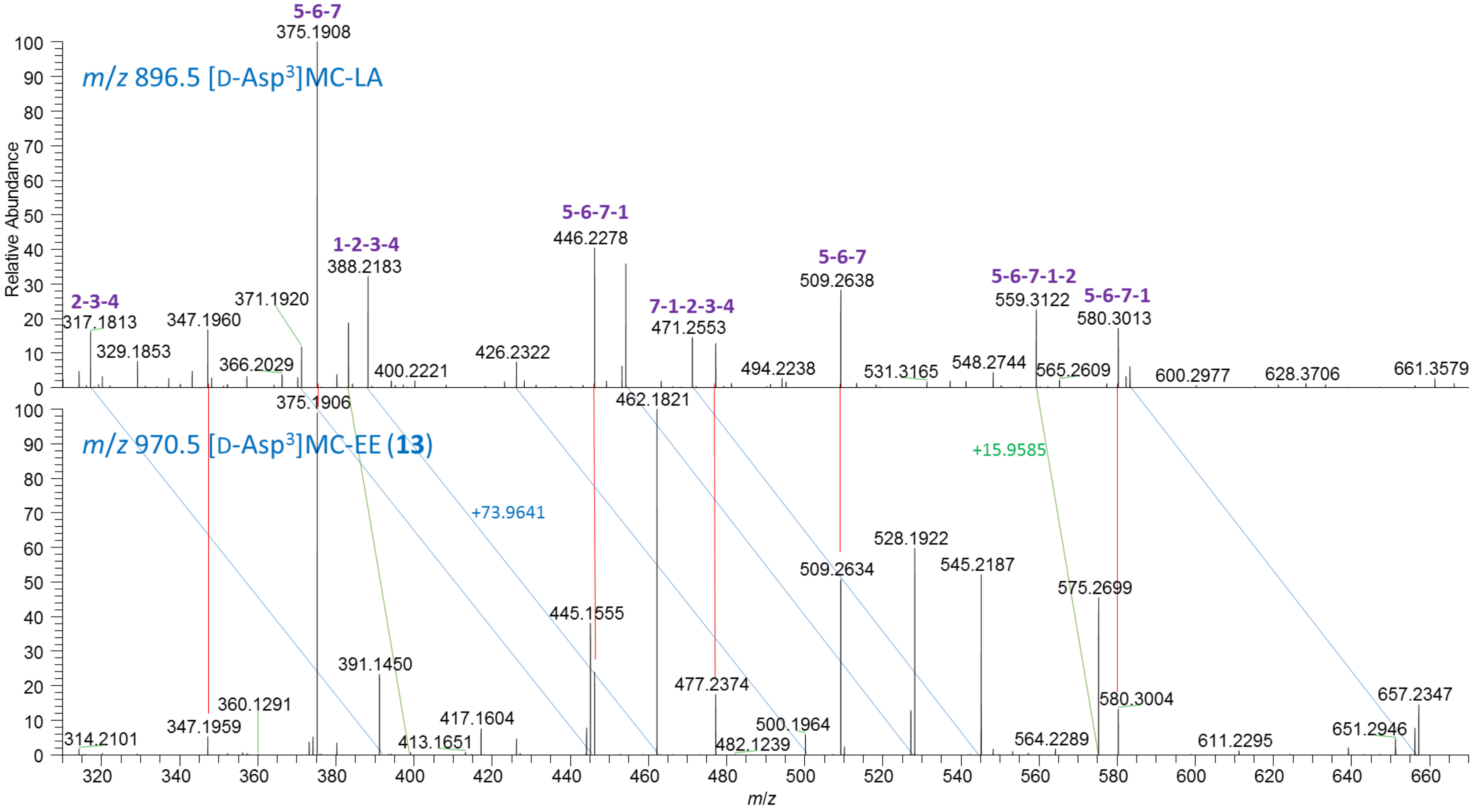

Figure S29. Expansion (*m*/*z* 330–670) of the LC–HRMS/MS PRM spectra (method B) of $[M + H]^+$ of [D-Asp^3]MC-LA at *m*/*z* 896.5 (top), and of [D-Asp^3]MC-EE (**13**) at *m*/*z* 970.5 (bottom). Blue lines join peaks differing by *m*/*z* +73.9640 (the exact mass difference between [D-Asp^3]MC-LA and **13**) and contain both amino acid-2 and -4; orange lines join peaks differing by *m*/*z* +58.0055 (the difference in exact mass between Ala and Glu) and contain amino acid-4 but not -2; green lines join peaks differing by *m*/*z* +15.9585 (the difference in exact mass between Leu and Glu) and contain amino acid-2 but not -4; red lines join peaks that do not differ between the two compounds, and thus contain neither amino acid-2 nor -4. Bold purple numbers indicate the amino acid residue numbers of the amino acids attributed to selected product ions based on LeBlanc et al.[2] The results show that **13** and [D-Asp^3]MC-LA differ by +15.9585 Da in amino acid-2 and by +58.0055 in amino acid-4.

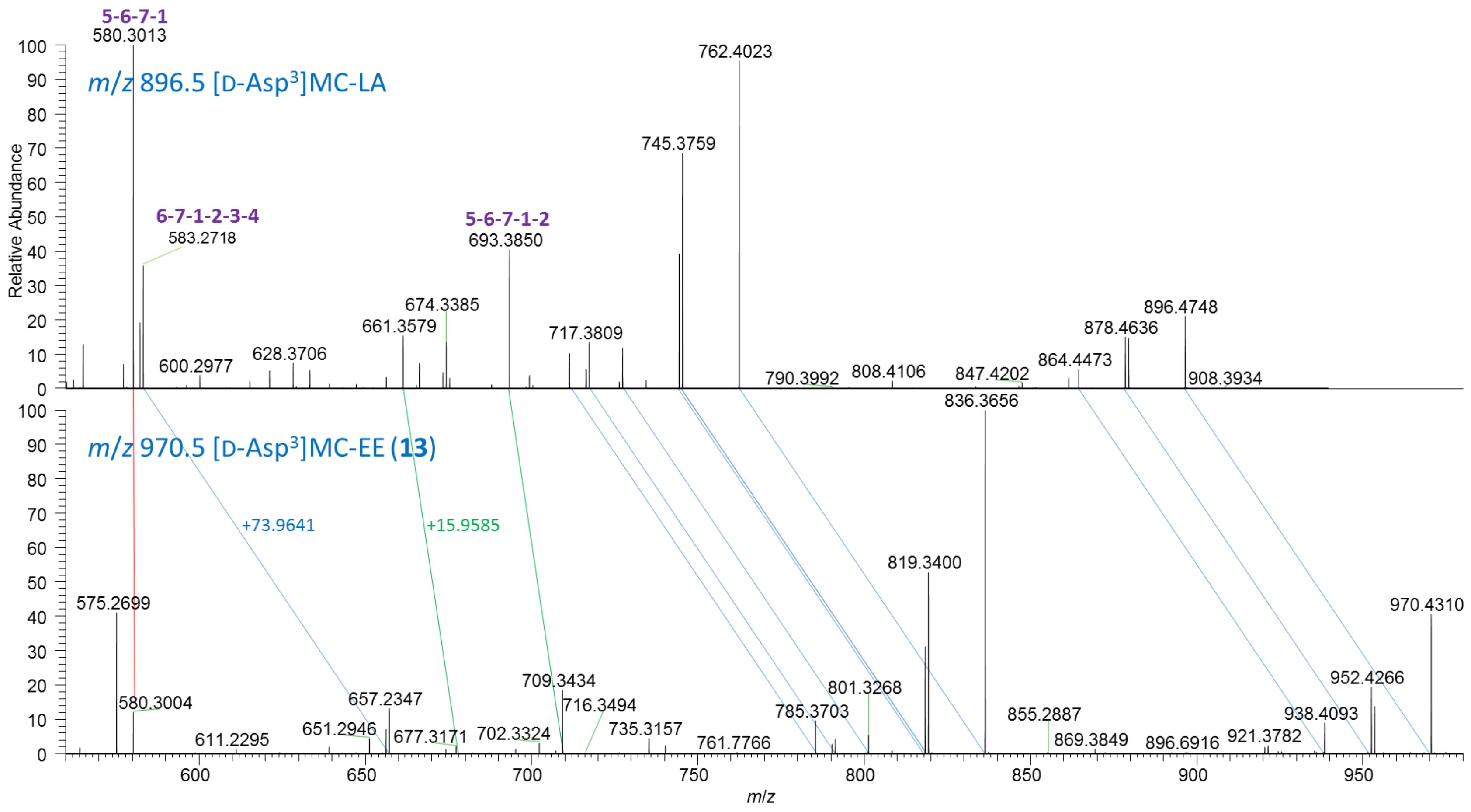

Figure S30. Expansion (*m/z* 560–980) of the LC–HRMS/MS PRM spectra (method B) of $[M + H]^+$ of [D-Asp^3]MC-LA at *m/z* 896.5 (top), and of [D-Asp^3]MC-EE (**13**) at *m/z* 970.5 (bottom). Blue lines join peaks differing by *m/z* +73.9640 (the exact mass difference between [D-Asp^3]MC-LA and **13**) and contain both amino acid-2 and -4; orange lines join peaks differing by *m/z* +58.0055 (the difference in exact mass between Ala and Glu) and contain amino acid-4 but not -2; green lines join peaks differing by *m/z* +15.9585 (the difference in exact mass between Leu and Glu) and contain amino acid-2 but not -4; red lines join peaks that do not differ between the two compounds, and thus contain neither amino acid-2 nor -4. Bold purple numbers indicate the amino acid residue numbers of the amino acids attributed to selected product ions based on LeBlanc et al.[2] The results show that **13** and [D-Asp^3]MC-LA differ by +15.9585 Da in amino acid-2 and by +58.0055 in amino acid-4.

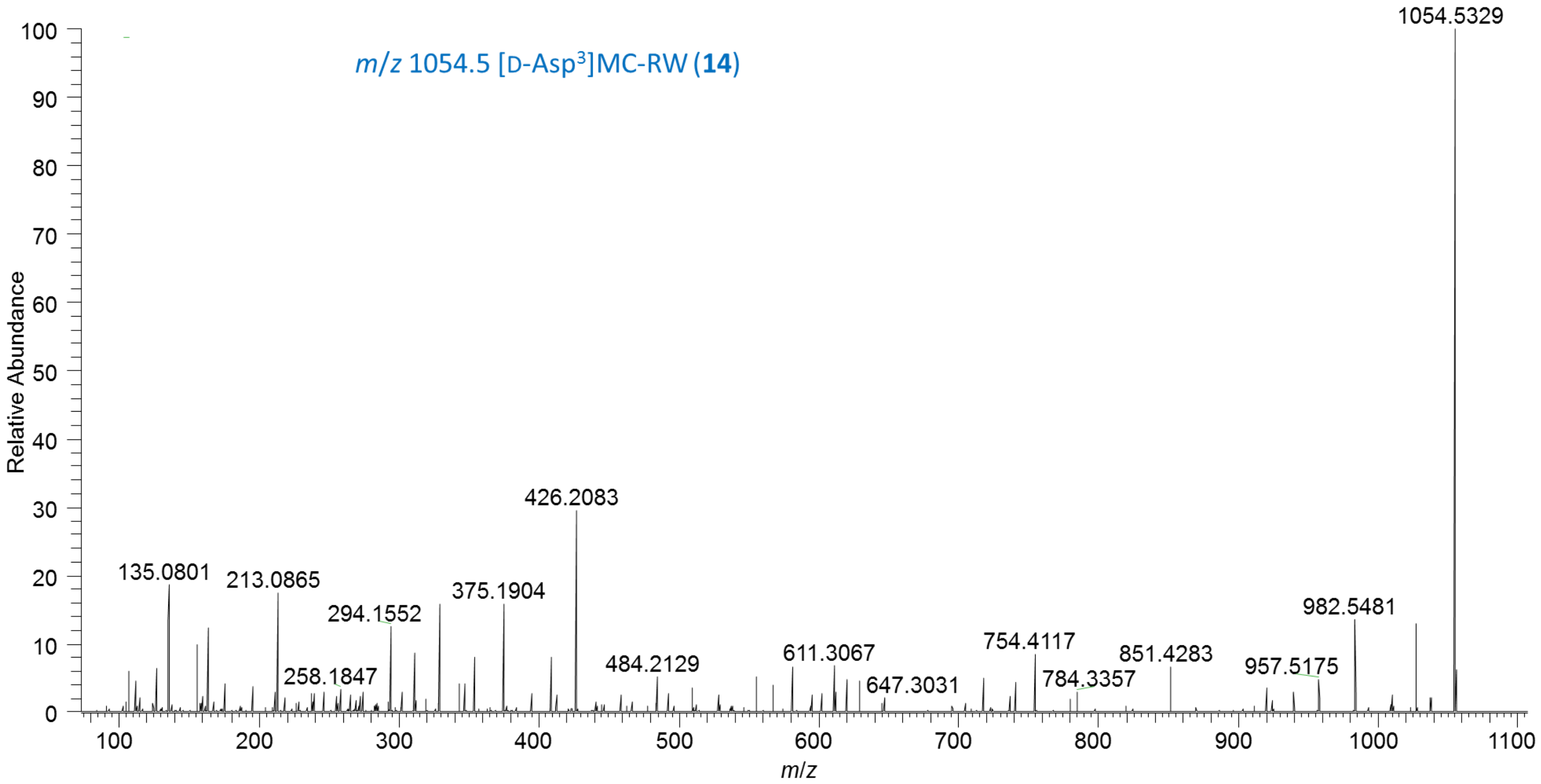

Figure S31. LC–HRMS/MS PRM spectrum (method B) of [M + H]$^+$ of [D-Asp^3]MC-RW (**14**) at *m*/*z* 1054.5.

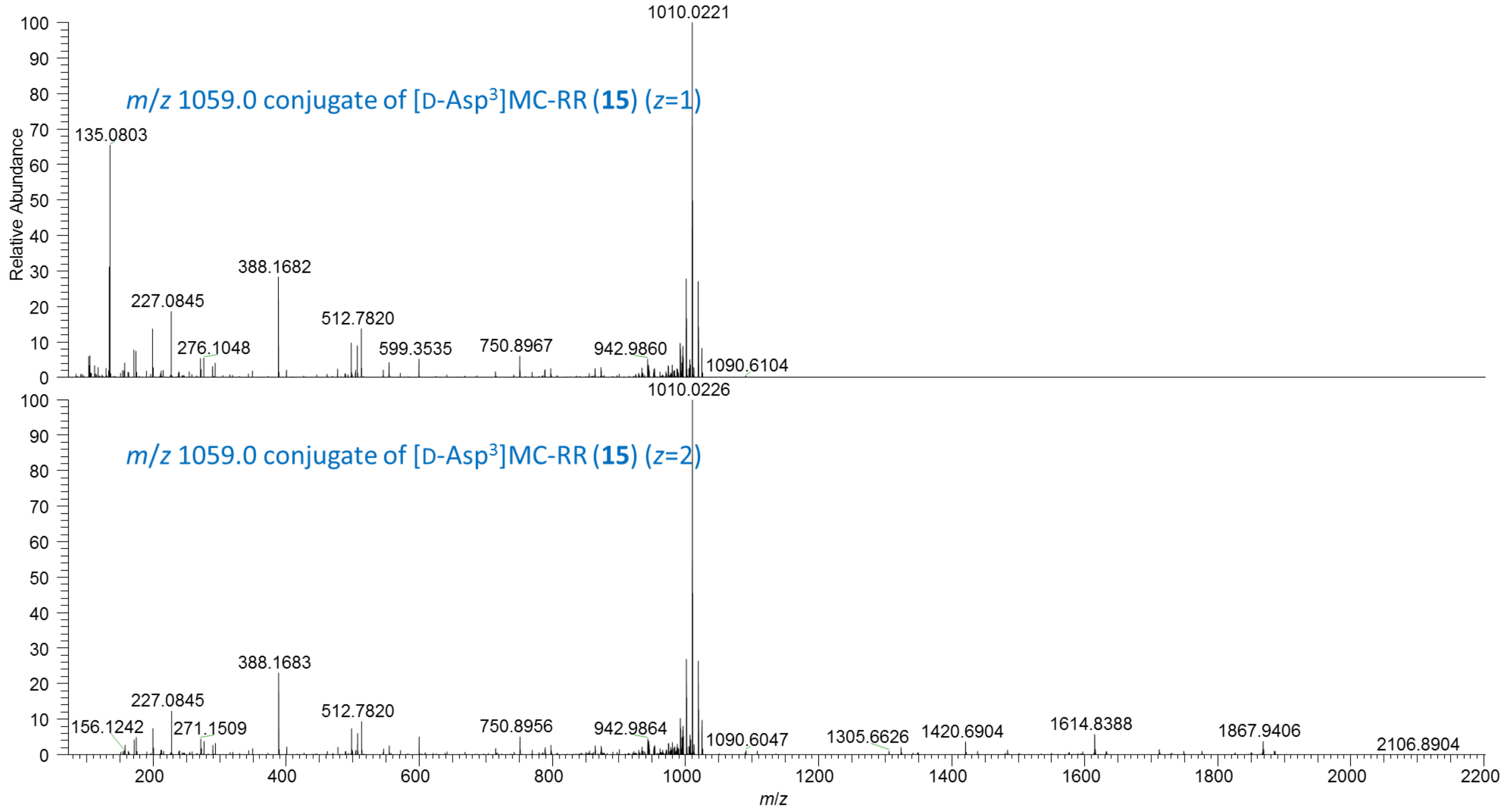

Figure S32. LC–HRMS/MS PRM spectra (method B) of $[M + 2H]^{2+}$ of the sulfide conjugate of [D-Asp^3]MC-RR (**15**) at *m*/*z* 1059.0 recorded with setting *z* = 1 (top) leading to scanning from *m*/*z* 73–1100, and recorded with setting *z* = 2 leading to scanning from *m*/*z* 145–2180 (bottom).

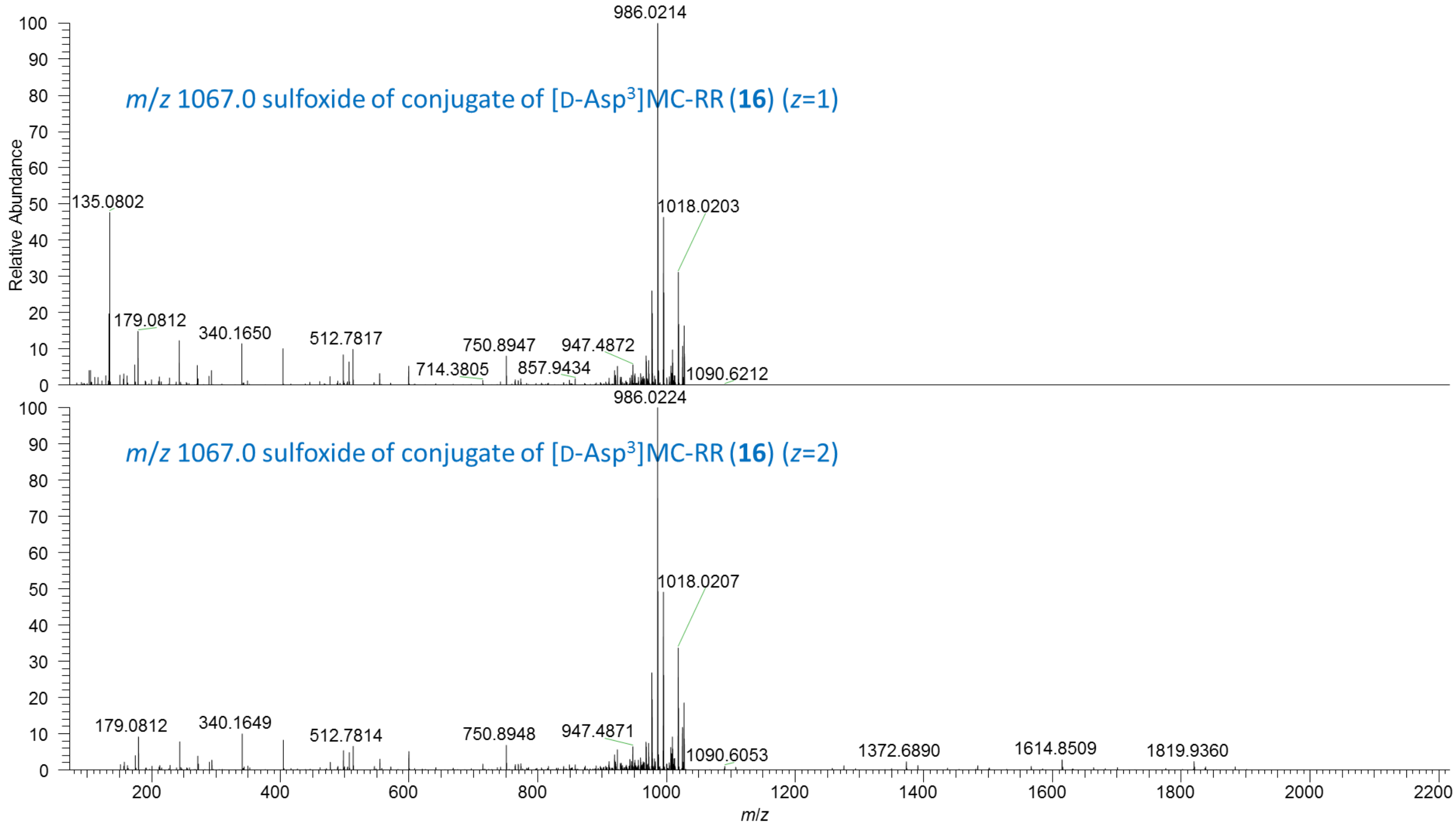

Figure S33. LC–HRMS/MS PRM spectra (method B) of $[M + 2H]^{2+}$ of the sulfoxide conjugate of [D-Asp^3]MC-RR (**16**) at *m*/*z* 1067.0 recorded with setting *z* = 1 (top) leading to scanning from *m*/*z* 73–1105, and recorded with setting *z* = 2 leading to scanning from *m*/*z* 146–2195 (bottom).

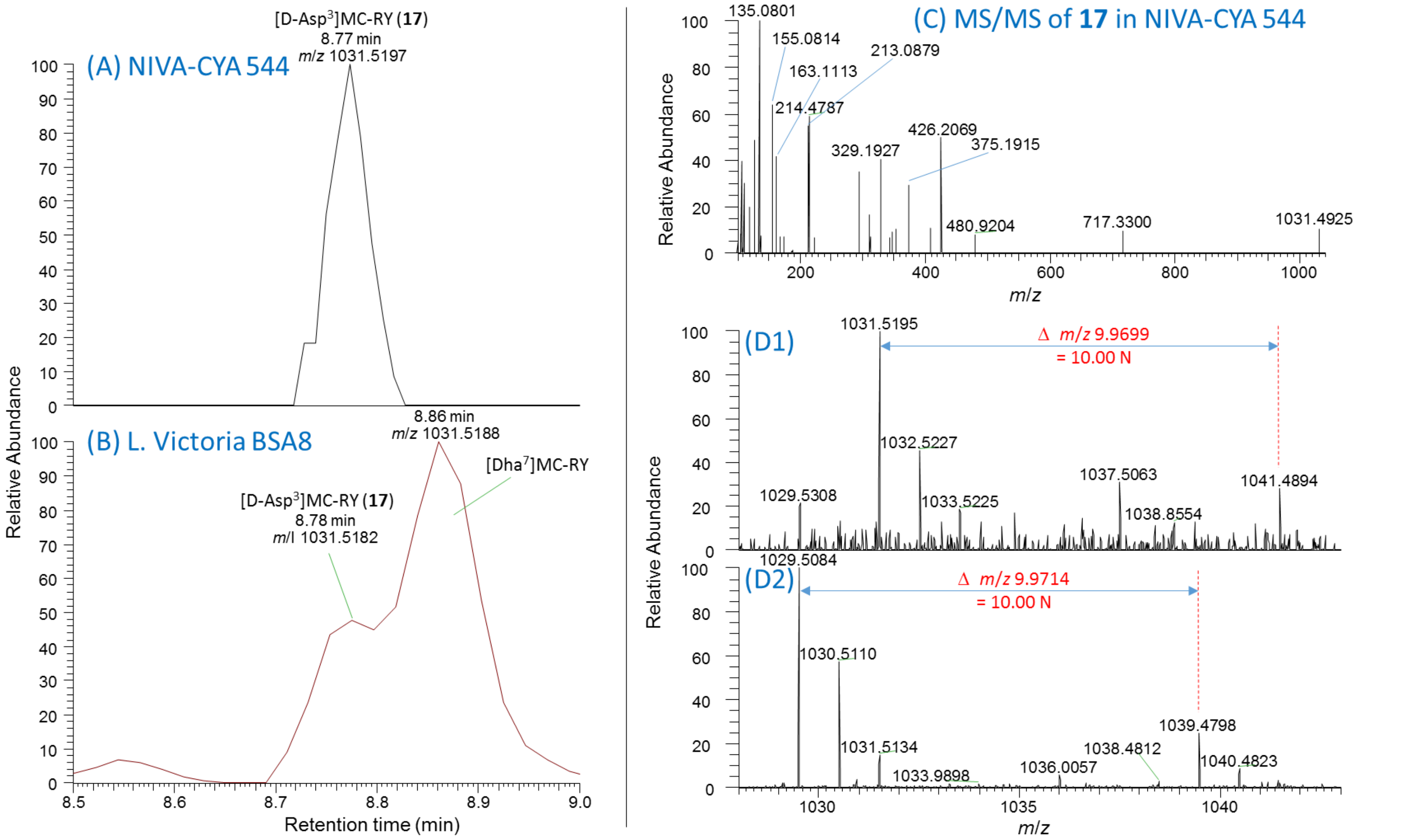

Figure S34. Extracted positive ion LC−MS chromatograms (method B) at *m/z* 1031.5197 an extract of: A, NIVA-CYA 544, and; B, Lake Victoria bloom sample BSA8,[3] showing co-elution of **17** with a sample containing [D-Asp^3]MC-RY (**17**). Panel C shows a weak positive ion mode MS/MS spectrum of **17** from NIVA-CYA 544 showing characteristic product ions for an [D-Asp^3]MC-RZ congener (*cf*. Figures S31, S35–37). Panels D1 and D2 show positive and negative ion full scan MS spectra of **17** in a mixture of extracts from an unlabelled culture of NIVA-CYA 544 and a culture grown in medium containing 98% ^{15}N, showing that **17** contains 10 nitrogen atoms (see also Figure S51 for application of the NRC formula calculator to this data).

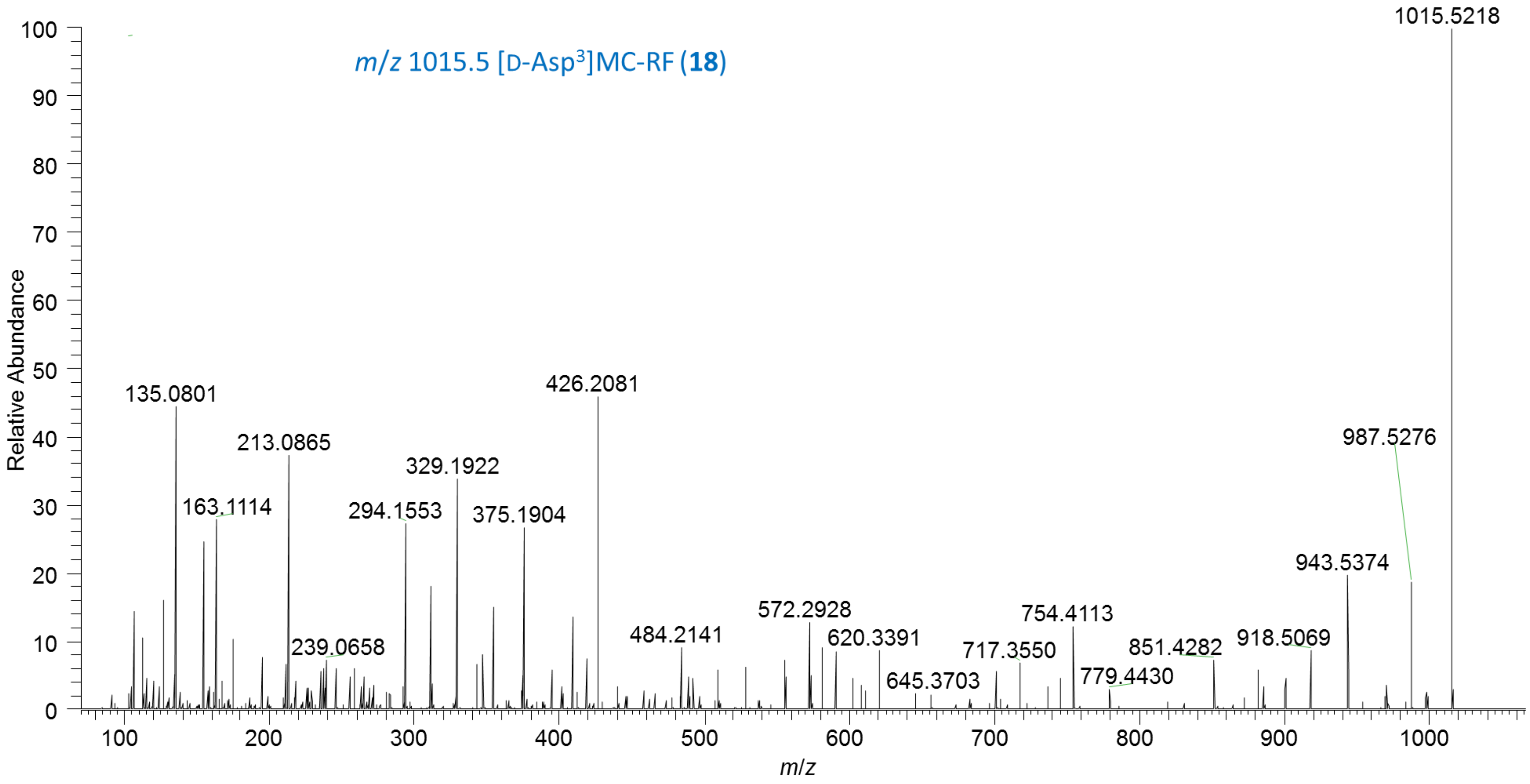

Figure S35. LC–HRMS/MS PRM spectrum (method B) of $[M + H]^+$ of [D-Asp[3]]MC-RF (**18**) at *m*/*z* 1015.5.

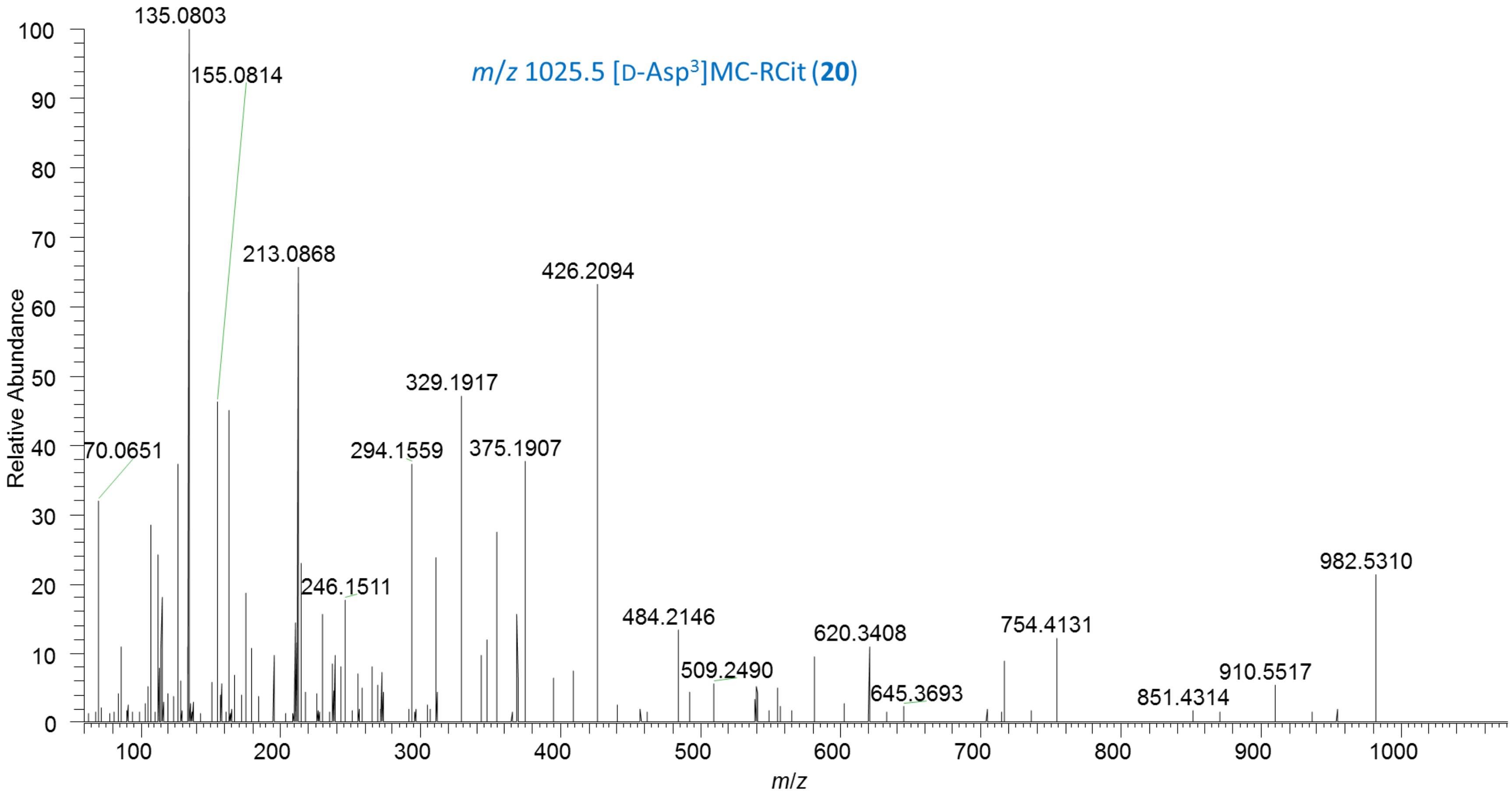

Figure S36. LC–HRMS/MS PRM spectrum (method B) of $[M + H]^+$ of [D-Asp[3]]MC-RCit (**20**) at *m/z* 1025.5.

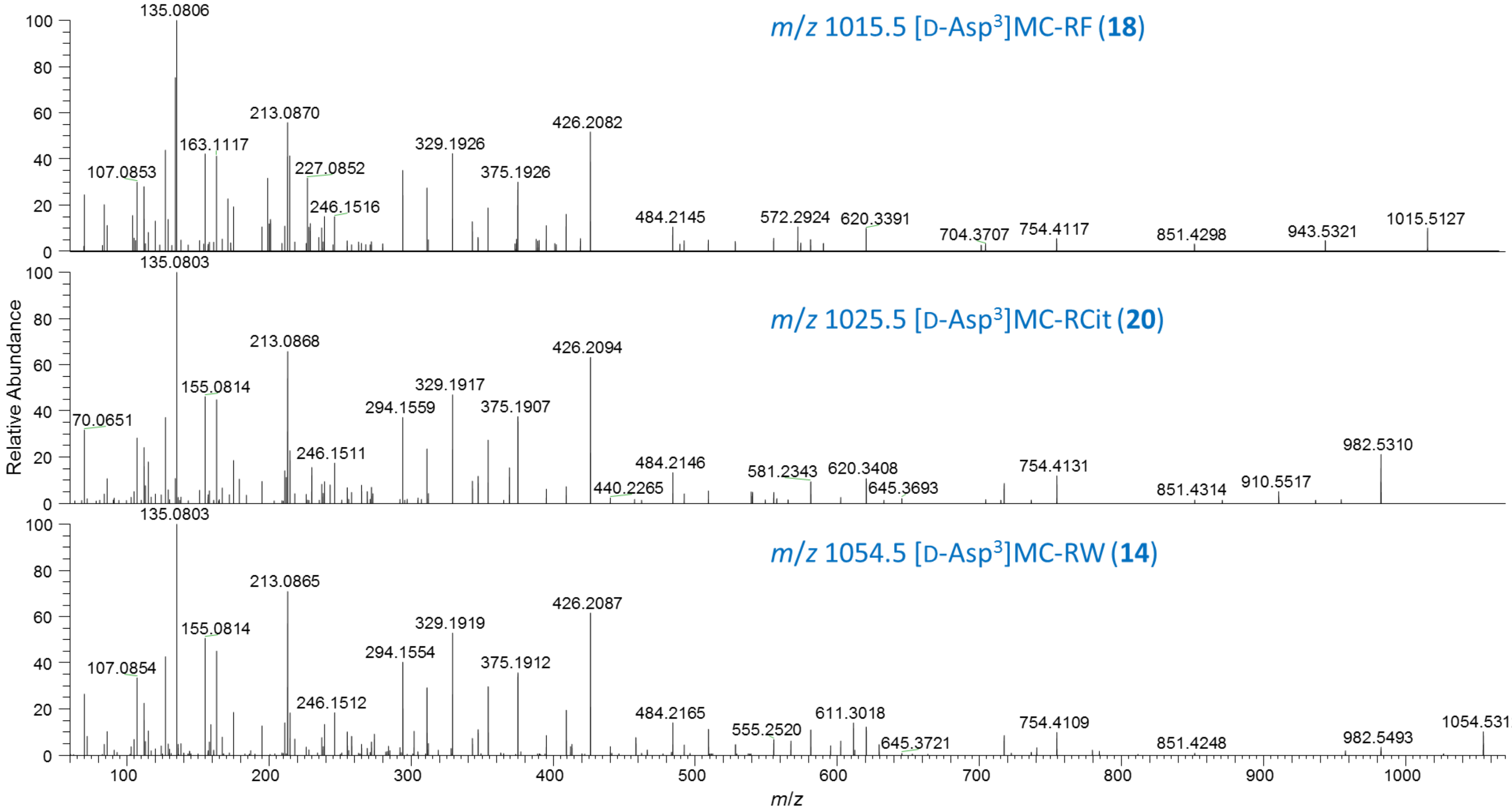

Figure S37. LC–HRMS/MS PRM spectra (method B) of $[M + H]^+$ of [D-Asp^3]MC-RF (**18**) at *m/z* 1015.5 (top), [D-Asp^3]MC-RCit (**20**) at *m/z* 1025.5 (middle), and [D-Asp^3]MC-RW (**14**) at *m/z* 1054.5 (bottom). Note the prominent product ions at *m/z* 375.1915 (Adda^5–D-Glu^6–Mdha^7 minus $C_9H_{10}O$), and 426.2096 (Dha^7–D-Ala^1–Arg^2-D-Masp^3) that are characteristic 3-desmethylated MCs containing Arg at position-2 (i.e. [D-Asp^3]MC-RZ) (*cf* also spectra of [D-Asp^3]MC-RR (**1**)).

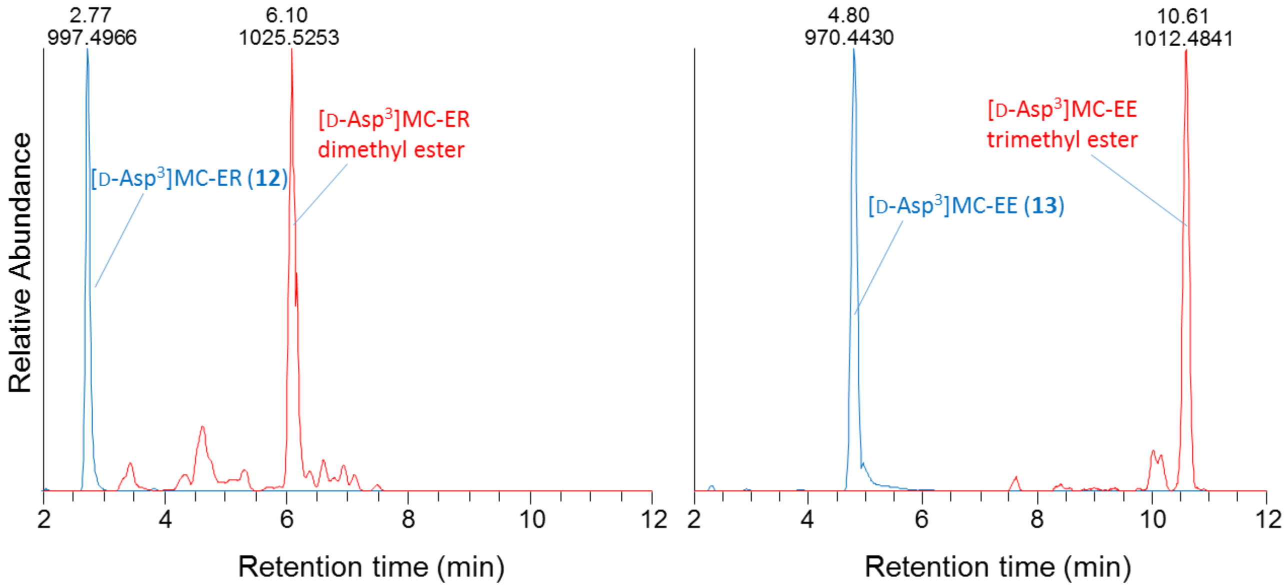

Figure S38. Extracted ion LC–HRMS chromatograms (method A) of an extract of NIVA-CYA 544 before (blue) and after (red) esterification with diazomethane. Left, extracted for *m*/*z* corresponding to $[M + H]^+$ for [D- Asp^3]MC-ER (**12**) and its mono-, di-, and tri-methyl esters; right, extracted for *m*/*z* corresponding to $[M + H]^+$ for [D-Asp^3]MC-EE (**13**) and its mono-, di-, and tri-methyl esters. Results confirmed the presence of one extra carboxylic acid group in **12**, and two extra carboxylic acid groups in **13**, relative to [D-Asp^3]MC-LR (**4**), which was converted almost completely to its mono-methyl ester in the same experiment. Earlier retention times (compared to those ones reported in Table 1 and Table S1) result from use of an older column for monitoring this reaction.

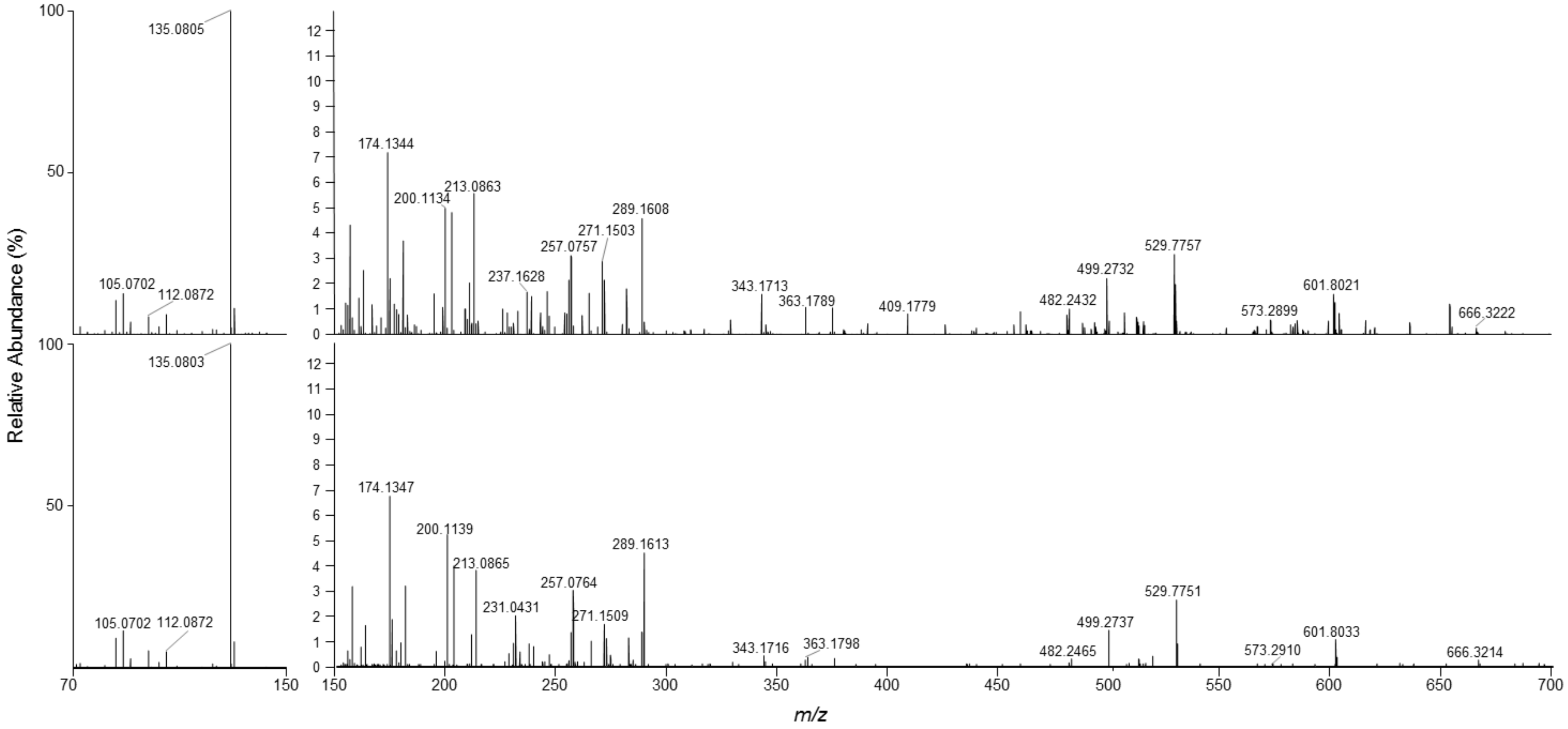

Figure S39. LC−HRMS/MS PRM spectra (method A) of $[M + 2H]^{2+}$ of putative GSH-conjugate of **1** (**19**) extracted at *m/z* 666.3. Top, in an extract of NIVA-CYA 544; bottom, in the product obtained from the reaction of a standard of [D-Asp^3]MC-RR (**1**) with glutathione (Figure S15). The mass range has been split into two segments with different vertical scales because of the dominance of the Adda-fragment (*m/z* 135.0804) in the spectra. The low mass range (m/z 70–150) is shown at full vertical scale (0–100%), whereas an expanded vertical scale (0–13%) was used in the range *m/z* 150–700 to allow visualization of the low-intensity fragments.

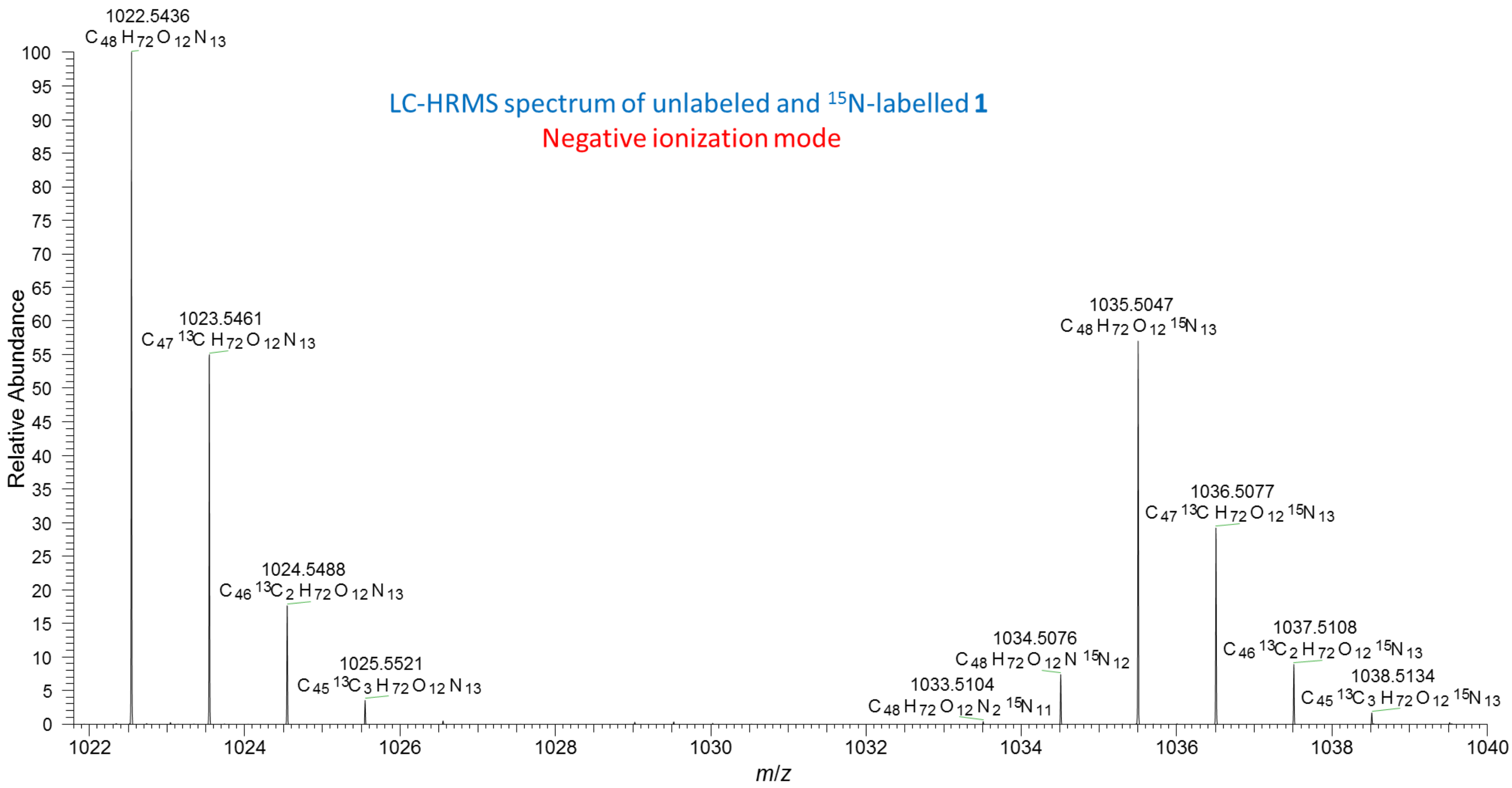

Figure 40. LC-HRMS (method B) spectrum of [D-Asp^3]MC-RR (**1**) in negative ionization mode ([M − H]$^-$) from a sample containing a mixture of an extract from an unlabelled culture of *P. prolifica* NIVA-CYA 544 and from the same culture grown for an extended period in ^{15}N-enriched (>98% ^{15}N) culture medium. Peaks are labelled with their elemental compositions, with all mass errors $\Delta \leq 1.0$ ppm. The measured level of ^{15}N-incorporation for the labelled **1** was 98% (see Figure 3). The *m/z* values and isotopomer peak intensities were used in the NRC Molecular Formula Calculator to obtain candidate elemental compositions for **1** (see Figure S42). Similar data was used with the NRC Molecular Formula Calculator to obtain candidate elemental compositions for other microcystins in the culture (see Figures S43–57).

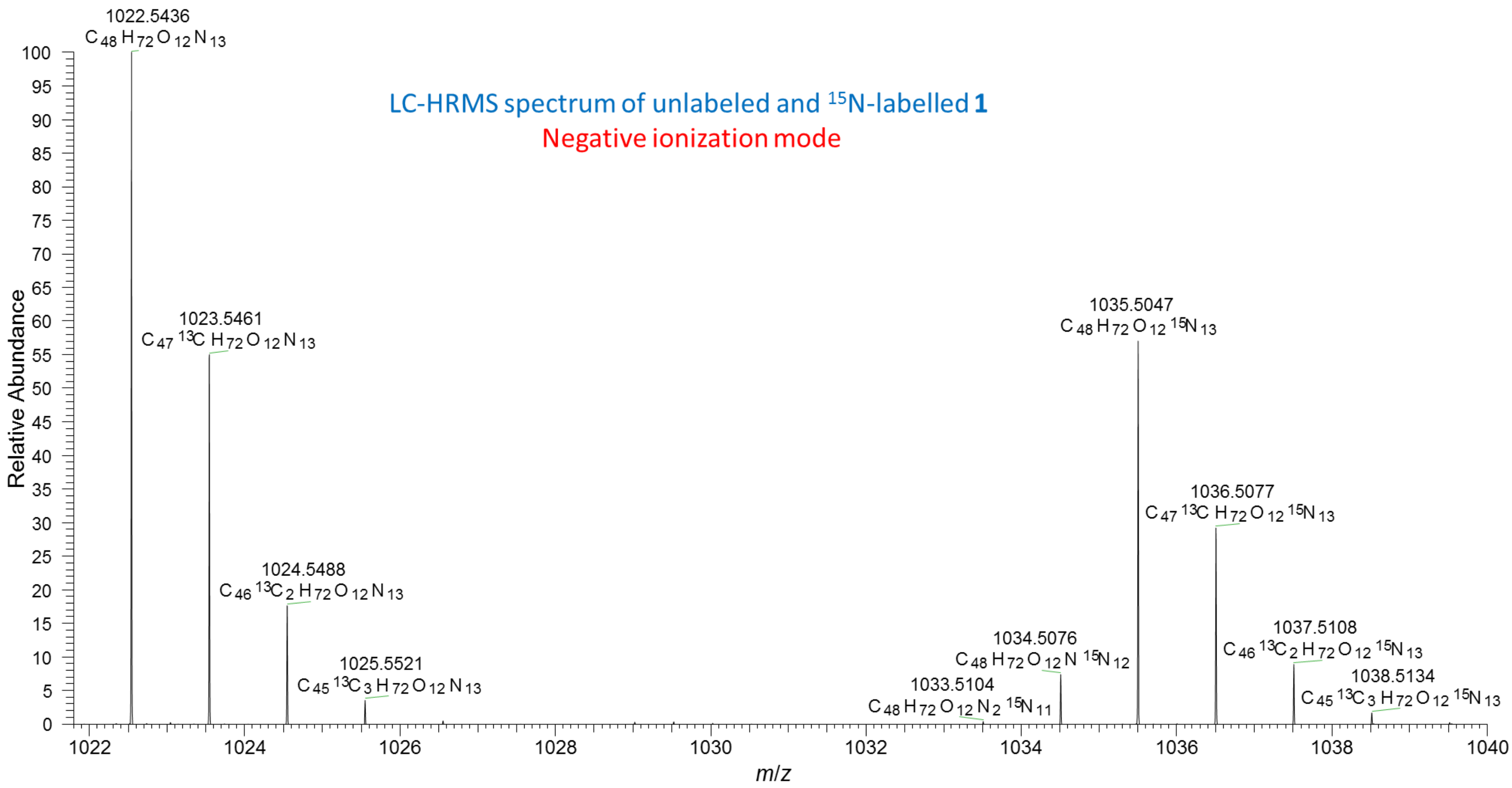

Figure 41. LC-HRMS (method B) spectrum of [D-Asp^3]MC-RR (**1**) in positive ionization mode ([M +2 H]$^{2+}$) from a sample containing a mixture of an extract from an unlabelled culture of *P. prolifica* NIVA-CYA 544 and from the same culture grown for an extended period in ^{15}N-enriched (>98% ^{15}N) culture medium. Peaks are labelled with their elemental compositions, with all mass errors $\Delta \leq 2.0$ ppm. The measured level of ^{15}N-incorporation for the labelled **1** was 98% (see Figure 3). The *m/z* values and isotopomer peak intensities were used in the NRC Molecular Formula Calculator to obtain candidate elemental compositions for **1** (see Figure S42). Similar data was used with the NRC Molecular Formula Calculator to obtain candidate elemental compositions for other microcystins in the culture (see Figures S43–57).

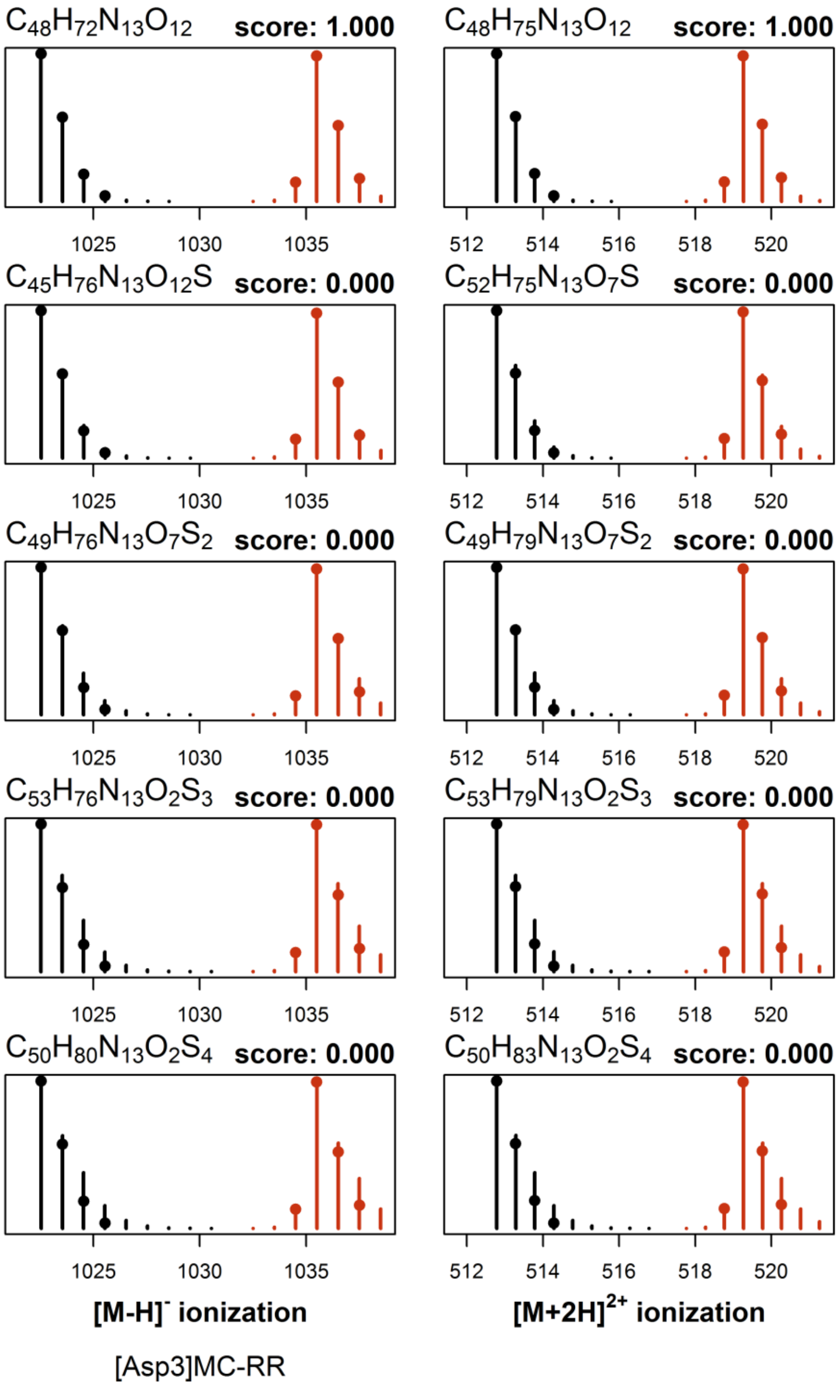

Figure S42. Most probable elemental compositions for [D-Asp[3]]MC-RR (**1**) based on full-scan LC–MS (method B) data for normalized ^{15}N-labelled (red) and natural abundance (black) cultures in negative (left) and positive (right) ionization modes using the NRC Molecular Formula Calculator. Candidate formulae and their scores are shown above the pairs of spectra in each panel, with the measured *m*/*z* and intensities indicated by the circles and the calculated values shown with vertical lines. For original data, see Figures S40 and S41.

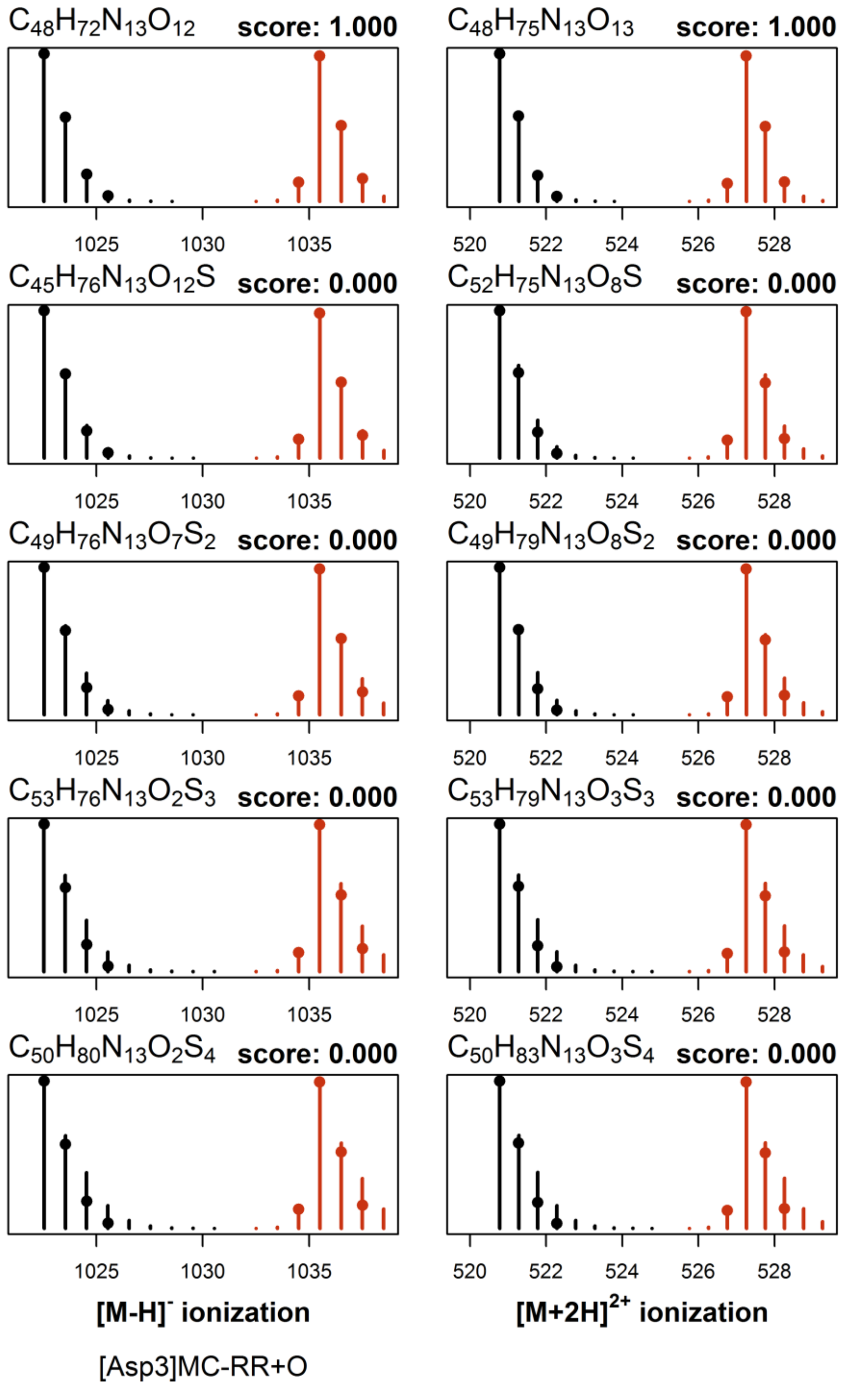

Figure S43. Most probable elemental compositions for oxidized [D-Asp^3]MC-RR (**1**-oxide) based on full-scan LC–MS (method B) data for normalized ^{15}N-labelled (red) and natural abundance (black) cultures in negative (left) and positive (right) ionization modes using the NRC Molecular Formula Calculator. Candidate formulae and their scores are shown above the pairs of spectra in each panel, with the measured *m*/*z* and intensities indicated by the circles and the calculated values shown with vertical lines.

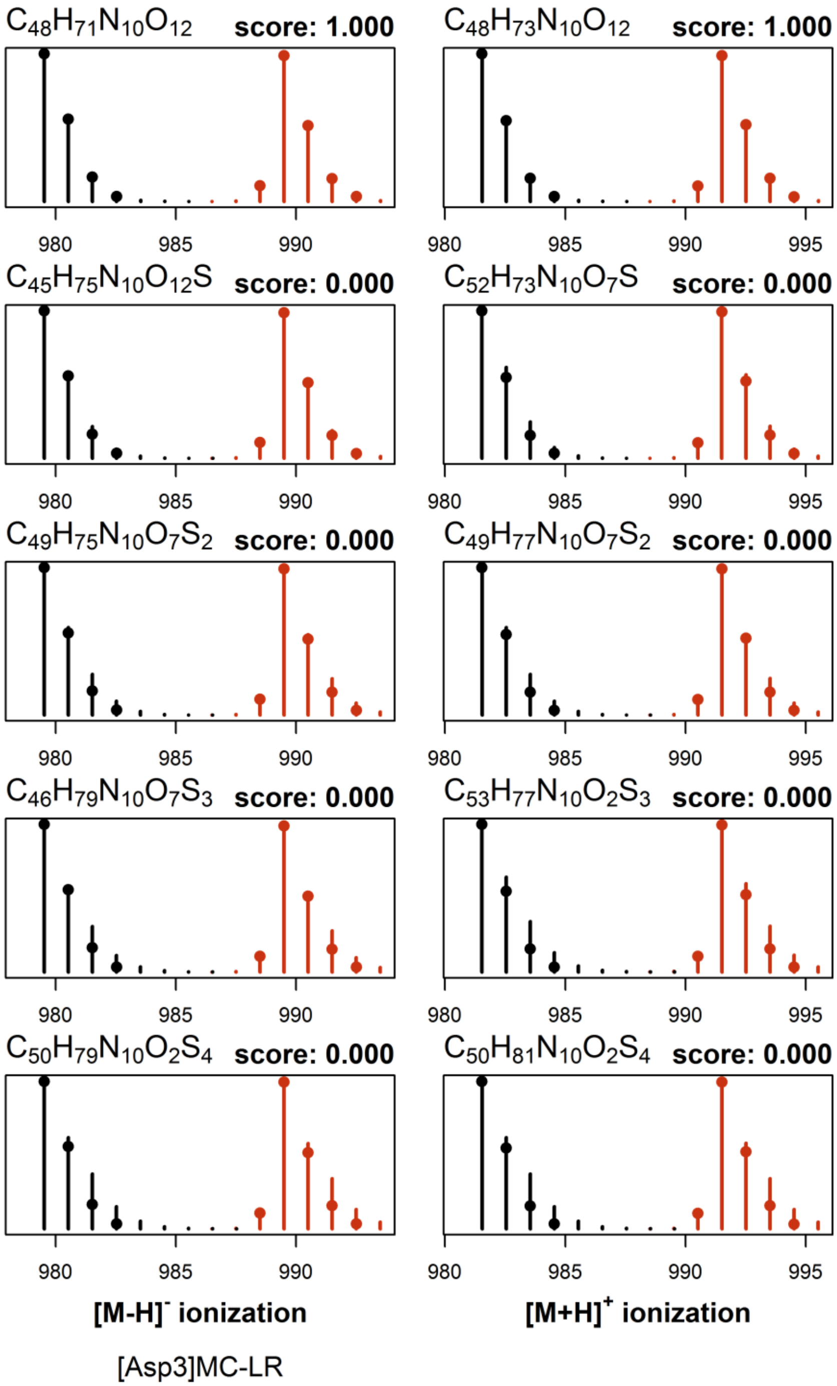

Figure S44. Most probable elemental compositions for [D-Asp[3]]MC-LR (**4**) based on full-scan LC–MS (method B) data for normalized ^{15}N-labelled (red) and natural abundance (black) cultures in negative (left) and positive (right) ionization modes using the NRC Molecular Formula Calculator. Candidate formulae and their scores are shown above the pairs of spectra in each panel, with the measured *m*/*z* and intensities indicated by the circles and the calculated values shown with vertical lines.

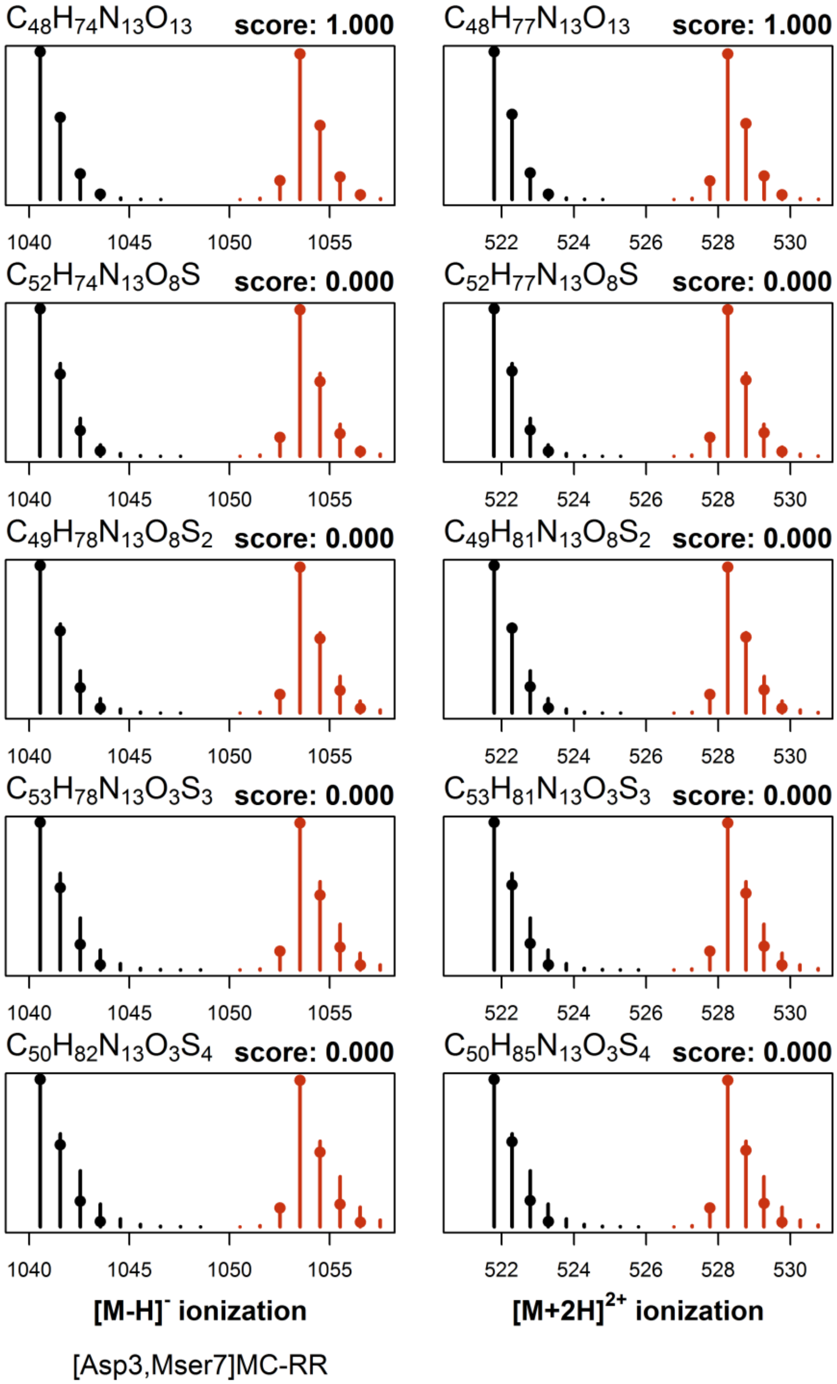

Figure S45. Most probable elemental compositions for [D-Asp[3],Mser[7]]MC-RR (**11**) based on full-scan LC–MS (method B) data for normalized ^{15}N-labelled (red) and natural abundance (black) cultures in negative (left) and positive (right) ionization modes using the NRC Molecular Formula Calculator. Candidate formulae and their scores are shown above the pairs of spectra in each panel, with the measured *m*/*z* and intensities indicated by the circles and the calculated values shown with vertical lines.

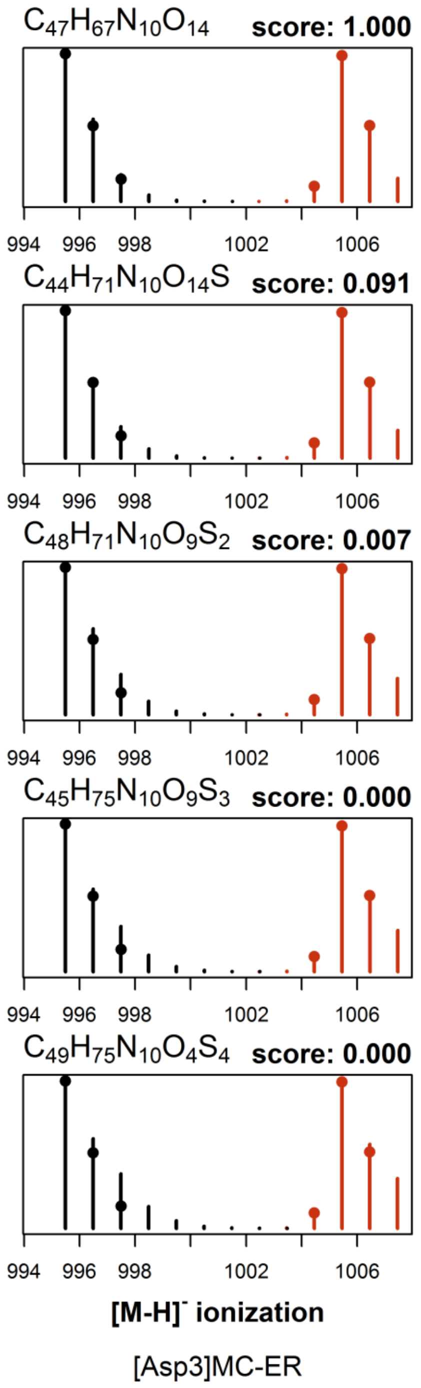

Figure S46. Most probable elemental compositions for [D-Asp[3]]MC-ER (**12**) based on full-scan LC–MS (method B) data for normalized ^{15}N-labelled (red) and natural abundance (black) cultures in negative (left) ionization mode using the NRC Molecular Formula Calculator. Candidate formulae and their scores are shown above the pairs of spectra in each panel, with the measured *m*/*z* and intensities indicated by the circles and the calculated values shown with vertical lines.

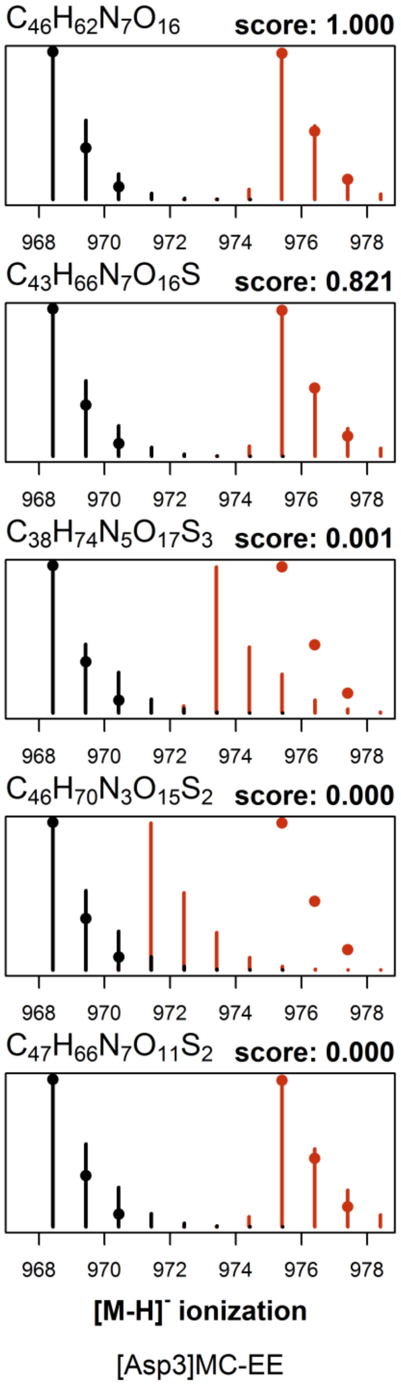

Figure S47. Most probable elemental compositions for [D-Asp[3]]MC-EE (**13**) based on full-scan LC–MS (method B) data for normalized ^{15}N-labelled (red) and natural abundance (black) cultures in negative (left) ionization mode using the NRC Molecular Formula Calculator. Candidate formulae and their scores are shown above the pairs of spectra in each panel, with the measured *m*/*z* and intensities indicated by the circles and the calculated values shown with vertical lines.

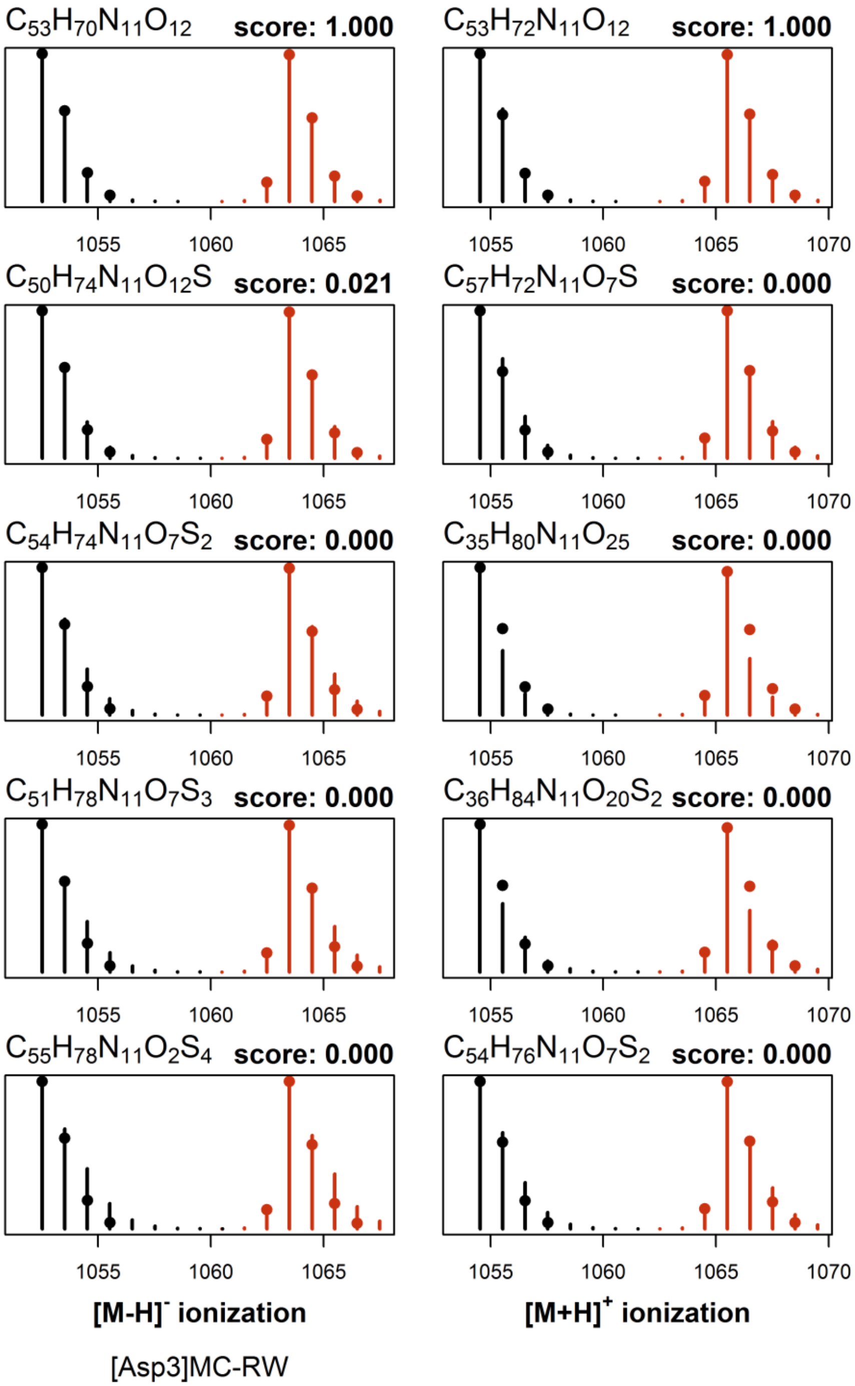

Figure S48. Most probable elemental compositions for [D-Asp^3]MC-RW (**14**) based on full-scan LC–MS (method B) data for normalized ^{15}N-labelled (red) and natural abundance (black) cultures in negative (left) and positive (right) ionization modes using the NRC Molecular Formula Calculator. Candidate formulae and their scores are shown above the pairs of spectra in each panel, with the measured *m*/*z* and intensities indicated by the circles and the calculated values shown with vertical lines.

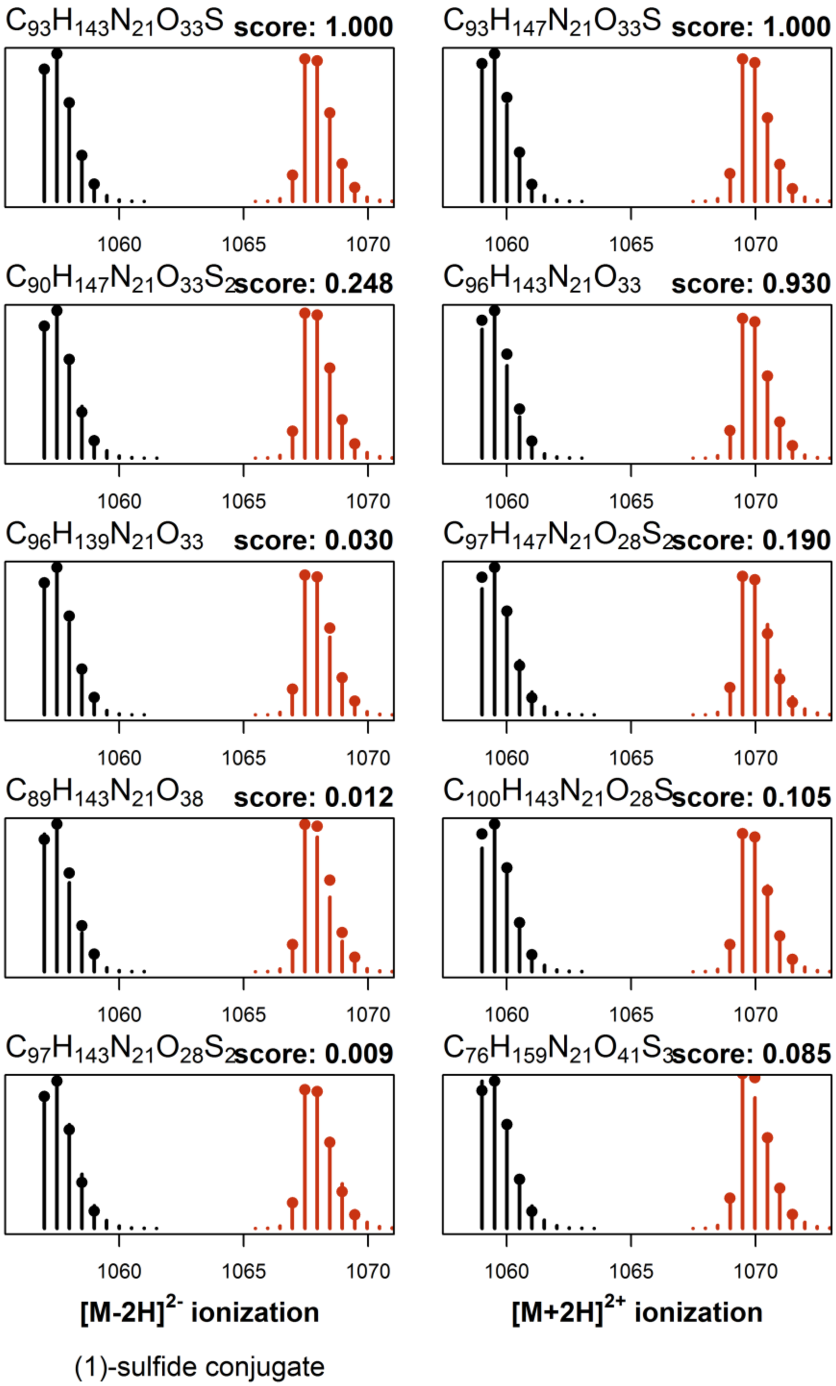

Figure S49. Most probable elemental compositions for the sulfide conjugate of [D-Asp^{3}]MC-RR (**15**) based on full-scan LC–MS (method B) data for normalized ^{15}N-labelled (red) and natural abundance (black) cultures in negative (left) and positive (right) ionization modes using the NRC Molecular Formula Calculator. Candidate formulae and their scores are shown above the pairs of spectra in each panel, with the measured *m*/*z* and intensities indicated by the circles and the calculated values shown with vertical lines.

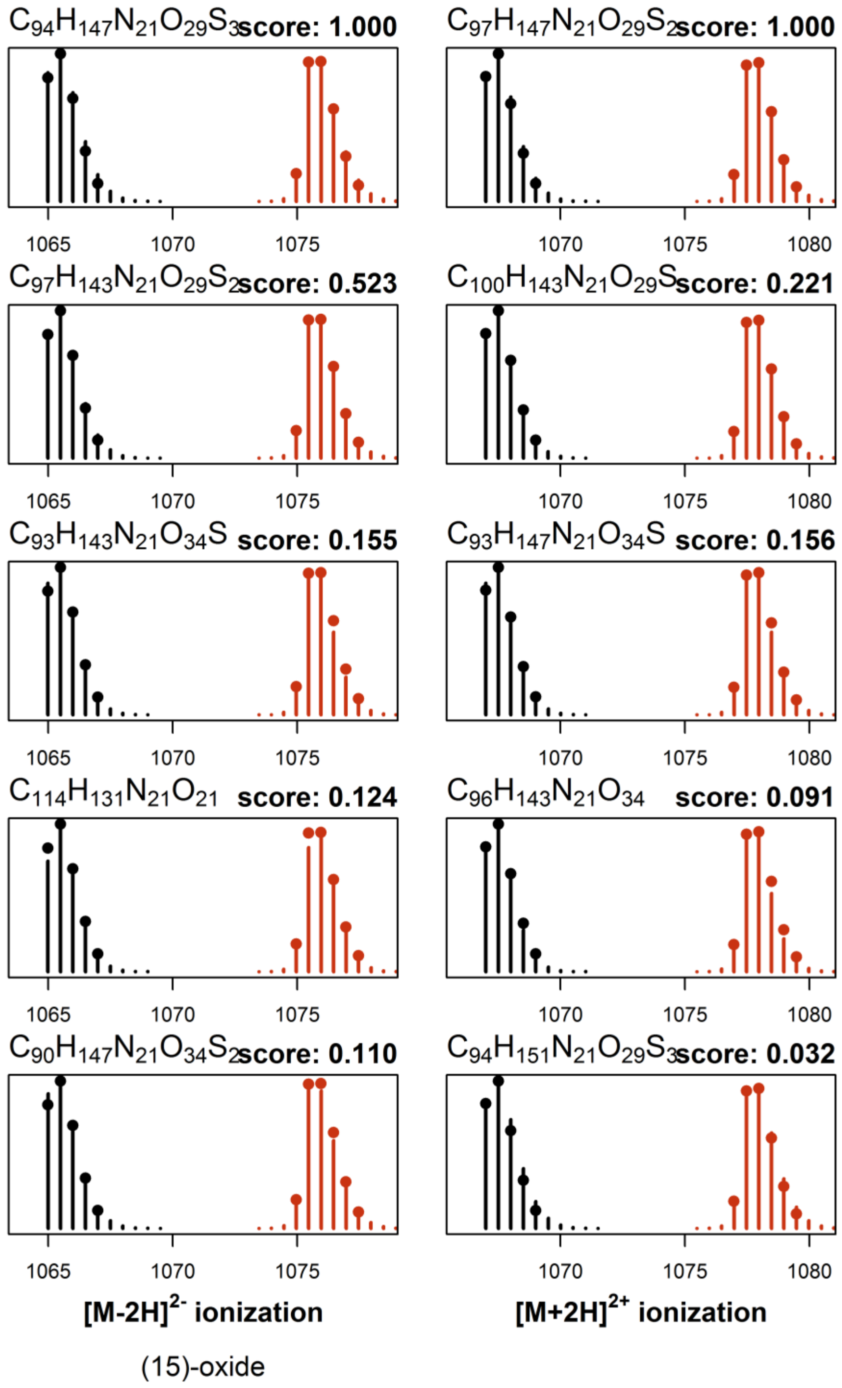

Figure S50. Most probable elemental compositions for the sulfoxide conjugate of [D-Asp[3]]MC-RR (**16**) (i.e. **15**-oxide) based on full-scan LC–MS (method B) data for normalized ^{15}N-labelled (red) and natural abundance (black) cultures in negative (left) and positive (right) ionization modes using the NRC Molecular Formula Calculator. Candidate formulae and their scores are shown above the pairs of spectra in each panel, with the measured *m*/*z* and intensities indicated by the circles and the calculated values shown with vertical lines.

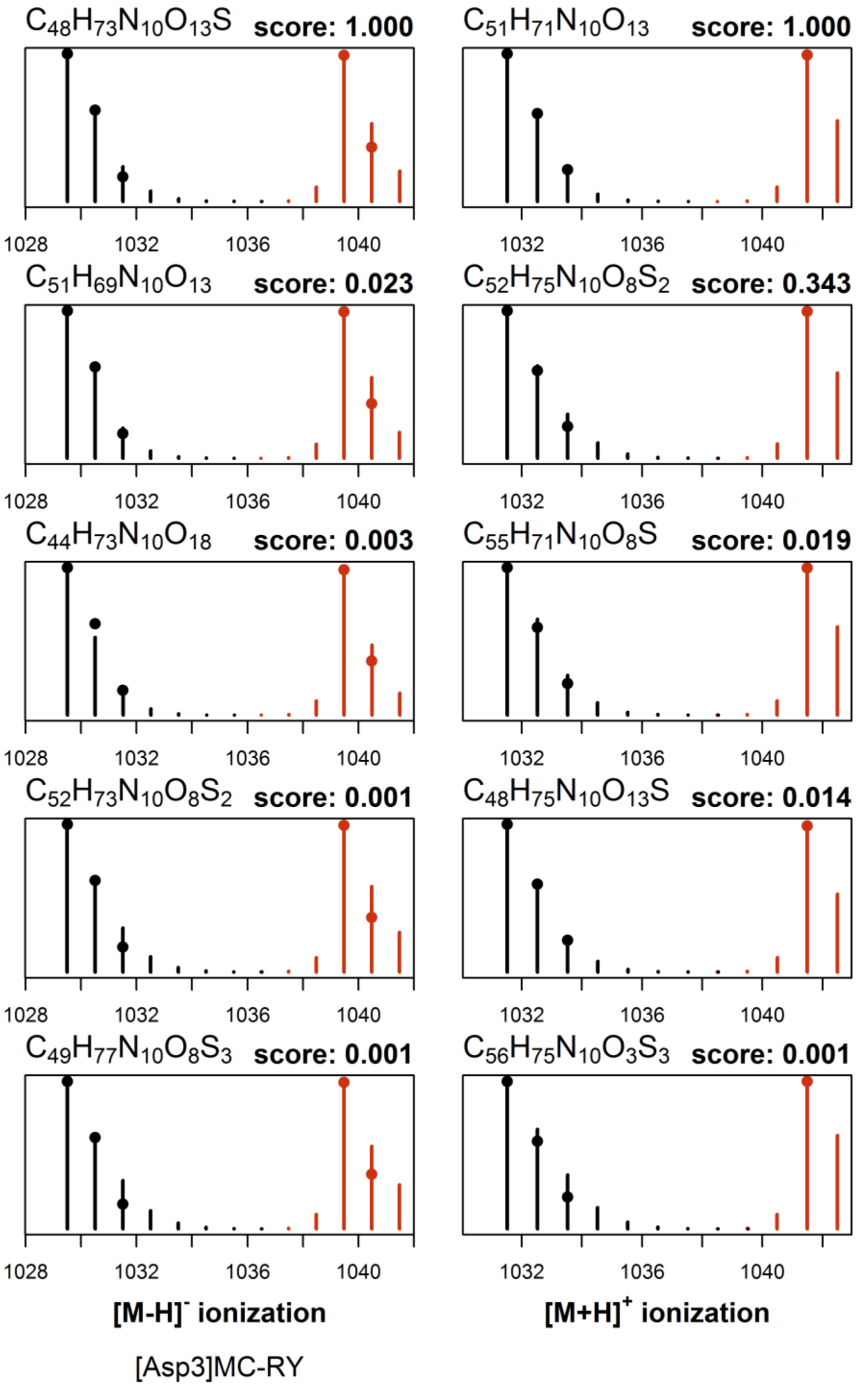

Figure S51. Most probable elemental compositions for [D-Asp^3]MC-RY (**17**) based on full-scan LC–MS (method B) data for normalized ^{15}N-labelled (red) and natural abundance (black) cultures in negative (left) and positive (right) ionization modes using the NRC Molecular Formula Calculator. Candidate formulae and their scores are shown above the pairs of spectra in each panel, with the measured *m*/*z* and intensities indicated by the circles and the calculated values shown with vertical lines.

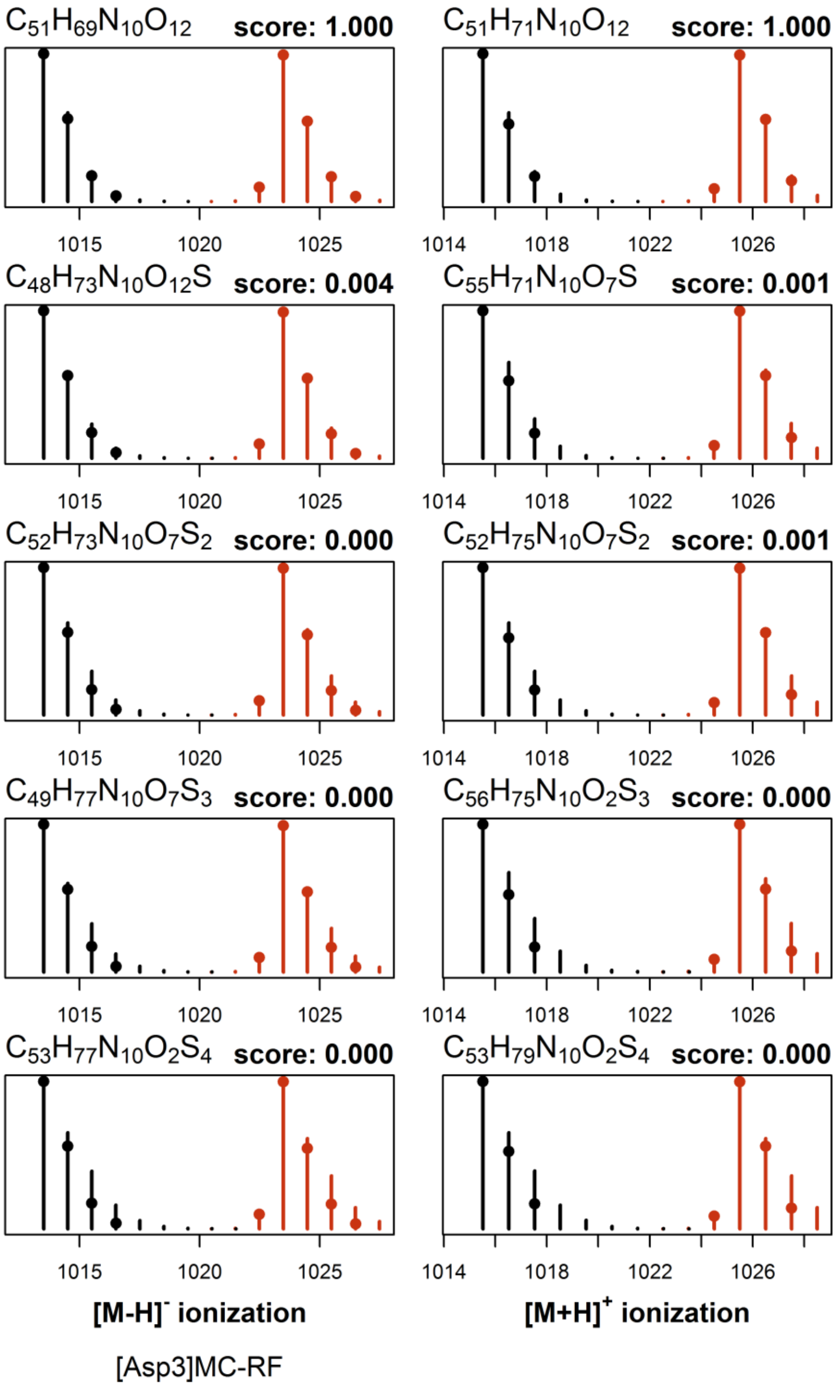

Figure S52. Most probable elemental compositions for [D-Asp^3]MC-RF (**18**) based on full-scan LC–MS (method B) data for normalized ^{15}N-labelled (red) and natural abundance (black) cultures in negative (left) and positive (right) ionization modes using the NRC Molecular Formula Calculator. Candidate formulae and their scores are shown above the pairs of spectra in each panel, with the measured *m*/*z* and intensities indicated by the circles and the calculated values shown with vertical lines.

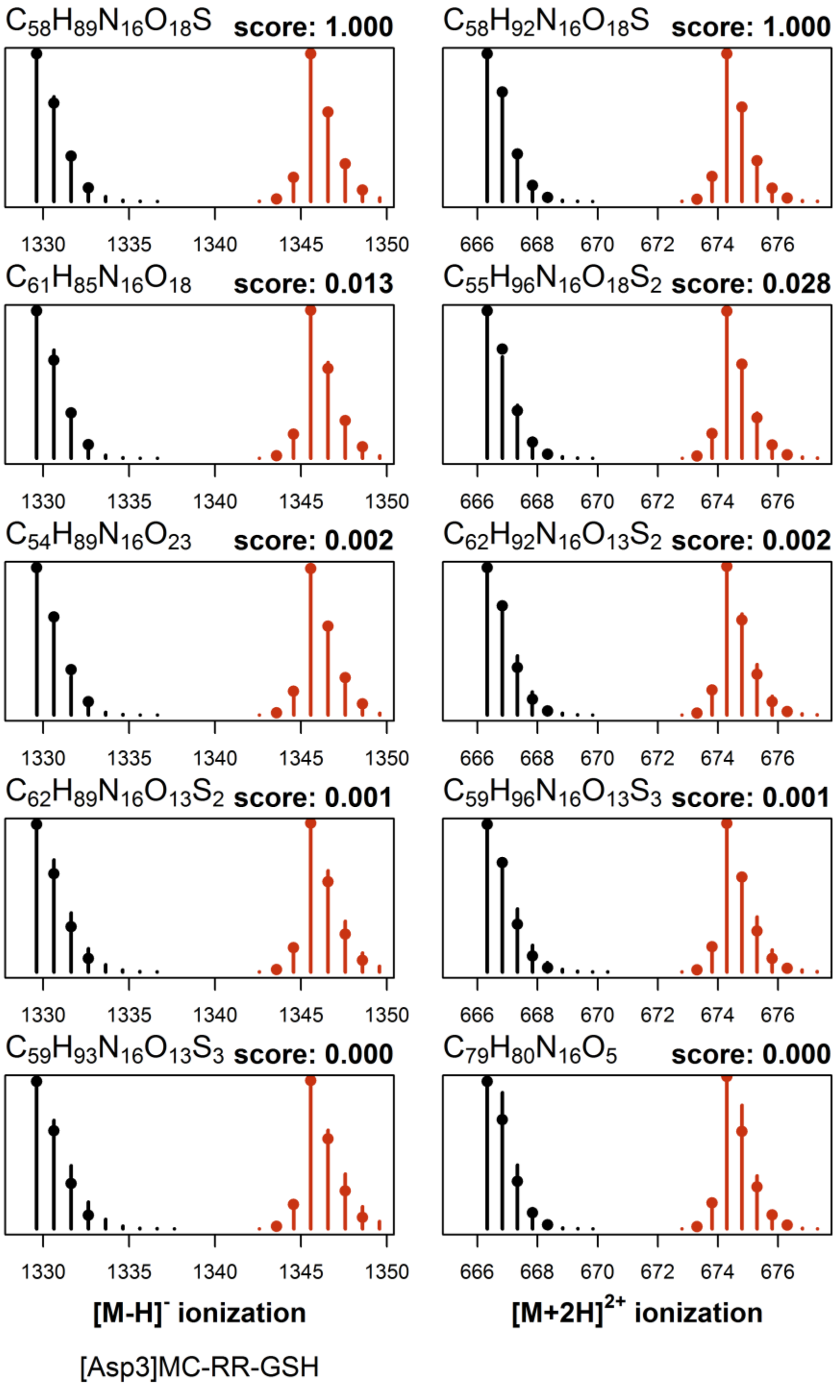

Figure S53. Most probable elemental compositions for the glutathione conjugate of [D-Asp[3]]MC-RR (**19**) based on full-scan LC–MS (method B) data for normalized ^{15}N-labelled (red) and natural abundance (black) cultures in negative (left) and positive (right) ionization modes using the NRC Molecular Formula Calculator. Candidate formulae and their scores are shown above the pairs of spectra in each panel, with the measured *m*/*z* and intensities indicated by the circles and the calculated values shown with vertical lines.

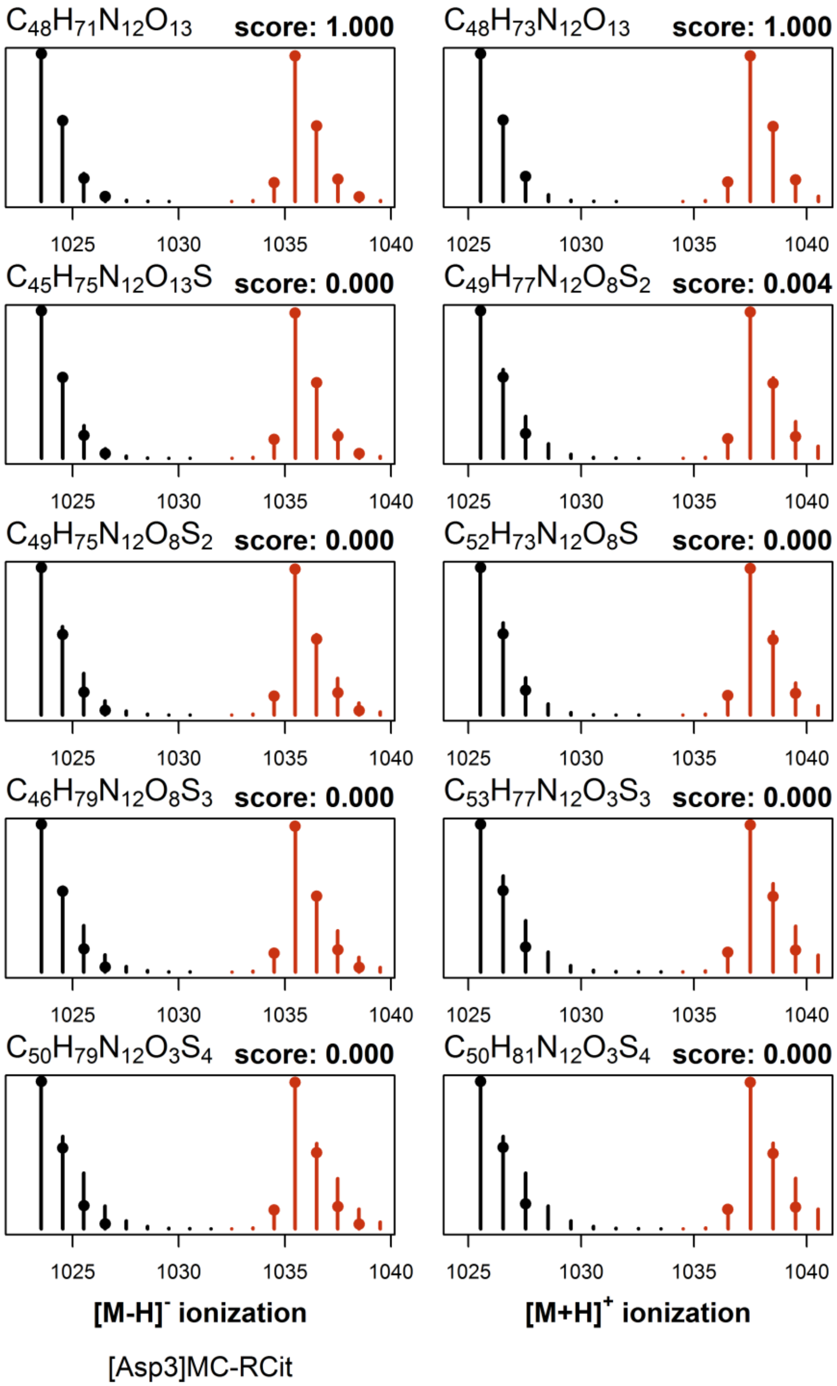

Figure S54. Most probable elemental compositions for [D-Asp[3]]MC-RCit (**20**) based on full-scan LC–MS (method B) data for normalized ^{15}N-labelled (red) and natural abundance (black) cultures in negative (left) and positive (right) ionization modes using the NRC Molecular Formula Calculator. Candidate formulae and their scores are shown above the pairs of spectra in each panel, with the measured *m*/*z* and intensities indicated by the circles and the calculated values shown with vertical lines.

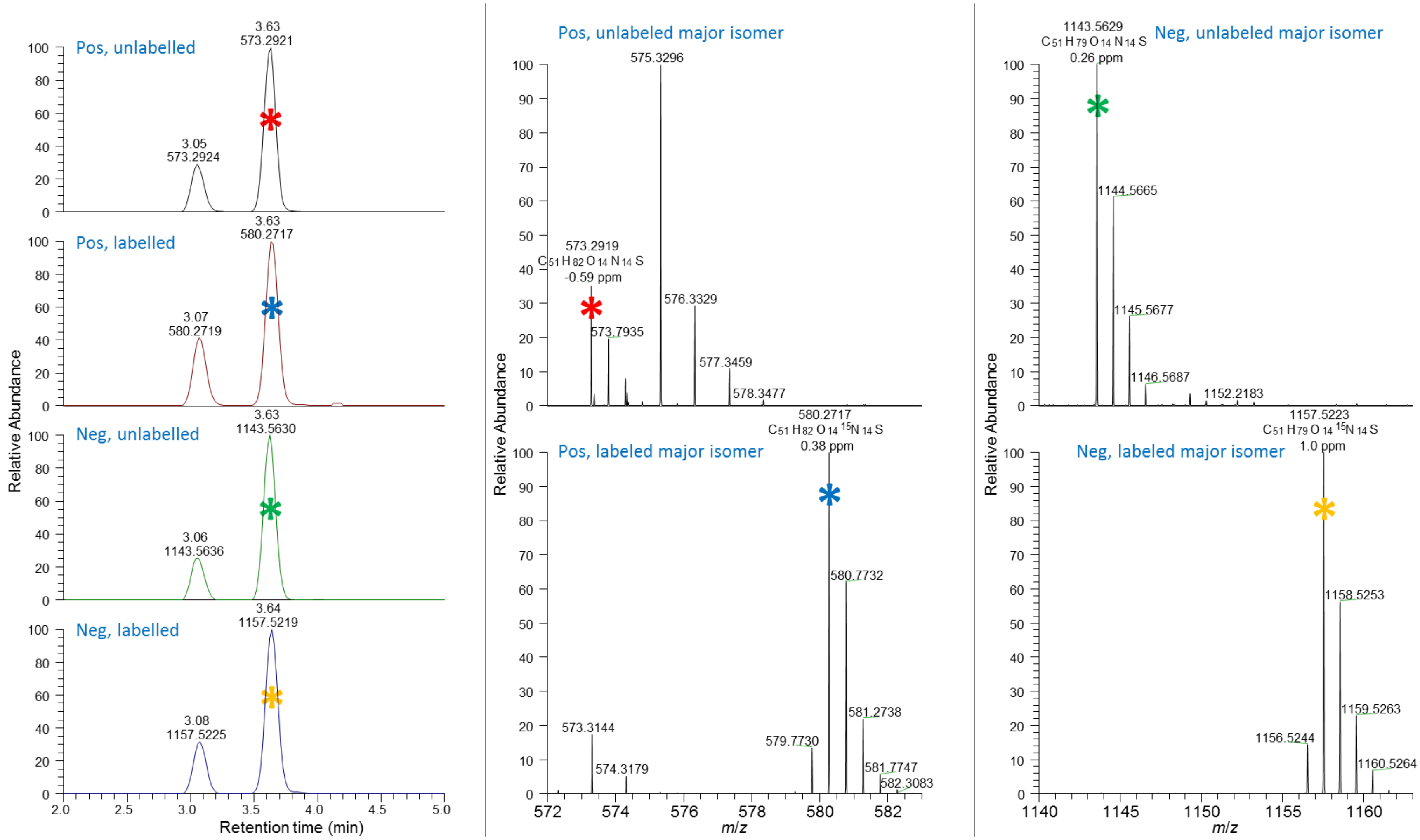

Figure S55. Left, LC-HRMS (method B) chromatograms of the [D-Asp[3]]MC-RR cysteine conjugate in unlabelled and ^{15}N-labelled cultures of *P. prolifica* NICA-CYA 544 in positive and negative modes extracted at *m/z* for $[M + 2H]^{2+}$ and $[M – H]^{–}$. Note the presence of a major and a minor isomer. Centre, positive mode mass spectra of the major isomer in the unlabelled (top) and ^{15}N-labelled cultures (bottom). Right, negative mode mass spectra of the major isomer in the unlabelled (top) and ^{15}N-labelled cultures (bottom). Analysis of the isotope patterns with the NRC Molecular Formula Calculator is shown in Figure S57.

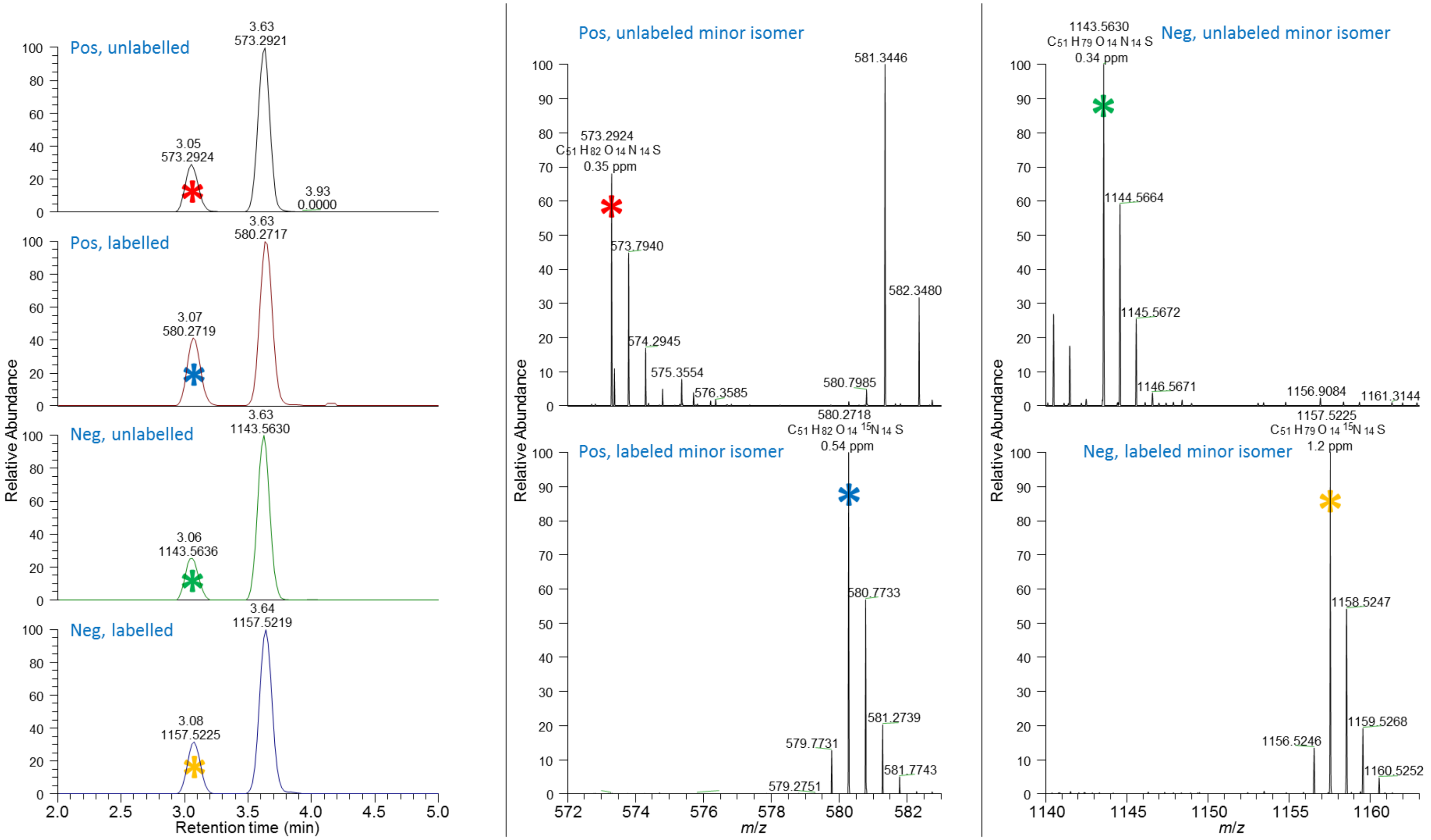

Figure S56. Left, LC-HRMS (method B) chromatograms of the [D-Asp[3]]MC-RR cysteine conjugate in unlabelled and ^{15}N-labelled cultures of *P. prolifica* NICA-CYA 544 in positive and negative modes extracted at *m/z* for $[M + 2H]^{2+}$ and $[M - H]^{-}$. Note the presence of a major and a minor isomer. Centre, positive mode mass spectra of the minor isomer in the unlabelled (top) and ^{15}N-labelled cultures (bottom). Right, negative mode mass spectra of the minor isomer in the unlabelled (top) and ^{15}N-labelled cultures (bottom).

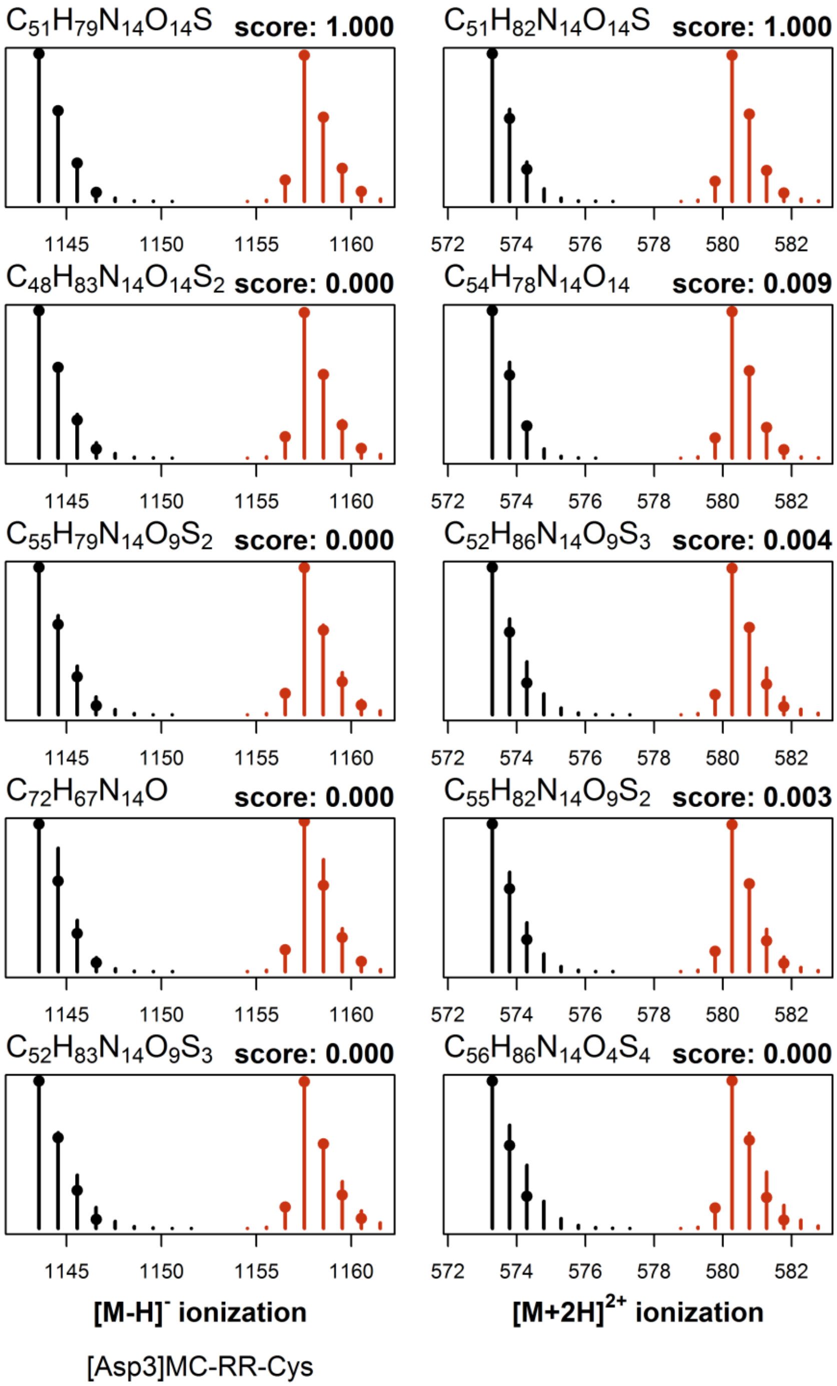

Figure S57. Most probable elemental compositions for the major isomer of the cysteine conjugate of [D-Asp^{3}]MC-RR based on the full-scan LC–MS (method B) data shown in Figure S55. The panels show normalized ^{15}N-labelled (red) and natural abundance (black) mass spectra for cultures in negative (left) and positive (right) ionization modes using the NRC Molecular Formula Calculator. Candidate formulae and their scores are shown above the pairs of spectra in each panel, with the measured *m*/*z* and intensities indicated by the circles and the calculated values shown with vertical lines.

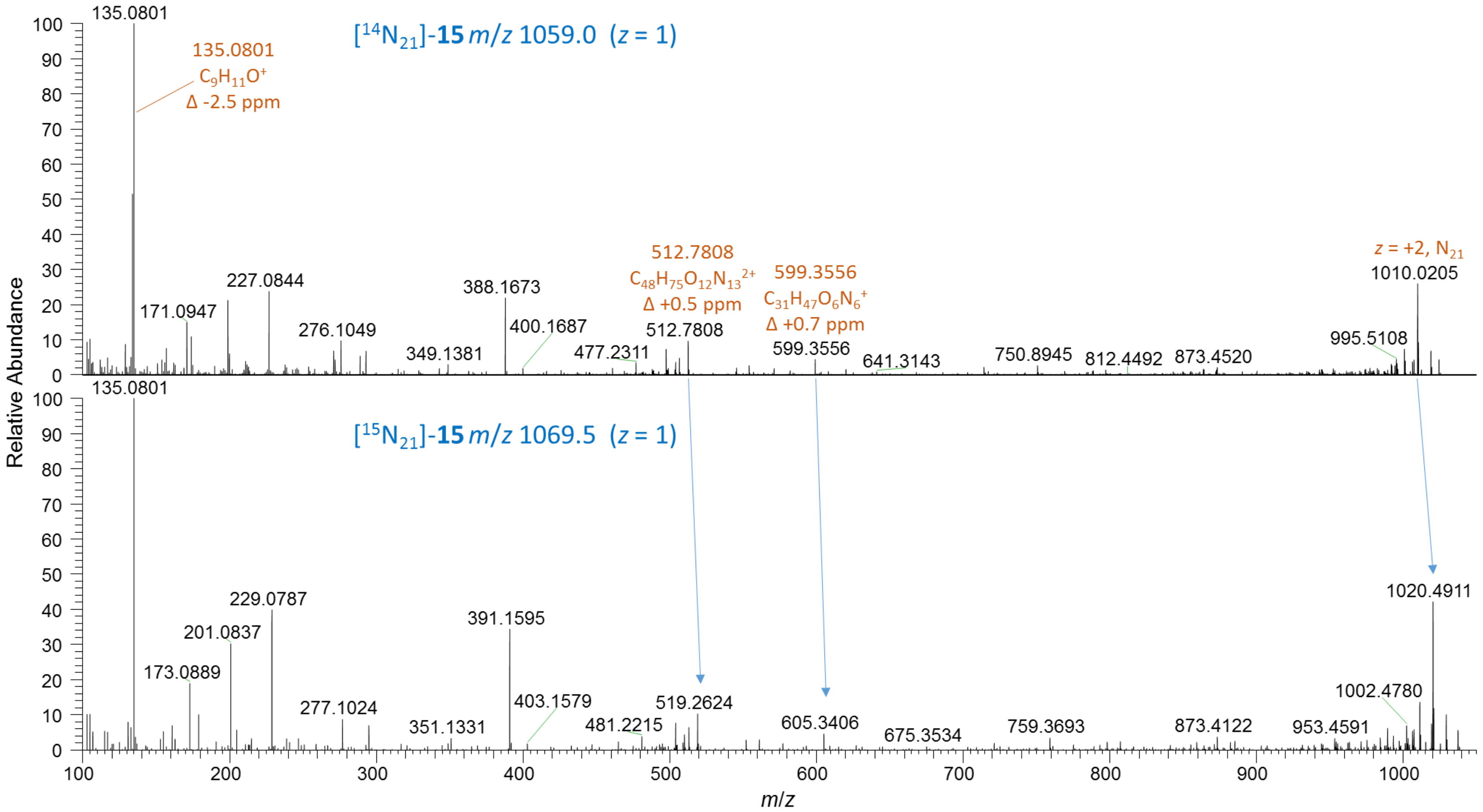

Figure S58. LC–HRMS/MS PRM spectra (method B) of $[M + 2H]^{2+}$ of the sulfide conjugate of the: top, unlabelled [D-Asp^3]MC-RR (**15**) at *m/z* 1059.0 recorded with setting *z* =1; bottom, ^{15}N-labelled **15** at *m/z* 1069.5. Note the characteristic product ions indicating the presence of [D-Asp^3]MC-RR (**1**) in conjugate-**15**.

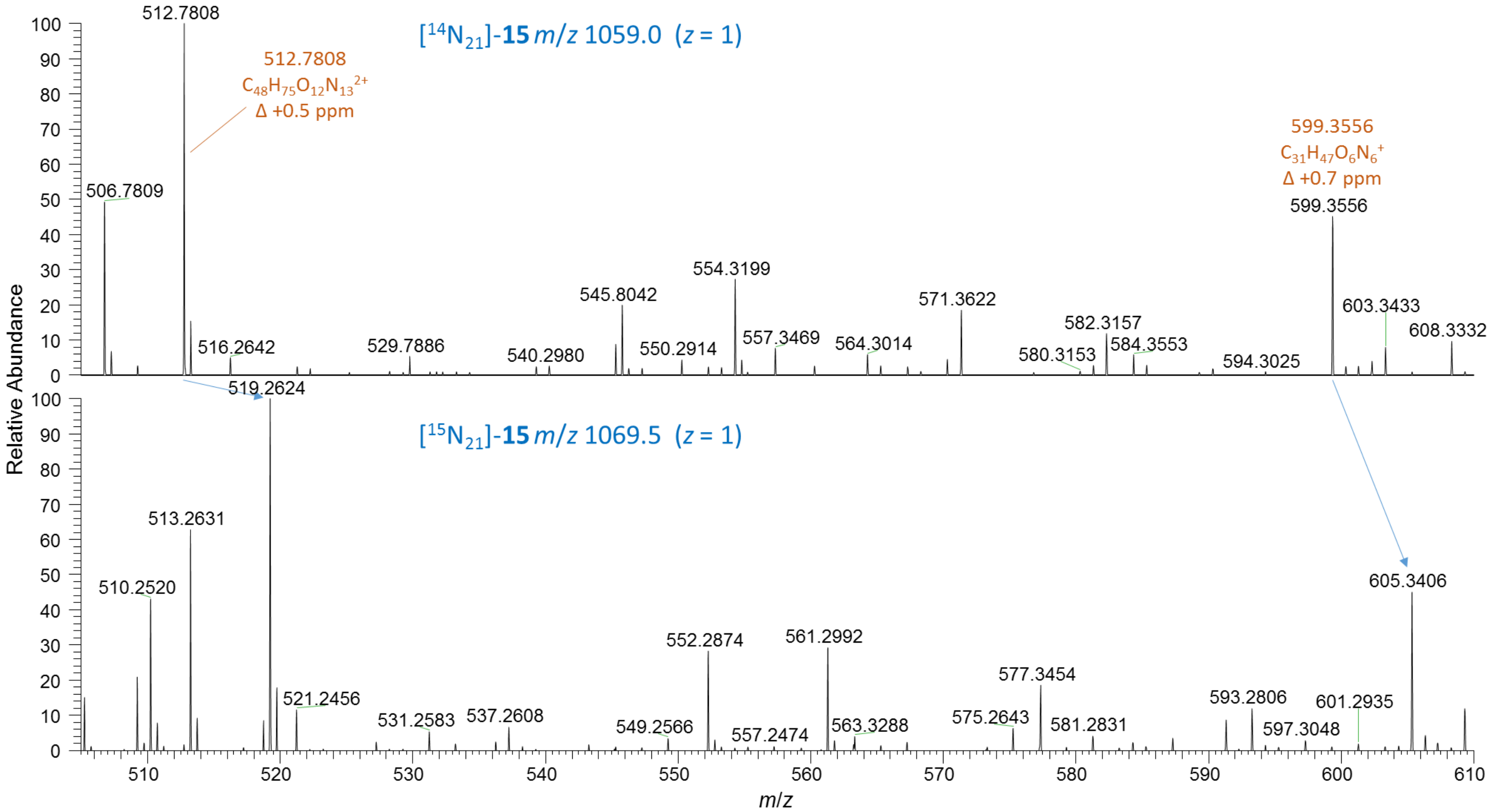

Figure S59. Expansion of LC–HRMS/MS PRM spectra (method B) of $[M + 2H]^{2+}$ of the sulfide conjugate (see Figure S58) of the: top, unlabelled [D-Asp^3]MC-RR (**15**) at *m*/*z* 1059.0 recorded with setting *z* =1; bottom, ^{15}N-labelled **15** at *m*/*z* 1069.5. Note the characteristic product ions indicating the presence of [D-Asp^3]MC-RR (**1**) in conjugate-**15**.

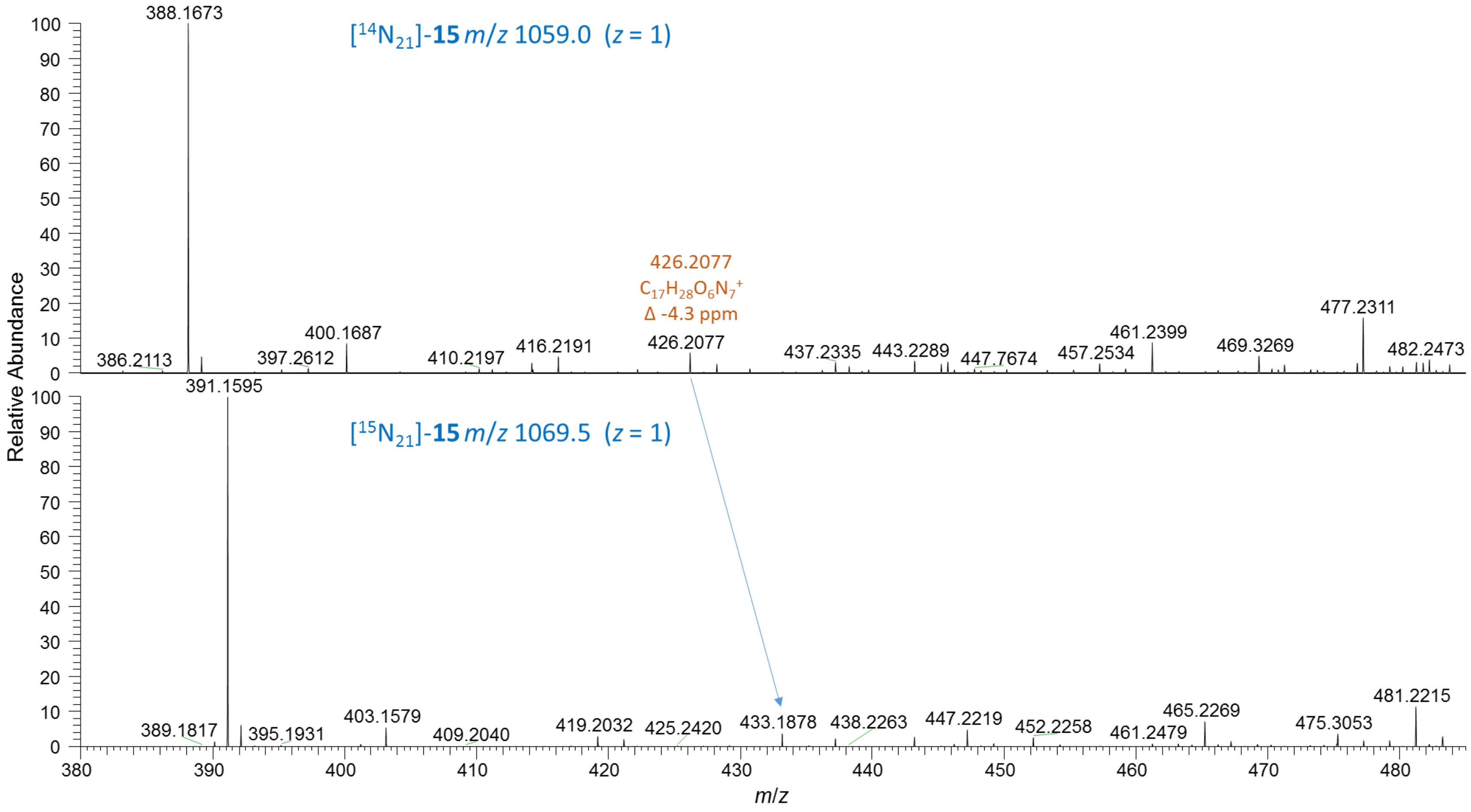

Figure S60. Expansion of LC–HRMS/MS PRM spectra (method B) of $[M + 2H]^{2+}$ of the sulfide conjugate (see Figure S58) of the: top, unlabelled [D-Asp^3]MC-RR (**15**) at *m/z* 1059.0 recorded with setting *z* =1; bottom, ^{15}N-labelled **15** at *m/z* 1069.5. Note the characteristic product ions indicating the presence of [D-Asp^3]MC-RR (**1**) in conjugate-**15**.

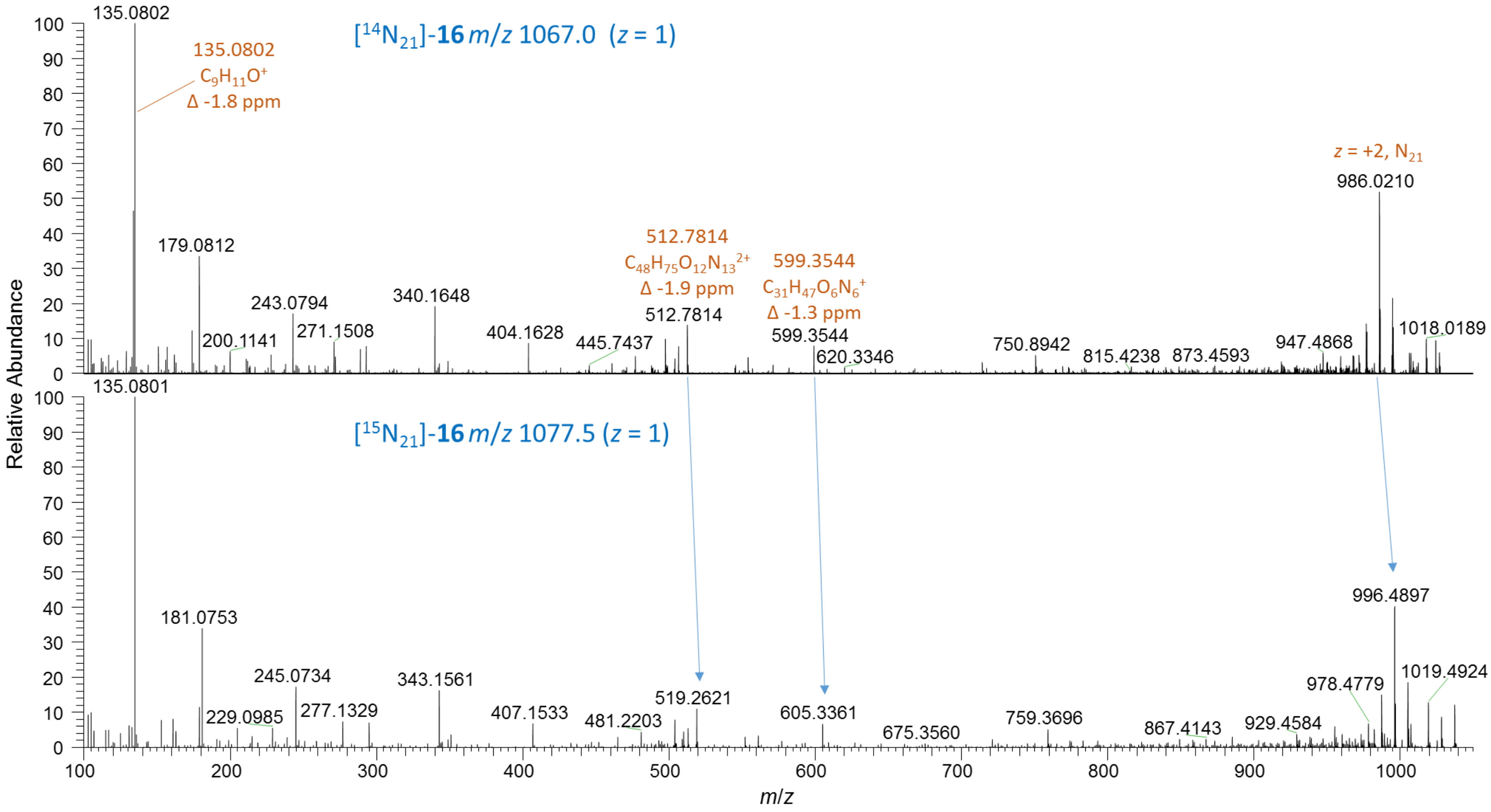

Figure S61. LC–HRMS/MS PRM spectra (method B) of [M + 2H]$^{2+}$ of the sulfoxide conjugate of the: top, unlabelled [D-Asp^3]MC-RR (**16**) at *m/z* 1067.0 recorded with setting *z* =1; bottom, ^{15}N-labelled **16** at *m/z* 1077.5. Note the characteristic product ions indicating the presence of [D-Asp^3]MC-RR (**1**) in conjugate-**16**.

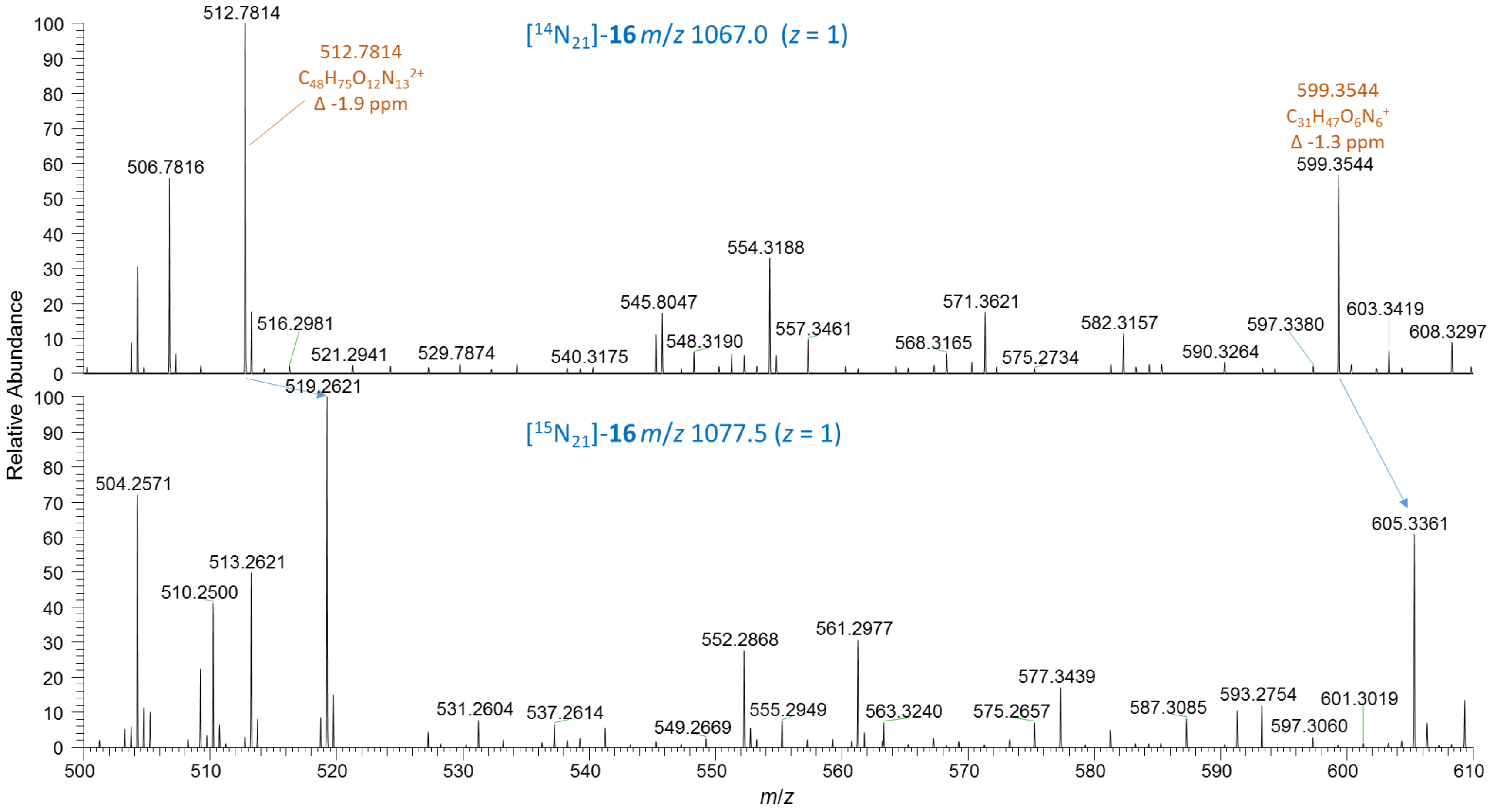

Figure S62. Expansion of LC–HRMS/MS PRM spectra (method B) of $[M + 2H]^{2+}$ of the sulfoxide conjugate (see Figure S61) of the: top, unlabelled [D-Asp^3]MC-RR (**16**) at *m/z* 1067.0 recorded with setting *z* =1; bottom, ^{15}N-labelled **15** at *m/z* 1077.5. Note the characteristic product ions indicating the presence of [D-Asp^3]MC-RR (**1**) in conjugate-**16**.

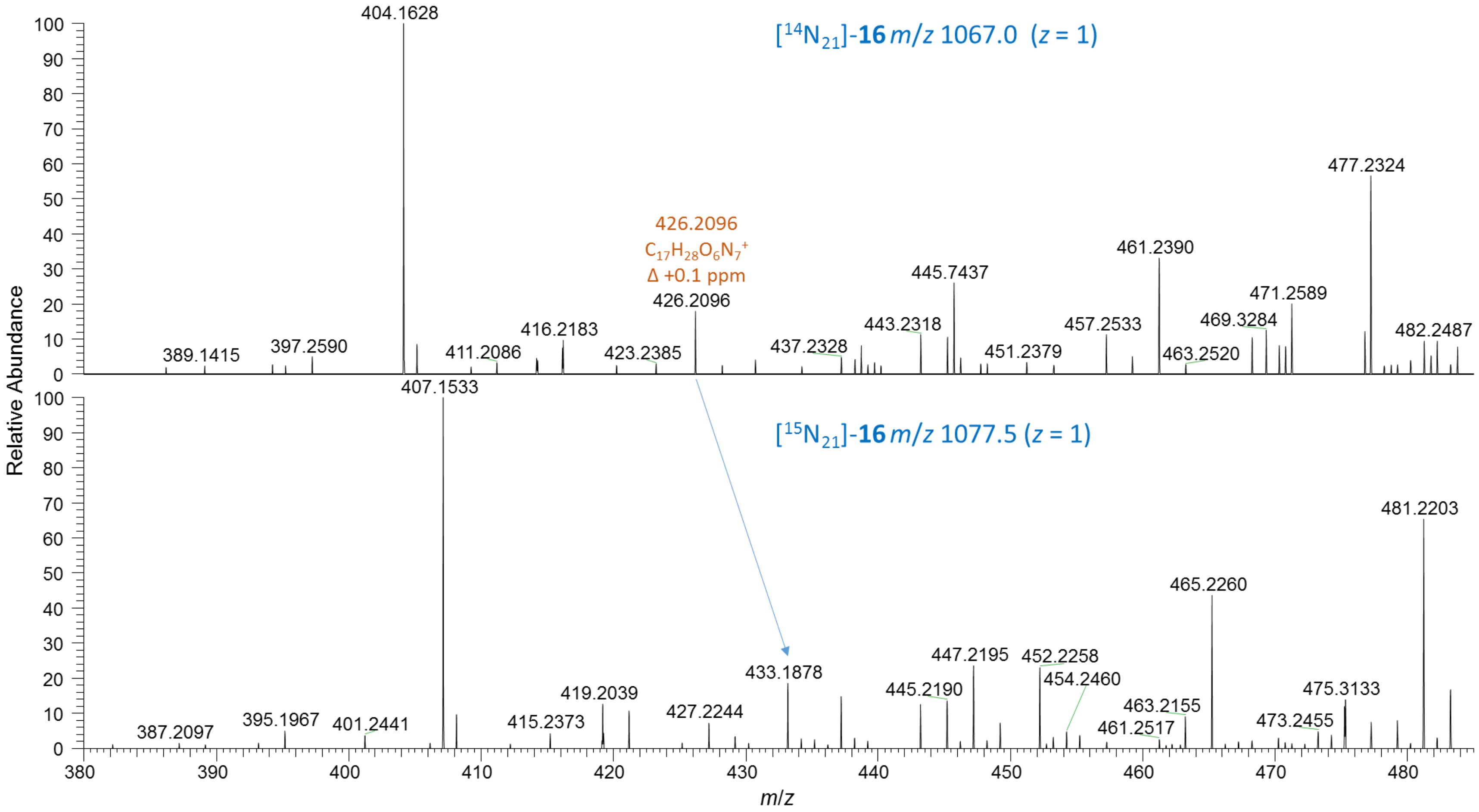

Figure S63. Expansion of LC–HRMS/MS PRM spectra (method B) of $[M + 2H]^{2+}$ of the sulfoxide conjugate (see Figure S61) of the: top, unlabelled [D-Asp^3]MC-RR (**16**) at *m*/*z* 1067.0 recorded with setting *z* =1; bottom, ^{15}N-labelled **15** at *m*/*z* 1077.5. Note the characteristic product ions indicating the presence of [D-Asp^3]MC-RR (**1**) in conjugate-**16**.

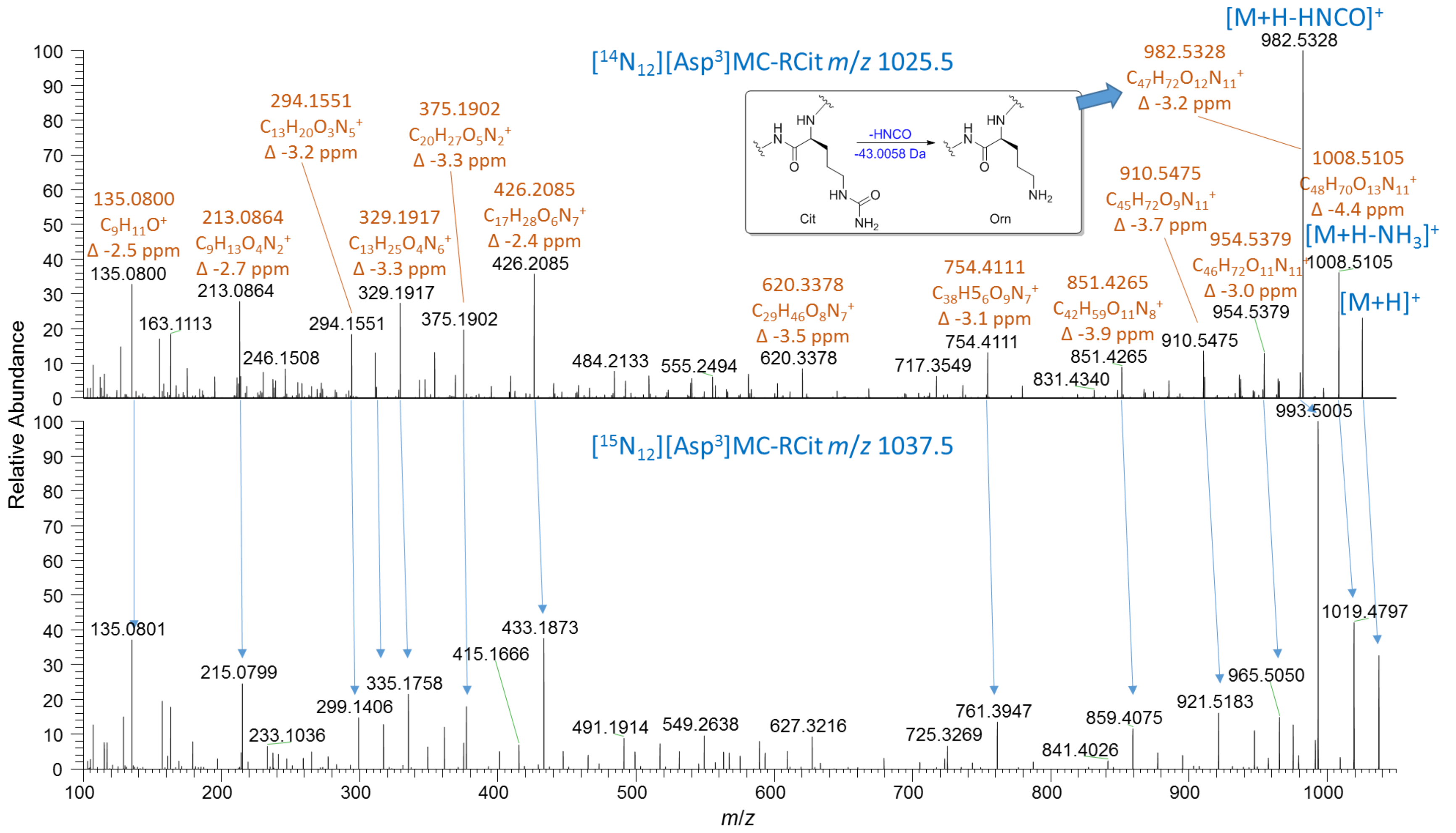

Figure S64. LC–HRMS/MS PRM spectra (method B) of $[M + H]^+$ of: top, unlabelled [D-Asp^3]MC-RCit (**20**) at *m/z* 1025.5, and; bottom, ^{15}N-labelled **20** at *m/z* 1037.5. Note the prominent neutral loss of isocyanic acid (HNCO, 43.0058 Da) in unlabelled **20**, which characteristic of citrulline residues in peptides.[4]

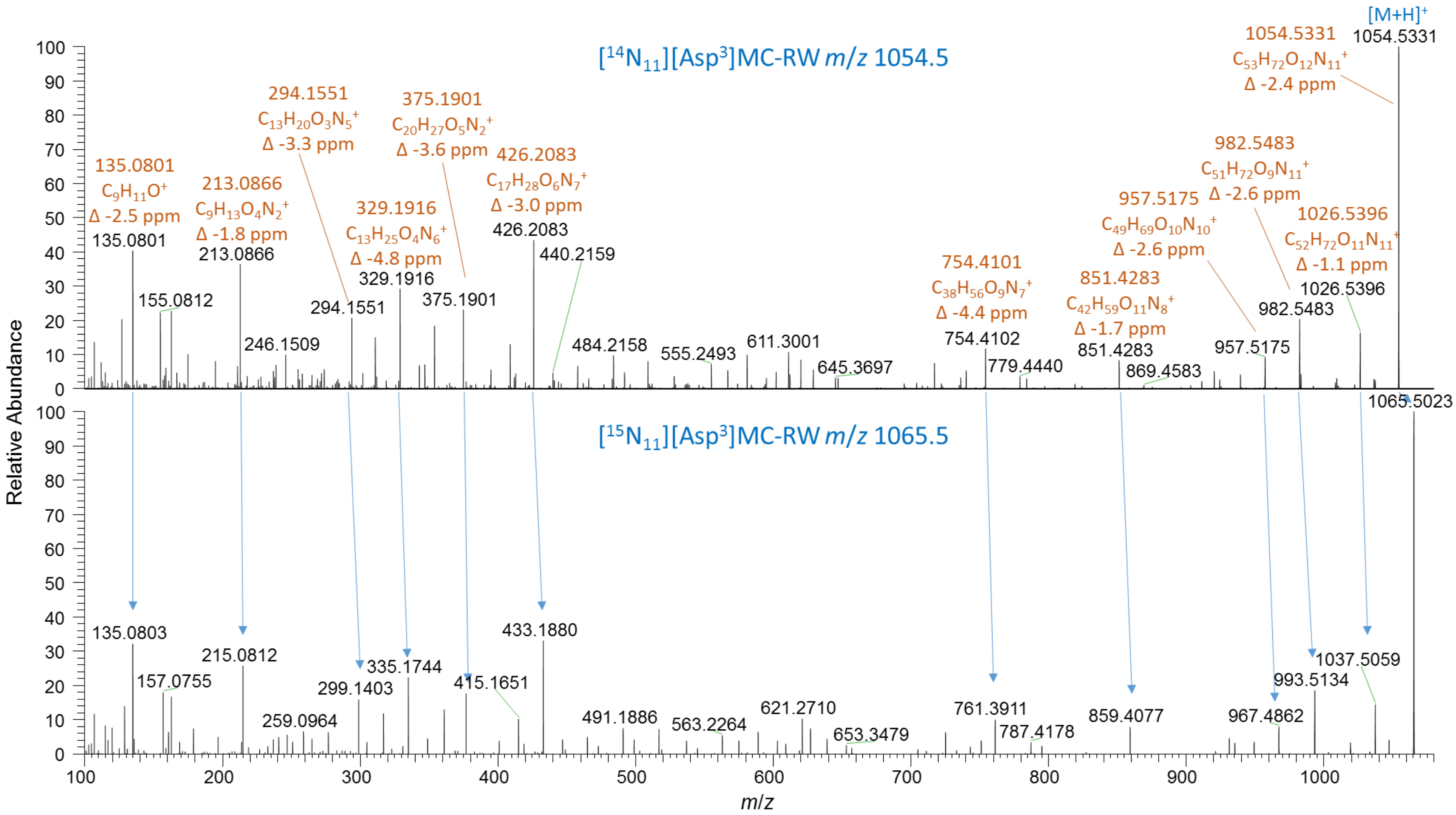

Figure S65. LC–HRMS/MS PRM spectra (method B) of $[M + H]^+$ of: top, unlabelled [D-Asp^3]MC-RW (**14**) at *m/z* 1054.5, and; bottom, ^{15}N-labelled **14** at *m/z* 1069.5.

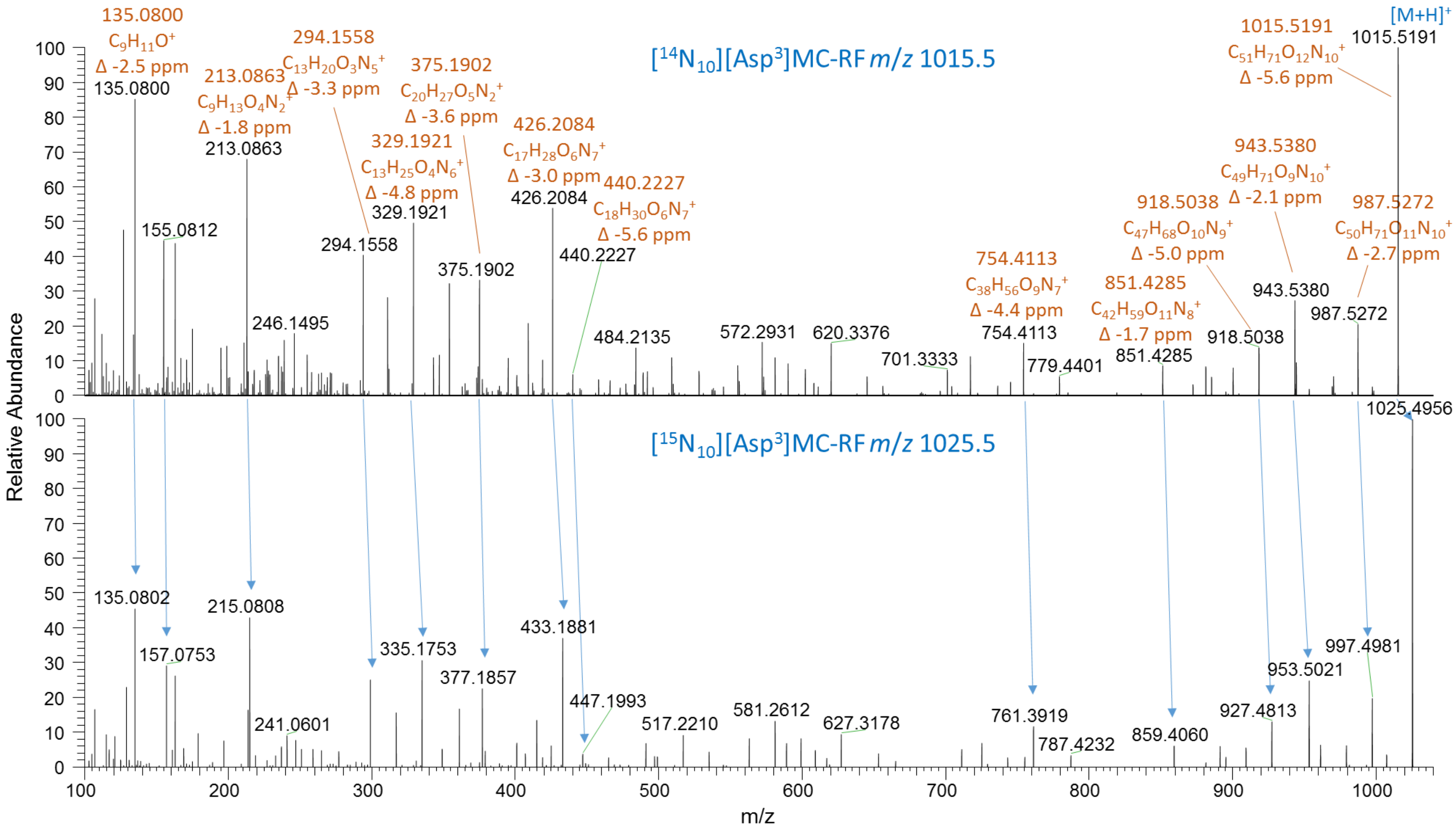

Figure S66. LC–HRMS/MS PRM spectra (method B) of $[M + H]^+$ of: top, unlabelled [D-Asp^3]MC-RF (**18**) at *m*/*z* 1015.5, and; bottom, ^{15}N-labelled **18** at *m*/*z* 1025.5.

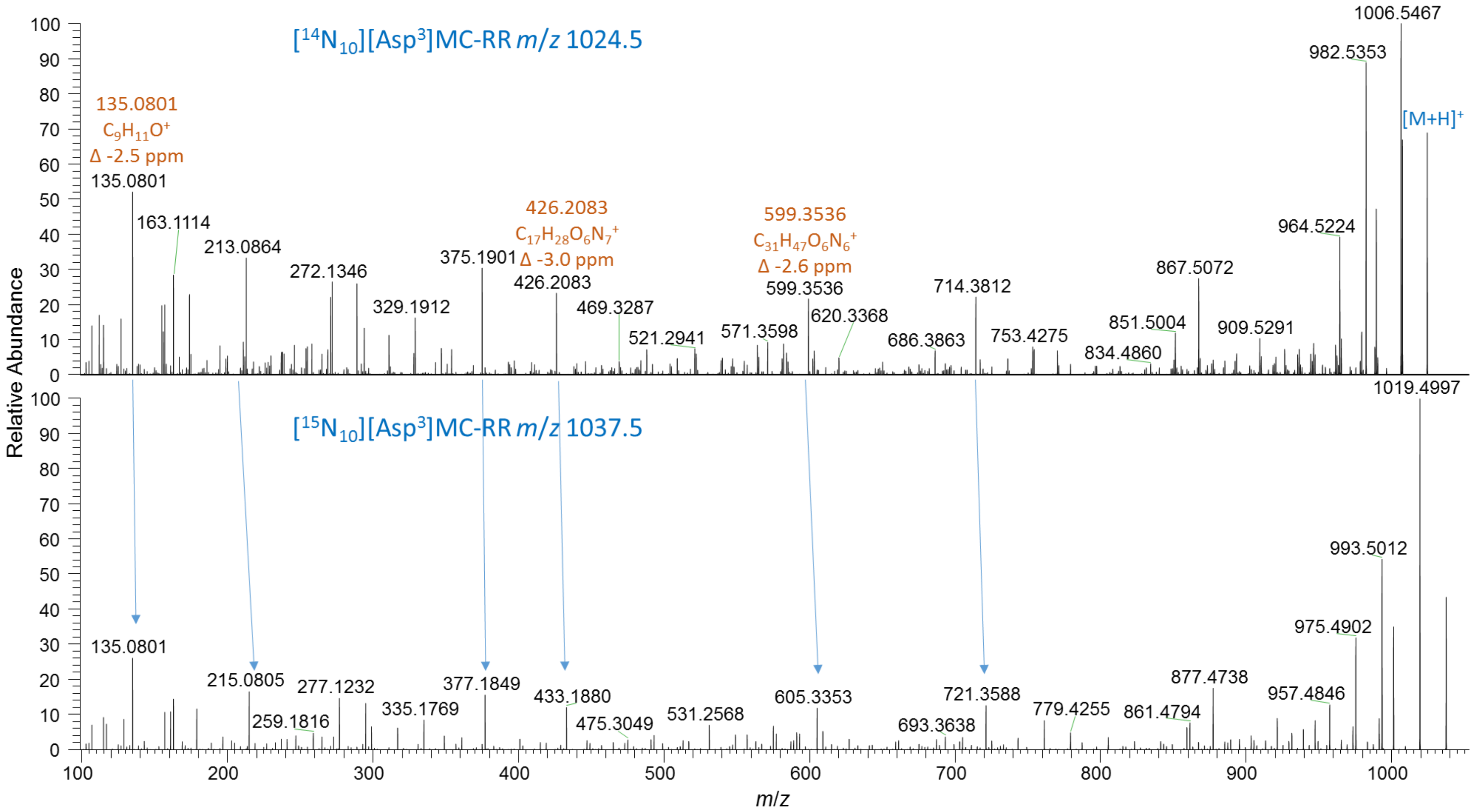

Figure S67. LC–HRMS/MS PRM spectra (method B) of $[M + H]^+$ of: top, unlabelled [D-Asp^3]MC-RR (**1**) at *m/z* 1024.5, and; bottom, ^{15}N-labelled **1** at *m/z* 1037.5. Note the presence of the characteristic singly-charged product ions at *m/z* 135.0804 (derived from Adda^5), 426.2096 (derived from Mdha^7–Ala^1–Arg^2–Asp^3), and 599.3552 (derived from Arg^4–Adda^5–Glu^6) that were also observed in the MS/MS spectra of **15** and **16** (Figures S32–33 and S58–63).

Literature Cited

1. Yilmaz, Y.; Foss, A. J.; Miles, C. O.; Özen, M.; Demir, N.; Balci, M.; Beach, D. G., Comprehensive multi-technique approach reveals the high diversity of microcystins in field collections and an associated isolate of *Microcystis aeruginosa* from a Turkish lake. *Toxicon* **2019**, *167*, 87−100.
2. LeBlanc, P.; Merkley, N.; Thomas, K.; Lewis, N. I.; Békri, K.; LeBlanc Renaud, S.; Pick, F. R.; McCarron, P.; Miles, C. O.; Quilliam, M. A., Isolation and characterization of [D-Leu[1]]microcystin-LY from *Microcystis aeruginosa* CPCC-464. *Chem. Res. Toxicol.* **2019**, (submitted).
3. Miles, C. O.; Sandvik, M.; Nonga, H. E.; Rundberget, T.; Wilkins, A. L.; Rise, F.; Ballot, A., Identification of microcystins in a Lake Victoria cyanobacterial bloom using LC-MS with thiol derivatization. *Toxicon* **2013**, *70*, 21−31.
4. Hao, G.; Wang, D.; Gu, J.; Shen, Q.; Gross, S. S.; Wang, Y., Neutral loss of isocyanic acid in peptide CID spectra: a novel diagnostic marker for mass spectrometric identification of protein citrullination. *J. Am. Soc. Mass Spectrom.* **2009**, *20*, 723−727.
